# Synthesis, Docking, and In Vitro Anticoagulant Activity Assay of Hybrid Derivatives of Pyrrolo[3,2,1-*ij*]Quinolin-2(1*H*)-one as New Inhibitors of Factor Xa and Factor XIa

**DOI:** 10.3390/molecules25081889

**Published:** 2020-04-19

**Authors:** Nadezhda Novichikhina, Ivan Ilin, Anna Tashchilova, Alexey Sulimov, Danil Kutov, Irina Ledenyova, Mikhail Krysin, Khidmet Shikhaliev, Anna Gantseva, Ekaterina Gantseva, Nadezhda Podoplelova, Vladimir Sulimov

**Affiliations:** 1 Department of Organic Chemistry, Faculty of Chemistry, Voronezh State University, 1 Universitetskaya sq., 394018 Voronezh, Russia; podaneva_nadya@mail.ru (N.N.); irairachem@yandex.ru (I.L.); chocd261@chem.vsu.ru (K.S.); 2 Research Computing Center, Lomonosov Moscow State University, 119992 Moscow, Russia; ivan.ilyin@srcc.msu.ru (I.I.); at@dimonta.com (A.T.); as@dimonta.com (A.S.); dk@dimonta.com (D.K.); vs@dimonta.com (V.S.); 3 Dimonta, Ltd., 117186 Moscow, Russia; 4 Faculty of Physics, Lomonosov Moscow State University, 119992 Moscow, Russia; gantceva.ar16@physics.msu.ru (A.G.); katia_gantseva@mail.ru (E.G.); 5 Russian Children’s Clinical Hospital of the Pirogov Russian National Research Medical University of the Ministry of Healthcare of the Russian Federation, 119571 Moscow, Russia; podoplelovan@yandex.ru; 6 Center for Theoretical Problems of Physicochemical Pharmakology, 119991 Moscow, Russia

**Keywords:** pyrroloquinolinones, anticoagulants, molecular docking, factor Xa, factor XIa

## Abstract

Coagulation factor Xa and factor XIa are proven to be convenient and crucial protein targets for treatment for thrombotic disorders and thereby their inhibitors can serve as effective anticoagulant drugs. In the present work, we focused on the structure–activity relationships of derivatives of pyrrolo[3,2,1-*ij*]quinolin-2(1*H*)-one and an evaluation of their activity against factor Xa and factor XIa. For this, docking-guided synthesis of nine compounds based on pyrrolo[3,2,1-*ij*]quinolin-2(1*H*)-one was carried out. For the synthesis of new hybrid hydropyrrolo[3,2,1-*ij*]quinolin-2(1*H*)-one derivatives, we used convenient structural modification of both the tetrahydro- and dihydroquinoline moiety by varying the substituents at the C^6,8,9^ positions. In vitro testing revealed that four derivatives were able to inhibit both coagulation factors and three compounds were selective factor XIa inhibitors. An IC_50_ value of 3.68 μM for was found for the best factor Xa inhibitor and 2 μM for the best factor XIa inhibitor.

## 1. Introduction

Thrombotic disorders greatly affect global health, being the leading cause of mortality and morbidity [1]. These disorders are mainly caused by abnormal regulation of hemostasis, an important system that controls perfusion and prevents blood loss caused by vascular damage. The main molecular effect of hemostasis activation is the formation of thrombin, an enzyme capable of converting soluble fibrinogen into insoluble strands of fibrin, which, in turn, provide the foundation of the clot. This main enzyme appears as a result of prothrombin conversion catalyzed by prothrombinase complex, in which the activated form of coagulation factor Xa (FXa) plays a key role. FXa activation occurs through either an extrinsic pathway with the participation of tissue factor and factor VII or an intrinsic pathway with the participation of factor VII and factor IXa. Activation of the latter enzyme is caused by factor XIa (FXIa). FXa, FXIa, and thrombin are the most attractive targets in modern pharmacology of antithrombotic medications. Inhibition of thrombin is of less priority because besides hemostasis, this enzyme is involved in other signaling cascades and regulates the clotting process directly instead of through the amplification stage, as FXa and FXIa do [2,3].

Despite the proven efficacy, existing antithrombotic agents, or anticoagulants, possess a number of limitations. Therapy based on warfarin suffers from the need for constant monitoring of the drug plasma concentration, an indirect action mechanism influencing a variety of coagulation factors, and a high risk of bleeding [4]. Because of the high molar mass and poor oral bioavailability, heparin and its fractional form are parenteral medications, which are not suitable for the treatment of chronic thrombotic diseases. Modern non-vitamin K antagonist oral anticoagulants achieve their effect through direct inhibition of key coagulation factors, such as thrombin or FXa. They do not possess the abovementioned issues, but, despite this, their medical use is associated with the risk of life-threatening bleeding events, which require urgent administration of a specific reversal agent. The absence of such a reversal agent is a strong limitation to the widespread application of existing direct FXa inhibitors and direct thrombin inhibitors [5]. Moreover, such serious adverse effects as drug withdrawal syndrome in the case of rivaroxaban and apixaban [6] and an enhanced risk for myocardial infarction in the case of dabigatran [7] complicate their use. There is therefore a need for the search for novel safer FXa inhibitors, probably belonging to other chemical classes. Several recent studies confirmed that FXIa can be an even more convenient target for anticoagulant therapy in terms of efficacy and safety than FXa or thrombin. Therefore, the search for FXIa inhibitors is also of significant priority.

**Figure 1:**
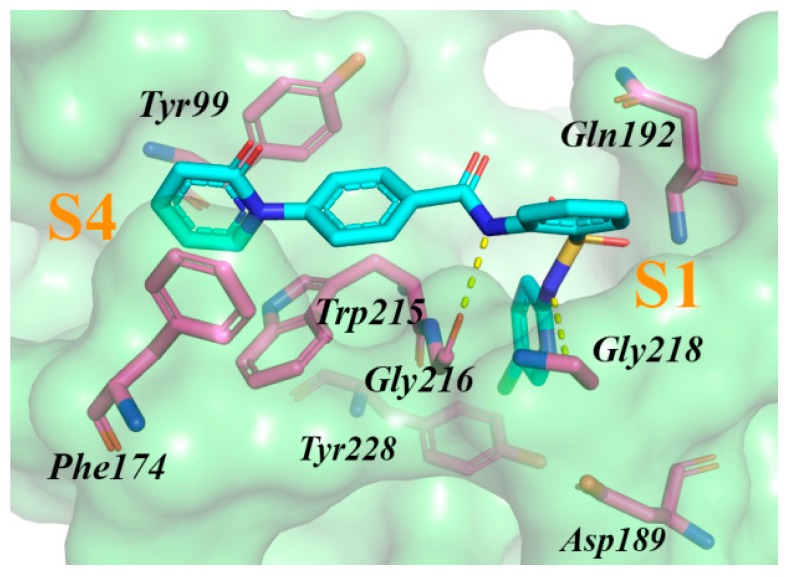
The active site of FXa with a bound nanomolar inhibitor (PDB ID: 3CEN). Hydrogen bonds are shown as yellow dotted lines. Buried residues are labeled by the lower font size.

FXa is a two-chained serine protease causing cleavage of Arg-Thr and then Arg-Ile bonds in prothrombin. The heavy chain contains a trypsin-like protease domain and consists of 303 amino acids. The light one is comprised of 139 amino acids. The first strategies to block FXa were built around using basic amidine-based compounds mainly interacting with the Asp189 of S1 binding pocket. However, owing to the poor bioavailability of high basic compounds, medicinal chemists thereafter focused on low basic and non-basic inhibitors. In such FXa inhibitors, P1 moieties contain aromatic electron-poor systems and interact with Tyr228 through π stacking and Asp189 through anion–π interaction. Examples of these P1 motifs include mono-halogen-substituted benzene, methoxy-substituted benzene, and substituted heterocycles. P4 moieties of most FXa inhibitors contain aromatic groups often coupled with a saturated heterocyclic fragment. There are a variety of scaffolds by which the P1 moiety and P4 moiety can be oriented in the proper manner to bind to the corresponding pockets. The two main features of any scaffold common for effective FXa inhibitors are the ability to form V- or L-shaped molecules and the presence of hydrogen bond donors/acceptors to interact with Gly216 and/or Gly218 [8]. Figure 1 depicts the structure of the active site of FXa and the crucial residues, which known FXa inhibitors interact with.

FXIa represents a disulfide-linked homodimer in which each monomer consists of 607 amino acids. Each subunit consists of a heavy chain and a light chain. The latter contains a trypsin-like catalytic domain similar to other trypsin-like serine proteases [9]. The S1 pocket represents the deep hollow, with Asp189 placed not at the base, as in FXa, but a bit higher in the wall of the pocket. The S1′ pocket is placed opposite to the catalytic triad Asp102, His57, and Ser195, near a disulfide bridge formed by Cys40-Cys58. The S2′ pocket contains the β-chain, which includes the residues Arg39, His40, and Leu41, where polar interactions of inhibitors are often observed, as well as the residues Ile151 and Tyr143 [9].

**Figure 2:**
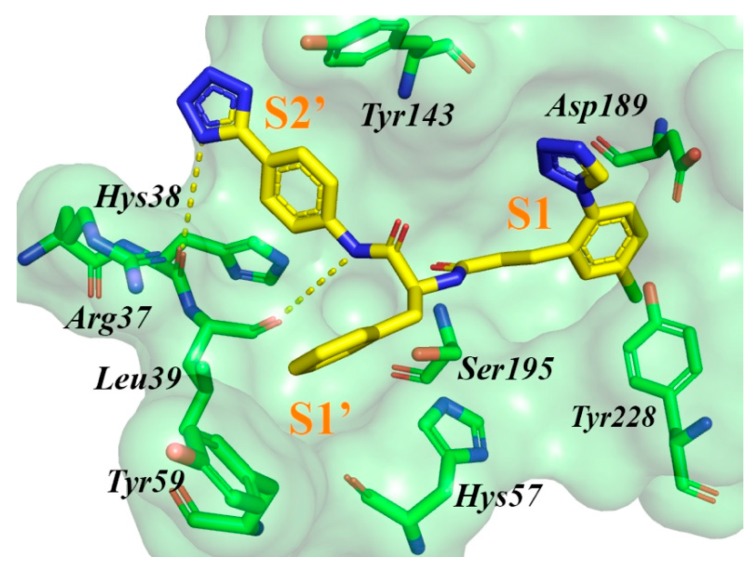
The active site of the FXIa with an inhibitor (PDB ID: 4CRC). Hydrogen bonds are shown as yellow dotted lines.

Known FXIa inhibitors in crystal complexes from PDB occupy all mentioned pockets and exhibit a Y-like shape. The geometry of the active center of factor XIa with its inhibitor is shown in Figure 2. P1 moieties often contain aromatic rings with deactivating substituents. The phenylalanine-like scaffold present in some inhibitors occupies the hydrophobic S1′ pocket and forms a hydrogen bond with Ser195. In the experimental structures, in addition to the neutral terminal fragments of the inhibitors, end-charged moieties are also applied to interact with charged residues, Asp189 in the S1 pocket and Arg37 in the S2′ pocket.

Computational chemistry is immensely useful to reduce the costs and time required for the discovery of new actives. Molecular docking [10,11] is a key tool in computational drug design that is able to predict the geometry of a protein–ligand complex and the binding affinity by relying upon the structure of the target biomolecule [12]. It may be applied for various tasks ranging from docking-based virtual screening for high-throughput sampling chemical space to lead optimization [13]. Despite the proven usefulness and efficacy in an accurate prediction of a bound conformation of the ligand, molecular docking possesses a few limitations among which the relatively high false-positive rate is the main one [14,15]. To solve this issue, some post-docking procedures aimed at a more accurate estimation of binding energy are often applied. One possible method to improve energy estimation and to reduce the false-positive rate is semiempirical postprocessing based on the PM7 method, thus accounting for solvent effects in the frame of the COSMO model, as shown by recent studies.

In the present study, continuing our previous work on the search for compounds with anticoagulant activity, we designed and synthesized derivatives of pyrrolo[3,2,1-*ij*]quinolin-2(1*H*)-one as potential inhibitors of FXa and FXIa. The design of the analogs was mainly based on the molecular hybridization paradigm, implying that the combination of structural features from known active molecules is used for lead optimization [16]. The synthesis of new derivatives of pyrrolo[3,2,1-*ij*]quinolin-2(1*H*)-one was reached by using a novel diversification of substitutes at the C^6,7,8^ positions. To test the activity of the synthesized compounds, amidolytic assays were applied to determine the IC_50_ values for the best inhibitors.

## 2. Results and Discussion

### 2.1. Design of Pyrrolo[3,2,1-Ij]quinolin-2(1H)-One-Based Derivatives

**Figure 3:**
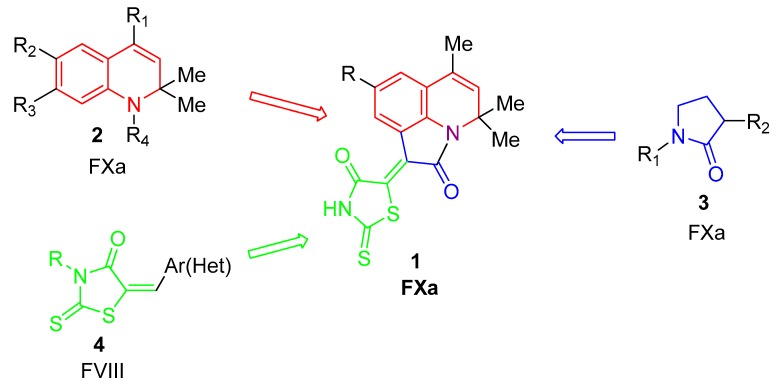
An example of the molecular hybridization strategy. Structure of hybrid FXa inhibitors found in our previous study (**1**) and structural fragments of some known inhibitors of coagulation factors: 1,2-dihydroquinoline (**2**), pyrrolidinone (**3**), and rhodanine (**4**).

In our previous work [17,18], derivatives of pyrrolo[3,2,1-*ij*]quinolin-2(1*H*)-one (PQ, **1**) were found to be perspective FXa inhibitors, with IC_50_ values in the range of 0.7 to 40 μM. These identified inhibitors represent hybrid molecules consisting of dihydroquinoline (**2**) [18], pyrrolidinone (**3**) [19], and rhodanine (**4**) [20] (see Figure 3), which are known to be active moieties of different inhibitors of coagulation factors.

**Figure 4:**
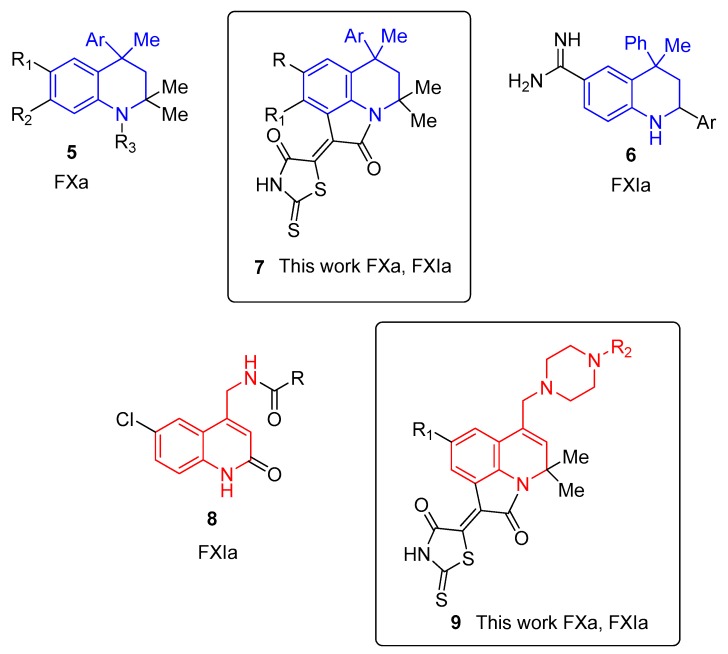
Substituted 1,2,3,4-tetrahydroquinolines (**5,6**)**,** N-acetylaminomethyl-1,2-dihydroquinoline (**8**), and hybrid moieties studied in this work as the basis for FXa and FXIa inhibitors (**7** and **9**–shown in boxes).

1,2,3,4-Tetrahydroquinolines (**5,6**) with phenyl and methyl substituents [18,21] as well as 1,2-dihydroquinolines containing N-acetylaminomethyl groups at C^4^ (**8**) [22] (see Figure 4) are also proven to form a structural basis for compounds with anticoagulant activity. Inhibitory activity against factor FXa was also detected for other 1,2,3,4-tetrahydroquinolines [23]. On the other hand, the introduction of piperazine fragments into the structure of biologically active compounds is considered as one of the most promising directions in medicinal chemistry for the construction of hybrid molecules [24,25,26].

Following the hybridization strategy and combing structural fragments, we thereby designed the focused library of new hybrid PQs, which can be divided into two classes: C^6^ aryl-substituted pyrroloquinolindiones (PQD) and piperazinylmethyl-PQD derivatives (Figure 3 and Figure 4—structures **7** and **9**). The total size of the focused virtual library was 40 compounds, 16 derivatives of aryl-substituted PQD and 24 derivatives of piperazinylmethyl-PQD derivatives. Virtual aryl-substituted PQD-based derivatives mainly differ in the position and substituent type introduced in the aryl group as well as in the substituents in the benzene ring of PQD at C^9,10^. For both places, the aryl fragment and the benzene ring of PQD, a methyl group, halogens, and a methoxy group were used as substituents. The design of virtual piperazinylmethyl-PQD derivatives implied varying substituents at the second nitrogen atom in piperazine as well as at C^9^ of PQD. Monohalogen-substituted aryl groups, methylenebenzodioxole, an acetylethyl group, an acetylpropyl group, and methylenebenzene were applied for an introduction in the piperazine fragment and a methyl group and a fluorine atom were used as C^9^ substituents. We considered only the Z configuration of a double bond between pyrroloquinoline and rhodamine. All derivatives were drawn manually in MarvinSketch and saved in the united sdf-file for further preparation.

**Scheme 1:**
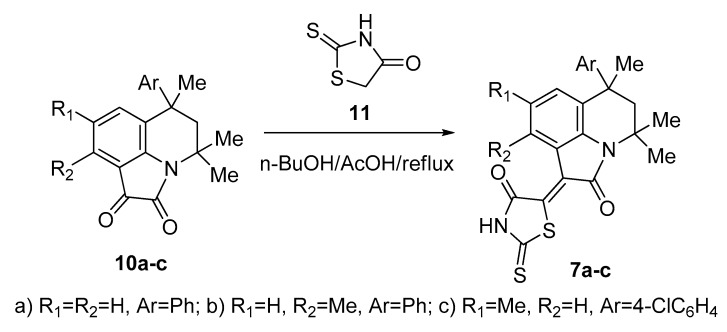
Synthesis of tetrahydro-1H-pyrrolo[3,2,1-ij]quinolin-1-ylidene-2-thioxothiazolidin-4-ones **7a**–**c**.

Derivatives of PQs with a tetrahydroquinoline fragment **7a**–**c** were obtained by the condensation of pyrroloquinolindiones **10** with rhodanine **11** (Scheme 1).

**Scheme 2:**
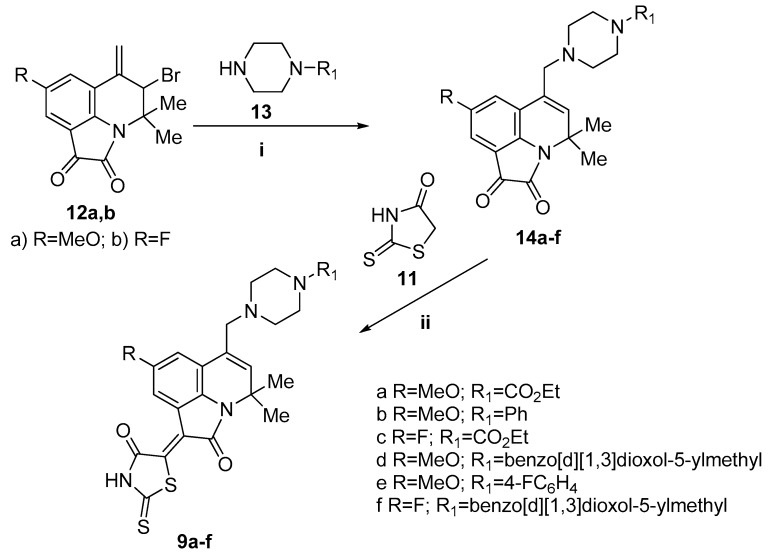
Synthetic route to dihydro-1H-pyrrolo[3,2,1-ij]quinolin-1-ylidene-2-thioxothiazolidin-4-ones **9a**–**f**. (**i**): K_2_CO_3_, KI, MeCN, reflux [27]; (**ii**): AcOH, reflux.

Recently, we showed that the amination of 5-bromo-6-methylene-PQD **12** with monosubstituted piperazines **13** proceeds according to the *tele*-substitution scheme with the formation of 6-piperazinylmethyl-PQD **14** [27]. This unusual conjugated nucleophilic addition-elimination reaction was selected to obtain PQD **14a, c**–**f** (Scheme 2).

Condensation of PQD **14a,c**–**f** with rhodanine **11** in acetic acid led to the desired 6-piperazinylmethyl-2,4-dihydro-1H-pyrrolo[3,2,1-ij]quinolin-1-ylidene-2-thioxothiazolidin-4-ones **9a-f**. In the ^1^H NMR spectra of compounds **9**, the signals of C^9^-H protons were shifted to the low-field region (8.21–8.53 ppm) compared with their position for the initial PQD **14a, c**–**f** (7.25–7.55 ppm). The signals of these protons in tetrahydroquinoline derivatives **7a, c** were also located at 8.51 and 8.74 ppm. The low-field shift is due to the anisotropic effect of the rhodanine carbonyl group. Thus, the condensation of PQD **10,14** with rhodanine **11** led to the formation of compounds **7,9** with Z configuration and formal trans-arrangement of the carbonyl groups.

### 2.2. Results of Virtual Screening and In Vitro Testing

Prepared virtual compounds were docked into both FXa and FXIa with the SOL program. Primary results indicated that among the studied molecules, there are potential dual inhibitors capable of inhibiting both the target protein since the scoring function values are good for both FXa and FXIa. In the case of FXa, the visual inspection of the binding modes helped to reject a few compounds with too long P1-moieties, which did not fit into the S1 pocket. The inspection for poses docked into FXIa resulted in the rejection of compounds that showed the wrong orientation, out of the active site.

For the 25 best potential inhibitors of FXa and for the 19 best FXIa inhibitor candidates, post-docking procedures were applied to calculate the binding energies more accurately. Considering all the criteria together, we selected the abovementioned nine compounds for synthesis. Among them, the ratio between C^6^ benzene-substituted PQDs and C^6^ piperazinylmethyl-substituted PQDs was 3:6. After their synthesis, all nine compounds were checked in an amydolityc assay against both FXa and FXIa. Rivaroxaban, an oral direct Factor Xa inhibitor, was used as the reference substance. The percent inhibition at 30 μM of rivaroxaban for FXa was 94 ± 3.9 and 4 ± 4.6 for FXIa. The results for FXa and FXIa, both calculated and experimental, are listed in Table 1.

Relying upon the results, some structure–activity relationships of PQ-based derivatives were revealed. In the case of the three found FXa inhibitors based on C^6^ benzene-substituted PQDs (**7a**, **7b**, and **7c**), we found that the introduction of a methyl group at C^10^ of PQD is explicitly associated with decreasing activity and this is probably due to the improper orientation of 2-thioxothiazolidine-4-one in the S4 pocket as can be seen from the docking pose (Figure S1). Because of the presence of a methyl group, thioxothiazolidineone is arranged out-of-plane of the scaffold, which hinders **7b** interacting with aromatic residues of the pocket and destabilizes its structure. Among the second group of derivatives, compounds based on mercapto-substituted PQDs, there were no significant actives despite the good calculated properties. The calculated binding enthalpies are shown to describe the observed inactivity better and are associated with the lower false-positive rate. The results for the piperazinylmethyl substituted derivatives of PQD clearly indicate two peculiarities. The first one is that an introduction of a methoxy group at C^9^ can clearly be attributed to increasing activity, which seems to be related to the formation of a hydrogen bond between an oxygen atom of this group and Gly218NH in the docking studies. The second conclusion confirms the known fact that applying halogen-substituted benzene as the P1 moiety for inhibition of FXa is more effective in terms of activity than a simple phenyl group.

From the nine synthesized derivatives of PQ, seven candidates were active against FXIa at 30 μM. Surprisingly, all identified FXa inhibitors were also active against FXIa. We assume that the higher number of actives inhibiting FXIa can be partly explained by the fact that FXIa possesses a larger active site in comparison with the active site of FXa. The inactivity of **7b** from all C^6^ benzene-substituted derivatives of PQD can be possibly explained by the destabilizing effect of a methyl group distorting a plane formed by thioxothiazolidineone and the scaffold as described above. Structural comparison of **9a** and **9c** allows the conclusion that the introduction of a methoxy group at the C^9^ position results in the loss of activity against FXIa. Analysis of the best docking pose of **9a** and **9c** revealed that the presence of a methoxy group at C^9^ may lead to a clash with Lys192, making it impossible for **9a** to reproduce the binding mode of **9c.** As can be seen from Figure S2, a fluorine atom of **9c** lies very close to a hydrogen atom of Lys192 and replacing this fluorine with a bulkier methoxy group should lead to steric hindrance. **9a** is therefore not able to place its positively charged moiety containing piperazine into the negatively charged S1 pocket and form tight contacts with FXIa. This hypothesis does not conflict with the observed activity for **9b**, which also contains a methoxy group at C^9^, since there is a difference in the length of the fragments containing piperazine between **9c** and **9b**, allowing the latter to occupy the S1 pocket without close contact near the methoxy group and Lys192. In general, the observed activity for the rest of the piperazinylmethyl-substituted PQDs is consistent with the overall number of negatively charged residues, which are able to interact with positively charged piperazine.

### 2.3. Determining IC_50_ Values and Mechanism of Inhibition

**Figure 5:**
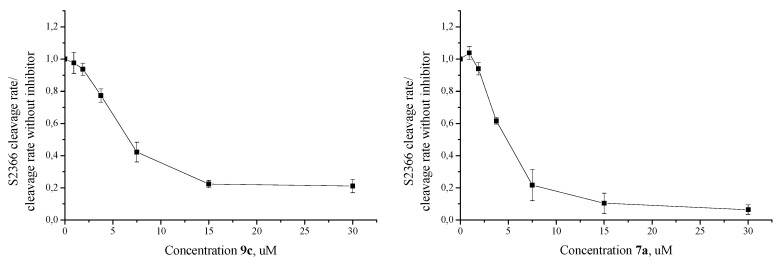
The dependence of inhibition of factor XIa-induced chromogenic substrate hydrolysis on the inhibitor concentration.

For the four best FXIa inhibitors, **7a, 7c,** and **9c**, additional measurements were performed to determine the IC50 values. The kinetics of hydrolysis of the specific substrate S2765 or S2366 by factor Xa and XIa, respectively, in the buffer solution and in the presence of various concentrations of compounds was measured. The results are presented in Figure 5. The values of IC50 are shown in Table 2.

## 3. Material and Methods

### 3.1. Molecular Docking Studies with Semiempirical Postprocessing

All docking simulations were performed in the SOL program. This program applies the rigid rotor approximation and the genetic algorithm for conformational sampling. SOL performs the grid-based docking for which a set of grids is constructed by an auxiliary program, SOLGRID. SOL’s scoring function is physics based and applies the MMFF94 force field for energy estimation for electrostatic and van-der-Waals terms and the generalized Born solvation model to account for solvent effects. The entropy term is assessed as a loss of torsional degrees of freedom. Other features of SOL and a more detailed description can be found in articles [28,29] and its application for the design of inhibitors in [21,22,30,31]. As in popular docking programs with stochastic sampling, docking solutions from SOL undergo cluster analysis to estimate for the given ligand whether the exploration of the conformational space was reliable at selected parameters of the genetic algorithm. The high population of the first cluster, which is formed by the best docking pose, and a low number of clusters are the main clues pointing out the reliability of docking results. Otherwise, repeated docking with increased parameters is a must.

To predict the anticoagulant activity by docking, we retrieved high-quality structures of target proteins from PDB: Structure of FXa from the 3CEN complex and FXIa from 4CRC. Both crystal complexes possess good resolution (<2.0 Å) and no missing residues. Protein structures were manually cleaned from native ligands, water molecules, and salt ions. Their protonation was made in the Aplite program. Extracted native ligands were protonated in Avogadro [32]. Validation of the prepared protein structures was performed by the docking of native ligands and docking of known FXa and FXIa inhibitors. Both docking procedures for native ligands (the FXA ligand into prepared FXa from 3CEN and the OTM ligand into prepared FXIa from 4CRC) were successful, with RMSD values between the best docking pose and the native conformation less than 1.4 Å. To study the known binders, we retrieved 10 crystal complexes of FXa and 10 complexes of FXIa with high active inhibitors. The mean score for the known FXa inhibitors was −6.76 ± 0.44 kcal/mol and the mean score for the known FXIa inhibitors was −5.53 ± 0.56 kcal/mol. These values further served as a score cutoff for the selection of candidates.

A more accurate estimation of the binding affinities was performed by applying semiempirical postprocessing for compounds that scored best. Postprocessing consisted of the following steps. Firstly, we performed local optimization of the protein–ligand complex by using the PM7 method [33] with varying positions of all ligand atoms (taken from the best docking pose from SOL) and fixing protein atoms. To obtain the heat of formation of the optimized complex, single self-consistent field calculation with consideration of the solvent effects in the frame of the COSMO model [34] (1SCF + COSMO) was performed. The heat of formation of the unbound ligand was calculated by a similar way with local optimization of its low energy conformation obtained from OpenBabel [35] at the first step and with subsequent 1SCF + COSMO calculation at the second step. Computing the heat of formation of unbound protein was carried out by direct 1SCF + COSMO calculation with no preliminary optimization. Finally, binding enthalpy was calculated by an equation:

*∆H_bind_* = *∆H_PL_* − (*∆H_P_* + *∆H_L_*)

where *∆H_P_, ∆H_L_*, and *∆H_PL_* are the heats of formation of an unbound protein, an unbound ligand, and their complex, respectively. All calculations related to postprocessing were carried out in the MOPAC program [36]. To perform calculations including thousands of atoms, the MOZYME module [37], which replaces the standard SCF procedure with a localized molecular orbital method, was applied.

### 3.2. Virtual Screening of Focused Library

Designed virtual derivatives of pyrrolo[3,2,1-*ij*]quinolin-2(1*H*)-one (see Section 2.1) were drawn manually by using MarvinSketch and protonated with ChemAxon Protonation module at pH 7.4 [38]. Low-probability protomers were rejected from the protonated 2D library with a simple Python script. OpenBabel was used for generating 3D coordinates. Because of additional protomers, the focused library was enlarged from 40 to 62 molecules.

The prepared library of pyrrolo[3,2,1-*ij*]quinolin-2(1*H*)-ones was subjected to virtual screening with the following approach. To begin with, all compounds were docked into FXa and FXIa with the SOL program at standard parameters: Number of independent runs of 50, population size of 30,000, mating pool size of 70, and number of generations of 1000. Compounds that showed a low population of the first cluster were re-docked with heightened parameters (number of runs of 100, population size of 300,000/3000,000) in order to increase the reliability of the docking simulation. After docking, the binding modes of the top compounds with scoring function values close to the mean score showed by known inhibitors or better were visually inspected to reject compounds exhibiting the wrong occupation of the protein active site. PyMOL [39] was applied for both visual inspection and making figures. Compounds with good binding modes were subjected to semiempirical postprocessing to predict the binding affinity more precisely. Finally, the best virtual candidates were supposed to be synthesized.

### 3.3. Synthesis

#### 3.3.1. Instrumentation

NMR ^1^H and ^13^C spectra were registered on a Bruker DRX−500 (500.13 and 125.76 MHz, respectively) spectrometer (Bruker Corporation, Billerica, MA, USA) in DMSO-*d*_6_, and the internal standard was TMS. Melting points were determined on Stuart SMP 30 (Cole-Palmer, Staffordshire, UK). To control the reagent and products individuality, qualitative analysis of reaction mass was performed by TLC on Merck TLC Silicagel 60 F_254_ chromatographic plate (Merck KGaA, Darmstadt, Germany); eluents: Methanol, chloroform, and their mixtures in various rations. The chromatograms were developed by UV and iodine vapor.

Product purity was monitored by high performance liquid chromatography with high-resolution mass spectrometric electrospray ionization detection (HPLC-HRMS-ESI) in combination with UV detection. The analyzes were performed on an Agilent 1260 Infinity chromatograph (Agilent Technologies, Santa Clara, CA, USA) and Agilent 6230 TOF LC/MS high-resolution time-of-flight mass detector. The ionization block was double electrospray; the signals were recorded in positive polarity; nebulizer N2 20 psig; desiccant gas N2, 6 mL/min, 325 °C; mass detection range is 50–2000 daltons. Capillary voltage 4.0 kV, fragmentator +191 V, skimmer +66 V, OctRF 750 V. Poroshell 120 EC-C18 column (4.6 × 50 mm; 2.7 μm) was used. Gradient elution: acetonitrile/water (0.1% formic acid); flow rate 0.4 mL/min. Software for processing research results-MassHunter Workstation/Data Acquisition V.06.00. 

#### 3.3.2. Chemicals

Starting 4,4,6-trimethyl-6-phenyl-5,6-dihydro-1*H*-pyrrolo[3,2,1-*ij*]quinoline-1,2(4*H*)-dione (**10a**), 4,4,6,9-tetramethyl-6-phenyl-5,6-dihydro-1*H*-pyrrolo[3,2,1-*ij*]quinoline-1,2(4*H*)-dione (**10b**), 6-(4-chlorophenyl)-4,4,6,8-tetramethyl-5,6-dihydro-1*H*-pyrrolo[3,2,1-*ij*]quinoline-1,2(4H)-dione (**10c**) were provided by Alinda Chemical Ltd., Moscow, Russian Federation. 5-Bromo-8-methoxy-4,4-dimethyl-6-methylene-5,6-dihydro-1*H*-pyrrolo[3,2,1-*ij*]quinoline-1,2(4*H*)-dione (**12a**), 5-bromo-8-fluoro-4,4-dimethyl-6-methylene-5,6-dihydro-1*H*-pyrrolo[3,2,1-*ij*]quinoline-1,2(4*H*)-dione (**12b**), 8-methoxy-4,4-dimethyl-6-((4-phenylpiperazine-1-yl)methyl)-1*H*-pyrrolo[3,2,1-*ij*]quinolin-1,2(4*H*)-dione (**14b**) were prepared according to the reported procedure [27]. Other reagents were purchased from commercial suppliers and used as received.

8-R-4,4-dimethyl-6-((4-R_1_-piperazine-1-yl)methyl)-1H-pyrrolo[3,2,1-ij]quinolin-1,2(4H)-diones **14a,c**–**f** were obtained by our previously published procedure [27].

*Ethyl 4-((8-Methoxy-4,4-dimethyl-1,2-dioxo-2,4-dihydro-1H-pyrrolo[3,2,1-ij]quinolin-6-yl)methyl)piperazine-1-carboxylate***14a.** 0.75 g; yield 61 %; m.p. 156–158 ^o^C; ^1^H NMR, δ (ppm): 1.16 (3H, t, *J* = 7.1 Hz, CH_3_CH_2_); 1.60 (6H, s, 2CH_3_); 2.32–2.40 (4H, m, CH_2_N); 3.20–3.42 (m, CH_2_N + H_2_O); 3.62 (3H, s, CH_3_O); 4.01 (2H, q, *J* = 7.1 Hz, CH_3_CH_2_); 5.64 (1H, s, H-5); 6.88 (1H, d, *J* = 2.4 Hz, H-7(9)); 7.25 (1H, d, *J* = 2.4 Hz, H-7(9)). ^13^C NMR, δ (ppm): 15.0, 27.5, 43.9, 52.6, 56.2, 56.3, 59.5, 61.2, 106.7, 115.2, 119.9, 120.0, 124.4, 135.1, 142.5, 155.0, 156.1, 158.1, 183.3. HPLC-HRMS-ESI, *m*/*z* ([M + H]^+^), calcd for C_22_H_27_N_3_O_5_ + H^+^ 414.2025, found 414.2022.

*Ethyl 4-((8-Fluoro-4,4-dimethyl-1,2-dioxo-2,4-dihydro-1H-pyrrolo[3,2,1-ij]quinolin-6-yl)methyl)piperazine-1-carboxylate***14c.** 0.81 g; yield 69%; m.p. 120–122 °C; ^1^H NMR, δ (ppm): 1.17 (3H, t, *J* = 7.1 Hz, CH_3_CH_2_); 1.62 (6H, s, 2CH_3_); 2.28–2.42 (4H, m, CH_2_N); 3.15–3.42 (m, CH_2_N + H_2_O); 4.02 (2H, q, *J* = 7.1 Hz, CH_3_CH_2_); 5.68 (1H, s, H-5); 7.12–7.25 (1H, m, H-7(9)); 7.38–7.55 (1H, m, H-7(9)).^13^C NMR, δ (ppm): 15.0, 27.6, 43.9, 52.6, 56.5, 59.4, 61.2, 109.5, 109.7, 115.7, 118.9, 119.1, 120.4, 120.5, 124.0, 135.7, 144.5, 155.0, 158.1, 160.0, 182.5. HPLC-HRMS-ESI, *m*/*z* ([M + H]^+^), calcd for C_21_H_24_FN_3_O_4_ + H^+^ 402.1825, found 402.1824.

*6-((4-Benzo[d][1,3]dioxol-5-ylmethyl)piperazine-1-yl)methyl)-8-methoxy-4,4-dimethyl-1H-pyrrolo[3,2,1-ij]quinolin-1,2(4H)-dione***14d.** 0.95 g; yield 67 %; m.p. 158–160 °C; ^1^H NMR, δ (ppm): 1.62 (6H, s, 2CH_3_); 2.23–2.50 (4H, m, CH_2_N + DMSO-d_5_); 3.15–3.25 (2H, m, CH_2_N); 3.25–3.48 (m, CH_2_N + H_2_O); 3.72 (3H, s, CH_3_O); 5.59 (1H, s, H-5); 6.69–6.72 (1H, m, CH_arom_); 6.78–6.82 (2H, m, CH_arom_); 6.87 (1H, d, *J* = 2.0 Hz, H-7(9)); 7.24 (1H, d, *J* = 2.0 Hz, H-7(9)). ^13^C NMR, δ (ppm): 27.5, 52.9, 53.0, 56.2, 56.3, 59.7, 62.1, 101.2, 106/6, 108.3, 109.5, 115.1, 120.0, 120.1, 122.4, 124.8, 132.5, 134.7, 142.5, 146.6, 147.6, 156.1, 158.1, 183.3. HPLC-HRMS-ESI, *m*/*z* ([M + H]^+^), calcd for C_27_H_29_N_3_O_5_ + H^+^ 476.2182, found 476.2185.

*6-((4-(4-Fluoropheny)lpiperazine-1-yl)methyl)-8-methoxy-4,4-dimethyl-1H-pyrrolo[3,2,1-ij]quinolin-1,2(4H)-dione***14e.** 0.79 g; yield 62 %; m.p. 180–182 °C; ^1^H NMR, δ (ppm): 1.62 (6H, s, 2CH_3_); 2.52–2.58 (4H, m, CH_2_N); 3.02–3.08 (4H, m, CH_2_N); 3.27–3.33 (m, CH_2_N + H_2_O); 3.72 (3H, s, CH_3_O); 5.67 (1H, s, H-5); 5.96 (2H, s, OCH_2_O); 6.98 (1H, d, *J* = 2.3 Hz, H-7(9)); 6.90–6.94 (2H, m, CH_arom_); 6.99–7.04 (2H, m, CH_arom_); 7.30 (1H, d, *J* = 2.3 Hz, H-7(9)). ^13^C NMR, δ (ppm): 27.6, 49.6, 52.8, 56.2, 56.3, 59.6, 106.7, 115.2, 115.6, 115.8, 117.6, 116.7, 120.1, 124.7, 134.9, 142.5, 148.4, 155.5, 153.1, 157.4, 158.1, 183.3. HPLC-HRMS-ESI, *m*/*z* ([M + H]^+^), calcd for C_25_H_26_FN_3_O_3_ + H^+^ 436.2032, found 436.2034.

*6-((4-Benzo[d][1,3]dioxol-5-ylmethyl)piperazine-1-yl)methyl)-8-fluoro-4,4-dimethyl--1H-pyrrolo[3,2,1-ij]quinolin-1,2(4H)-dione***14f.** 0.91 g; yield 66 %; m.p. 160–162 °C; ^1^H NMR, δ (ppm): 1.60 (6H, s, 2CH_3_); 2.25–2.46 (6H, m, CH_2_N); 3.20 (2H, s, CH_2_N); 3.22–3.35 (m, CH_2_N + H_2_O); 5.63 (1H, s, H-5); 5.96 (2H, s, OCH_2_O); 6.69–6.72 (1H, m, CH_arom_); 6.79–6.82 (2H, m, CH_arom_); 7.18 (1H, dd, *J* = 7.2 Hz (HF), *J* = 2.3 Hz, H-7(9)); 7.84 (1H, dd, *J* = 10.3 Hz (HF), *J* = 2.3 Hz, H-7(9)). ^13^C NMR, δ (ppm): 27.6, 52.9, 53.0, 56.5, 59.5, 62.1, 101.2, 108.3, 109.5, 109.6, 115.6, 115.7, 118.9, 119.1, 120.5, 122.3, 124.3, 132.5, 135.3, 144.5, 146.6, 147.6, 158.0, 158.1, 160.0, 182.5 HPLC-HRMS-ESI, *m*/*z* ([M + H]^+^), calcd for C_26_H_26_FN_3_O_4_ + H^+^ 464.1981, found 464.1984.

##### General procedure for the synthesis of (*2,4,5,6-tetrahydro-1H-pyrrolo[3,2,1-ij]quinolin-1-ylidene)-2-thioxothiazolidin-4-ones* **7a**–**c**

A mixture of the corresponding pyrroloquinolinedione (**10a**–**c**, 1.5 mmol) and rhodanine (**11**, 1.5 mmol) in n-BuOH (15 mL) containing 1–2 drops of acetic acid was refluxed for 7–10 h and cooled. The resulting precipitate was filtered, washed with i-PrOH and dried.

*(Z)-2-Thioxo-5-(4,4,6-trimethyl-2-oxo-6-phenyl-2,4,5,6-tetrahydro-1H-pyrrolo[3,2,1-ij]quinolin-1-ylidene)thiazolidin-4-one***7a.** 0.38 g; yield 60 %; m.p. 269–271 °C; ^1^H NMR, δ (ppm): 0.72 (3H, s, C^6^-CH_3_); 1.63 (3H, s, C^4^-CH_3_); 1.71 (3H, s, C^4^-CH_3_); 2.15 (1H, d, *J* = 14.3 Hz, C^5^-H); 2.52 (1H, d, *J* = 14.3 Hz, C^5^-H); 7.05–7.08 (2H, m, CH_arom_); 7.15–7.27 (4H, m, CH_arom_); 7.47 (1H, dd, *J* = 7.8 Hz, *J* = 0.5 Hz, H-7); 8.74 (1H, dd, *J* = 7.8 Hz, *J* = 0.5 Hz, H-9); 13.95 (1H, br. s, NH). ^13^C NMR, δ (ppm): 24.9, 28.0, 30.5, 39.6, 50.8, 54.2, 118.5, 122.5 124.6, 126.3, 126.3, 126.6, 128.2, 131.2, 134.3, 141.2, 147.9, 165.5, 169.4, 199.9. HPLC-HRMS-ESI, *m*/*z* ([M + H]^+^), calcd for C_23_H_20_N_2_O_2_S_2_ + H^+^ 421.1040, found 421.1042.

*(Z)-5-(4,4,6,9-Tetramethyl-2-oxo-6-phenyl-2,4,5,6-tetrahydro-1H-pyrrolo[3,2,1-ij]quinolin-1-ylidene)-2-thioxothiazolidin-4-one***7b.** 0.31 g; yield 48 %; m.p. 246–248 °C; ^1^H NMR, δ (ppm): 0.73 (3H, s, C^6^-CH_3_); 1.60 (3H, s, C^4^-CH_3_); 1.68 (3H, s, C^4^-CH_3_); 2.12 (1H, d, *J* = 14.3 Hz, C^5^-H); 2.28 (3H, s, C^9^-CH_3_); 2.46–2.52 (m, C^5^-H + DMSO-d_5_); 7.02 (1H, d, *J* = 8.1 Hz, H-7(8)); 7.08 (2H, d, *J* = 7.8 Hz, CH_arom_); 7.13–7.17 (1H, m, CH_arom_); 7.22–7.26 (2H, m, CH_arom_); 7.36 (1H, d, *J* = 8.1 Hz, CH_arom_); 13.95 (1H, br. s, NH). ^13^C NMR, δ (ppm): 23.8, 25.3, 26.0, 28.3, 31.1, 51.4, 54.5, 119.1, 123,6, 125.9, 126.2, 127.1, 128.6, 131.1, 134.3, 138.6, 141.6, 148.6, 167.1, 168.3, 199.3. HPLC-HRMS-ESI, *m*/*z* ([M + H]^+^), calcd for C_24_H_22_N_2_O_2_S_2_ + H^+^ 435.1197, found 435.1194.

*(Z)-5-(6-(4-Chlorophenyl)-4,4,6,8-tetramethyl-2-oxo-2,4,5,6-tetrahydro-1H-pyrrolo[3,2,1-ij]quinolin-1-ylidene)-2-thioxothiazolidin-4-one***7c.** 0.39 g; yield 56 %; m.p. 288–290 °C; ^1^H NMR, δ (ppm): 0.72 (3H, s, C^6^-CH_3_); 1.60 (3H, s, C^4^-CH_3_); 1.66 (3H, s, C^4^-CH_3_); 2.10 (1H, d, *J* = 14.3 Hz, C^5^-H); 2.32 (3H, s, C^8^-CH_3_); 2.46–2.50 (m, C^5^-H + DMSO-d_5_); 7.08 (2H, d, *J* = 8.3 Hz, CH_arom_); 7.20–7.31 (3H, m, CH_arom_); 8.51 (1H, s, H-9); 13.94 (1H, br. s, NH).. ^13^C NMR, δ (ppm): 21.7, 25.5, 28.3, 30.7, 51.2, 54.4, 119.0, 125.2, 125.9, 127.4, 128.6, 129.0, 131.3, 131.7, 131.9, 139.4, 147.5, 165.8, 168.8, 200.3. HPLC-HRMS-ESI, *m*/*z* ([M + H]^+^), calcd for C_24_H_21_ClN_2_O_2_S_2_ + H^+^ 469.0807, found 469.0808.

##### General procedure for the synthesis of *5-(8-R-4,4-Dimethyl-2-oxo-6-((4-R_1_-piperazin-1-yl)methyl)-2,4-dihydro-1H-pyrrolo[3,2,1-ij]quinolin-1-ylidene)-2-thioxothiazolidin-4-ones* **9a**–**f**

A mixture of the corresponding pyrroloquinolinedione (**14a**–**f**, 1.2 mmol) and rhodanine (11, 1.2 mmol) in acetic acid (15 mL) was refluxed for 5–10 h and cooled. The resulting precipitate was filtered off, washed with cold i-PrOH and recrystallized from i-PrOH/acetic acid.

*(Z)-Ethyl 4-((8-methoxy-4,4-dimethyl-2-oxo-1-(4-oxo-2-thioxothiazolidin-5-ylidene)-2,4-dihydro-1H-pyrrolo[3,2,1-ij]quinolin-6-yl)methyl)piperazine-1-carboxylate***9a.** 0.35 g; yield 55 %; m.p. 264–266 °C; ^1^H NMR, δ (ppm): 1.18 (3H, t, *J* = 7.1 Hz, CH_3_CH_2_); 1.64 (6H, s, 2CH_3_); 2.49–2.54 (m, CH_2_N + DMSO-d_5_); 3.20–3.45 (m, CH_2_N + H_2_O); 3.77 (3H, s, CH_3_O); 4.03 (2H, q, *J* = 7.1 Hz, CH_3_CH_2_); 5.67 (1H, s, H-5); 7.14 (1H, d, *J* = 2.4 Hz, H-7); 8.21 (1H, d, *J* = 2.4 Hz, H-9); 13.50 (1H, br. s, NH). ^13^C NMR, δ (ppm): 14.4, 26.8, 48.0, 51.9, 55.4, 56.4, 58.2, 60.7, 111.5, 113.3, 116.9, 117.7, 123.4, 123.8, 134.3, 134.6, 135.8, 154.4, 154.9, 166.2, 170.8, 200.5. HPLC-HRMS-ESI, *m*/*z* ([M + H]^+^), calcd for C_25_H_28_N_4_O_5_S_2_ + H^+^ 529.1575, found 529.1580.

*(Z)-5-(8-Methoxy-4,4-dimethyl-2-oxo-6-((4-phenylpiperazin-1-yl)methyl)-2,4-dihydro-1H-pyrrolo[3,2,1-ij]quinolin-1-ylidene)-2-thioxothiazolidin-4-one***9b.** 0.41g; yield 64%; m.p. 274–276 °C; ^1^H NMR, δ (ppm) (*low solubility in DMSO*): 1.67 (6H, s, 2CH_3_); 2.65–2.85 (4H, m, CH_2_N); 3.10–3.45 (m, CH_2_N + H_2_O); 3.77 (3H, s, CH_3_O);5.73 (1H, s, H-5); 6.70–7.00 (3H, m, CH_arom_); 7.15–7.30 (3H, m, CH_arom_); 8.25 (1H, s, H-9); 13.25 (1H, br. s, NH). ^13^C NMR, δ (ppm): 27.0, 41.3, 48.2, 52.2, 55.9, 56.4, 58.5, 112.4, 113.6, 115.4, 117.3, 118.0, 118.8, 124.5, 128.8, 134.9, 150.9, 155.3, 166.5, 171.0, 200.4. HPLC-HRMS-ESI, *m*/*z* ([M + H]^+^), calcd for C_28_H_28_N_4_O_3_S_2_ + H^+^ 533.1677, found 533.1674.

*(Z)-Ethyl 4-((8-Fluoro-4,4-dimethyl-2-oxo-1-(4-oxo-2-thioxothiazolidin-5-ylidene)-2,4-dihydro-1H-pyrrolo[3,2,1-ij]quinolin-6-yl)methyl)piperazine-1-carboxylate***9c.** 0.36 g; yield 58 %; m.p. 167–169 °C; ^1^H NMR, δ (ppm): 1.18 (3H, t, *J* = 7.1 Hz, CH_3_CH_2_); 1.66 (6H, s, 2CH_3_); 2.52–2.56 (4H, m, CH_2_N); 3.20–3.40 (m, CH_2_N + H_2_O); 4.04 (2H, q, *J* = 7.1 Hz, CH_3_CH_2_); 5.74 (1H, s, H-5); 7.35 (1H, dd, *J* = 10.0 Hz (HF), *J* = 2.4 Hz, H-7); 8.30 (1H, dd, *J* = 10.0 Hz (HF), *J* = 2.4 Hz, H-9); 13.20 (1H, br. s, NH). ^13^C NMR, δ (ppm): 14.5, 26.9, 42.8, 51.9, 56.7, 58.0, 60.8, 112.6, 112.9, 113.0, 113.2, 117.0, 118.1, 118.2, 121.8, 123.1, 135.5, 136.7, 138.1, 154.5, 157.0, 158.9, 166.3, 171.7, 200.7. HPLC-HRMS-ESI, *m*/*z* ([M + H]^+^), calcd for C_24_H_25_FN_4_O_4_S_2_ + H^+^ 517.1375, found 517.1374.

*(Z)-5-(6-((4-(Benzo[d][1,3]dioxol-5-ylmethyl)piperazin-1-yl)methyl)-8-methoxy-4,4-dimethyl-2-oxo-2,4-dihydro-1H-pyrrolo[3,2,1-ij]quinolin-1-ylidene)-2-thioxothiazolidin-4-one***9d.** 0.43 g; yield 61 %; m.p. 165–167 °C; ^1^H NMR, δ (ppm): 1.63 (6H, s, 2CH_3_); 2.75–3.05 (4H, m, CH_2_N); 3.25–3.45 (m, CH_2_N + H_2_O); 3.85–4.05 (2H, m, CH_2_N); 5.61 (1H, s, H-5); 6.03 (2H, s, OCH_2_O); 6.89–6.90 (1H, m, CH_arom_); 6.94–6.96 (1H, m, CH_arom_); 6.97–6.99 (1H, m, CH_arom_); 7.02 (1H, d, *J* = 2.4 Hz, H-7); 8.43 (1H, d, *J* = 2.4 Hz, H-9); NH not detected. ^13^C NMR, δ (ppm): 26.9, 50.1, 51.1, 55.4, 56.1, 57.9, 59.5, 101.1, 108.1, 110.3, 111.6, 112.0, 117.1, 117.7, 119.9, 124.2, 133.6, 133.8, 147.3, 147.4, 154.7, 166.8, 205.0. HPLC-HRMS-ESI, *m*/*z* ([M + H]^+^), calcd for C_30_H_30_N_4_O_5_S_2_ + H^+^ 591.1732, found 591.1731.

*(Z)-5-(6-((4-(4-Fluorophenyl)piperazin-1-yl)methyl)-8-methoxy-4,4-dimethyl-2-oxo-2,4-dihydro-1H-pyrrolo[3,2,1-ij]quinolin-1-ylidene)-2-thioxothiazolidin-4-one***9e.** 0.36 g; yield 54 %; m.p. 263–265 °C; ^1^H NMR, δ (ppm): 1.66 (6H, s, 2CH_3_); 2.60–2.80 (4H, m, CH_2_N); 3.10–3.20 (2H, m, CH_2_N); 3.23–3.42 (m, CH_2_N + H_2_O); 3.47–3.55 (2H, m, CH_2_N); 5.73 (1H, s, H-5); 6.94–6.98 (2H, m, CH_arom_); 7.02–7.07 (2H, m, CH_arom_); 7.16 (1H, d, *J* = 2.4 Hz, H-7); 8.22 (1H, d, *J* = 2.4 Hz, H-9); 12.85 (1H, br. s, NH). ^13^C NMR, δ (ppm): 26.9, 48.4, 52.0, 55.3, 56.4, 57.8, 111.6, 112.8, 115.1, 115.3, 117.0, 117.1, 117.2, 117.5, 122.8, 123.4, 134.3, 135.0, 136.9, 147.4, 154.9, 155.1, 157.0, 166.3, 172.2, 201.5. HPLC-HRMS-ESI, *m*/*z* ([M + H]^+^), calcd for C_28_H_27_FN_4_O_3_S_2_ + H^+^ 551.1583, found 551.1585.

*(Z)-5-(6-((4-(Benzo[d][1,3]dioxol-5-ylmethyl)piperazin-1-yl)methyl)-8-fluoro-4,4-dimethyl-2-oxo-2,4-dihydro-1H-pyrrolo[3,2,1-ij]quinolin-1-ylidene)-2-thioxothiazolidin-4-one***9f.** 0.42 g; yield 61 %; m.p. 276–278 °C; ^1^H NMR, δ (ppm): 1.65 (6H, s, 2CH_3_); 2.85–3.05 (4H, m, CH_2_N); 3.20–3.40 (m, CH_2_N + H_2_O); 3.92–4.05 (2H, m, CH_2_N); 5.66 (1H, s, H-5); 6.04 (2H, s, OCH_2_O); 6.90–7.00 (3H, m, CH_arom_); 7.24 (1H, dd, *J* = 10.0 Hz (HF), *J* = 2.6 Hz, H-7); 8.53 (1H, dd, *J* = 10.1 Hz (HF), *J* = 2.6 Hz, H-9); NH not detected. ^13^C NMR, δ (ppm) (*low solubility in DMSO*): 27.2, 50.6, 51.6, 56.6, 58.1, 60.3, 101.2, 108.2, 110.2, 112.7, 112.9, 118.1, 123.8, 124.3, 134.6, 147.5. HPLC-HRMS-ESI, *m*/*z* ([M + H]^+^), calcd for C_29_H_27_FN_4_O_4_S_2_ + H^+^ 579.1532, found 579.1529.

### 3.4. In Vitro Assays

The kinetics of factor Xa and XIa inhibition was determined from the hydrolysis reaction of a specific substrate by the enzyme in the presence of the tested substances. Chromogenic substrate is a peptide that reacts with proteolytic enzymes under the formation of color. Attached to the peptide part of the chromogenic substrate is a chemical group of p-nitroaniline (pNa), which when released after the enzyme cleavage, gives rise to color. The rate of pNa formation, i.e., the increase in absorbance per second, is measured photometrically at 405 nm.

The hydrolysis of the chromogenic peptide substrate by the proteolytic enzyme follows in general the Michaelis–Menten kinetics. This means that if the substrate is present at a sufficiently high concentration or if a comparatively small fraction of the substrate is hydrolyzed, the rate of product (color) formation is proportional to the activity of the enzyme.

The chromogenic substrates S2765 (Chromogenix-Instrumentation Laboratory Company, Bedford, MA, USA) for factor Xa and S2366 (Chromogenix-Instrumentation Laboratory Company, Bedford, MA, USA) for factor Xa were used for registering the accumulation of p-nitroaniline as a colored product by a spectrophotometer THERMOmax Microplate Reader (Molecular Devices Corporation, San Jose, CA, USA).

Firstly, all compounds were diluted in DMSO in a concentration of 1.5 mM. Then, compound solutions in DMSO were diluted 25-fold in water buffer (20mM HEPES, 140mM NaCl and 0.1% polyethylene glycol 6000Da, pH 7.4), mixed with factor Xa or XIa, warmed up for 5 min at 37 C, and added to plate well containing S2765 or S2366, respectively. The final solution in wells contained 2.5 nM of factor Xa or 0.8 nM of factor XIa, 100 uM of S2765 or S2366, 30 uM of potencial inhibitor, and 2% of DMSO.

The hydrolysis rate was monitored spectrophotometrically at 405 nm (absorption maximum of the reaction product p-nitroaniline). The results of these measurements were implied to measure the kinetics of factor Xa inhibition by various inhibitors.

The initial rate was determined as the slope of the linear part of the kinetic curve over the first 3 min of measurement. The inhibitory effect was expressed as the percentage of reduction of the initial hydrolysis rate. The reaction rate in the absence of any inhibitor was taken as 100%. Each result is the mean of three parallel determinations ± SE. For the most promising compounds, the titration was performed and IC50 was determined.

The processing of results was performed using the GraphPad Prism (GraphPad, San Diego, CA, USA) and OriginPro 8 (OriginLab Corporation, Suite, Northampton, MA, USA).

## 4. Conclusions

Herein, we developed a convenient path for the synthesis of new hybrid di- and tetrahydropyrrolo[3,2,1-*ij*]quinolin-1-ylidene-2-thioxothiazolidin-4-ones’ condensation of polyfunctional pyrrolo[3,2,1-*ij*]quinolindiones with rhodanine. The structure of the obtained compounds determined the wide possibilities of its directed optimization by varying the substituents both in the pyrroloquinoline and in the rhodamine fragments. Moreover, a few synthesized hybrid molecules were proven to be active against factor Xa and factor XIa.

## Figures and Tables

**Table 1:** Predicted and observed inhibition values for the nine synthesized derivatives of PQ against FXa and FXIa. The larger the value of percent inhibition, the more active the ligand.

#	Compound	Structure	Factor Xa			Factor XIa		
			Scoring Kunction in SOL, kcal/mol	Binding Enthalpy by PM7, kcal/mol	Percent Inhibition at 30 μM	Scoring Function in SOL, kcal/mol	Binding Enthalpy by PM7, kcal/mol	Percent Inhibition at 30 μM
1	**7a**	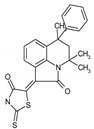	−6.22	−4.6	74 ± 8.8	−4.40	−26.20	100 ± 0.3
2	**7b**	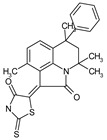	−5.77	−4.9	33 ± 1.6	−4.61	−17.74	7 ± 3.1
3	**7c**	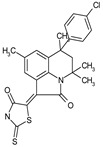	−6.12	−4.52	47 ± 1.7	−5.64	−33.20	87 ± 1.4
4	**9a**	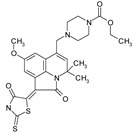	−5.70	−42.69	0	−4.12	−20.07	0
5	**9b**	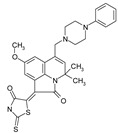	−5.74	−43.62	9 ± 4	−5.67	−32.29	54 ± 1.6
6	**9c**	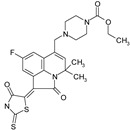	−5.53	−78.71	20 ± 7	−4.82	−26.69	96 ± 2.2
7	**9d**	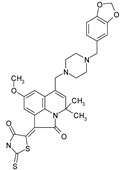	−6.17	−50.13	56 ± 5	−5.35	−20.05	49 ± 0.6
8	**9e**	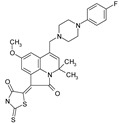	−5.89	−43.47	69 ± 1	−6.57	−22.50	71 ± 2.1
9	**9f**	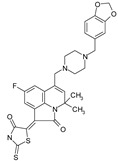	−6.21	−45.44	37 ± 2	−5.84	−25.71	64 ± 2

**Table 2:** Results of the experimental measurements of the IC_50_ values of factor XIa inhibition for various compounds.

Compound	Inhibition of Hydrolysis Rate Specific to the Factor XIa Substrate in the Buffer System IC_50_, µM
**7a**	4.30 ± 0.346
**7c**	9.42 ± 0.986
**9c**	5.31 ± 0.355

## Supplementary Materials

The following are available online, Figure S1, Figure S2, the NMR spectra, data of HPLC-MS-ESI analysis of PQ **7, 9, 14**.
