# Supplementary material for: Synthesis, Docking, and In Vitro Anticoagulant Activity Assay of Hybrid Derivatives of Pyrrolo[3,2,1-ij]Quinolin-2(1H)-one as New Inhibitors of Factor Xa and Factor XIa
Source: Molecules. 2020 Apr 19;25(8):1889. doi: 10.3390/molecules25081889 (PMC7222003; doi:10.3390/molecules25081889)
Supplement: Supplementary file 1 [file molecules-25-01889-s001.pdf]

Supplementary Materials:

Synthesis, docking and in vitro anticoagulant activity assay of hybrid derivatives of pyrrolo[3,2,1-*ij*]quinolin-2(1*H*)-one as new inhibitors of factor Xa and factor Xia

Article

Synthesis, Docking, and In Vitro Anticoagulant Activity Assay of Hybrid Derivatives of Pyrrolo[3,2,1-*ij*]Quinolin-2(1*H*)-one as New Inhibitors of Factor Xa and Factor XIa

Nadezhda Novichikhina [1], Ivan Ilin [2,3], Anna Tashchilova [2,3], Alexey Sulimov [2,3], Danil Kutov [2,3], Irina Ledenyova [1], Mikhail Krysin [1,*], Khidmet Shikhaliev [1], Anna Gantseva [4], Ekaterina Gantseva [4], Nadezhda Podoplelova [5,6] and Vladimir Sulimov [2,3]

[1] Department of Organic Chemistry, Faculty of Chemistry, Voronezh State University, 1 Universitetskaya sq., Voronezh 394018, Russia; podaneva_nadya@mail.ru (N.N.); irairachem@yandex.ru (I.L.); chocd261@chem.vsu.ru (K.S.)

[2] Research Computing Center, Lomonosov Moscow State University, Moscow 119992, Russia; ivan.ilyin@srcc.msu.ru (I.I.); at@dimonta.com (A.T.); as@dimonta.com (A.S.); dk@dimonta.com (D.K.); vs@dimonta.com (V.S.)

[3] Dimonta, Ltd., Moscow 117186, Russia

[4] Faculty of Physics, Lomonosov Moscow State University, Moscow 119992, Russia; gantceva.ar16@physics.msu.ru (A.G.); katia_gantseva@mail.ru (E.G.)

[5] Russian Children's Clinical Hospital of the Pirogov Russian National Research Medical University of the Ministry of Healthcare of the Russian Federation, Moscow 119571, Russia; podoplelovan@yandex.ru

[6] Center for Theoretical Problems of Physicochemical Pharmakology, Moscow 119991, Russia

* Correspondence: kaf261@rambler.ru; Tel.: +7–903–651–7482

Academic Editor: Athina Geronikaki

Received: 29 March 2020; Accepted: 17 April 2020; Published: date

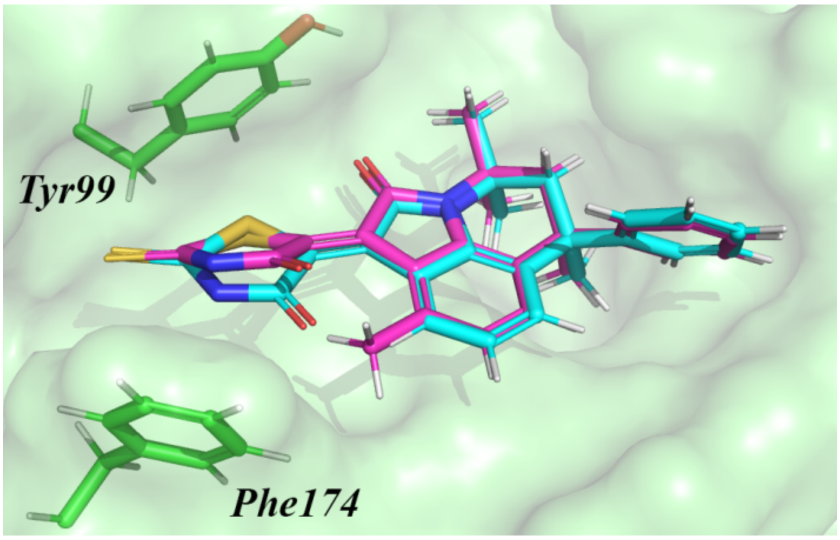

Figure S1. The docking pose of **7a** and **7b** in an active site of FXa. A methyl group in PQD of **7b** (purple carbon atoms) distorts the plane formed by thioxothiazolidineone and the scaffold which is observed for **7a** (blue carbon atoms). The distorted position of thioxothiazolidineone hinders favorable pi-pi interactions with Tyt99/Phe174.

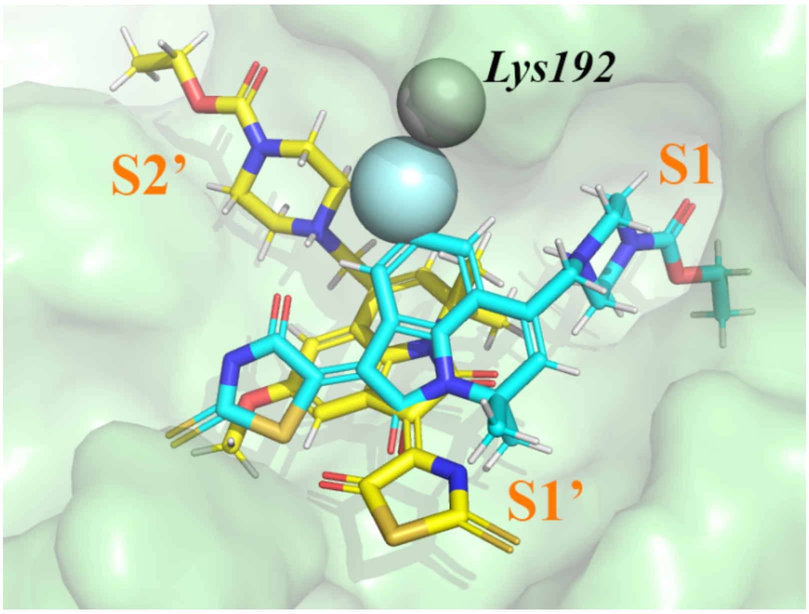

Figure S2. The docking pose of **9a** (yellow carbon atoms) and **9c** (blue carbon atoms) in an active site of FXIa. The small distance between the scaffold of presented ligands near C^9 position and Lys192 does not allow introducing bulky substituents at C^9. Spheres based on van der Waals radii show this small distance between a fluorine atom of (a blue sphere) **9c** and a hydrogen atom of Lys192 (a gray sphere). Because of steric hindrance, **9a** cannot reproduce the binding mode of **9c**.

^{1}H, ^{13}C NMR spectra and data of HPLC-MS-ESI analysis of PQ 7

(*Z*)-2-thioxo-5-(4,4,6-trimethyl-2-oxo-6-phenyl-2,4,5,6-tetrahydro-1*H*-pyrrolo[3,2,1-*ij*]quinolin-1-ylidene)thiazolidin-4-one 7a

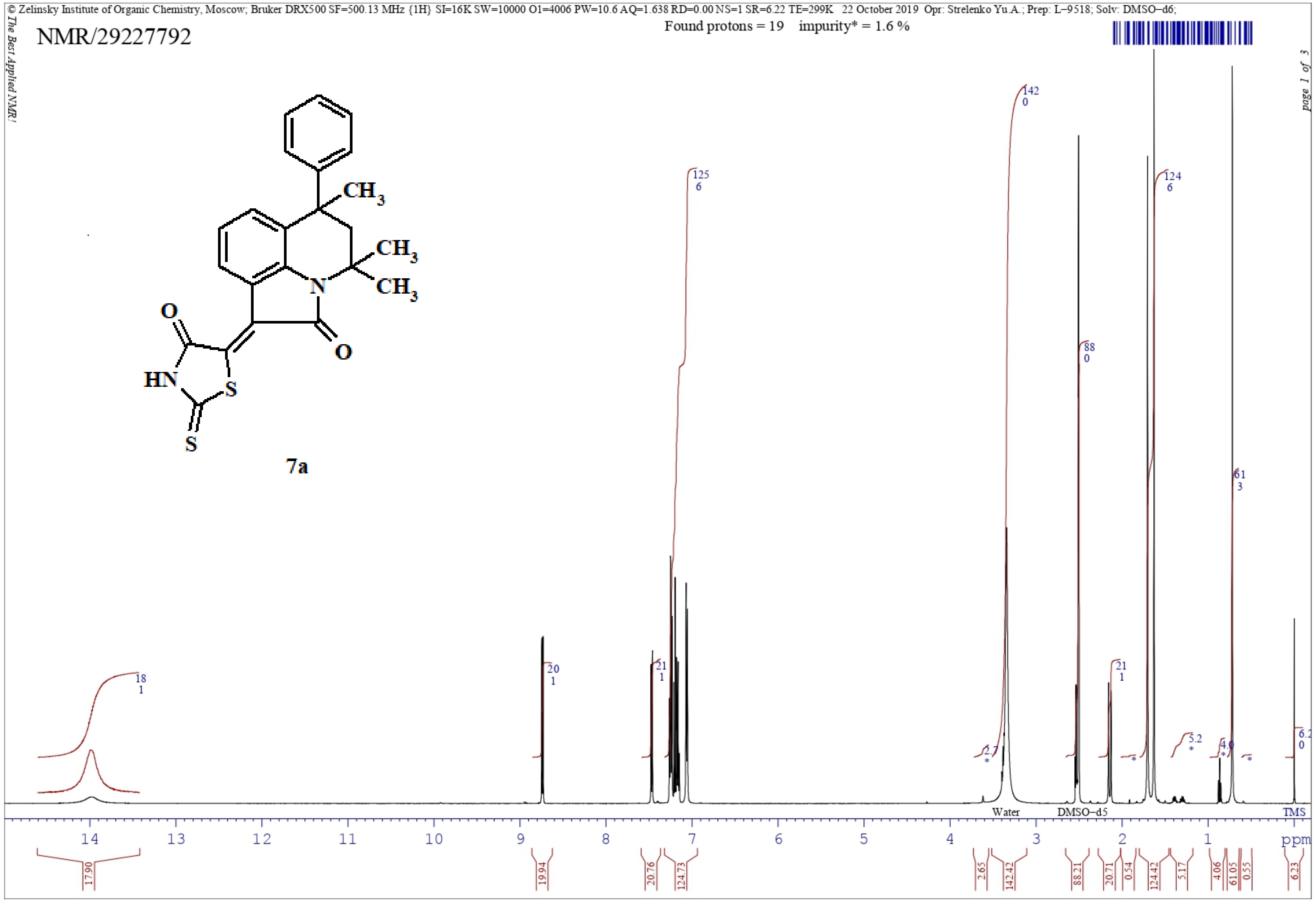

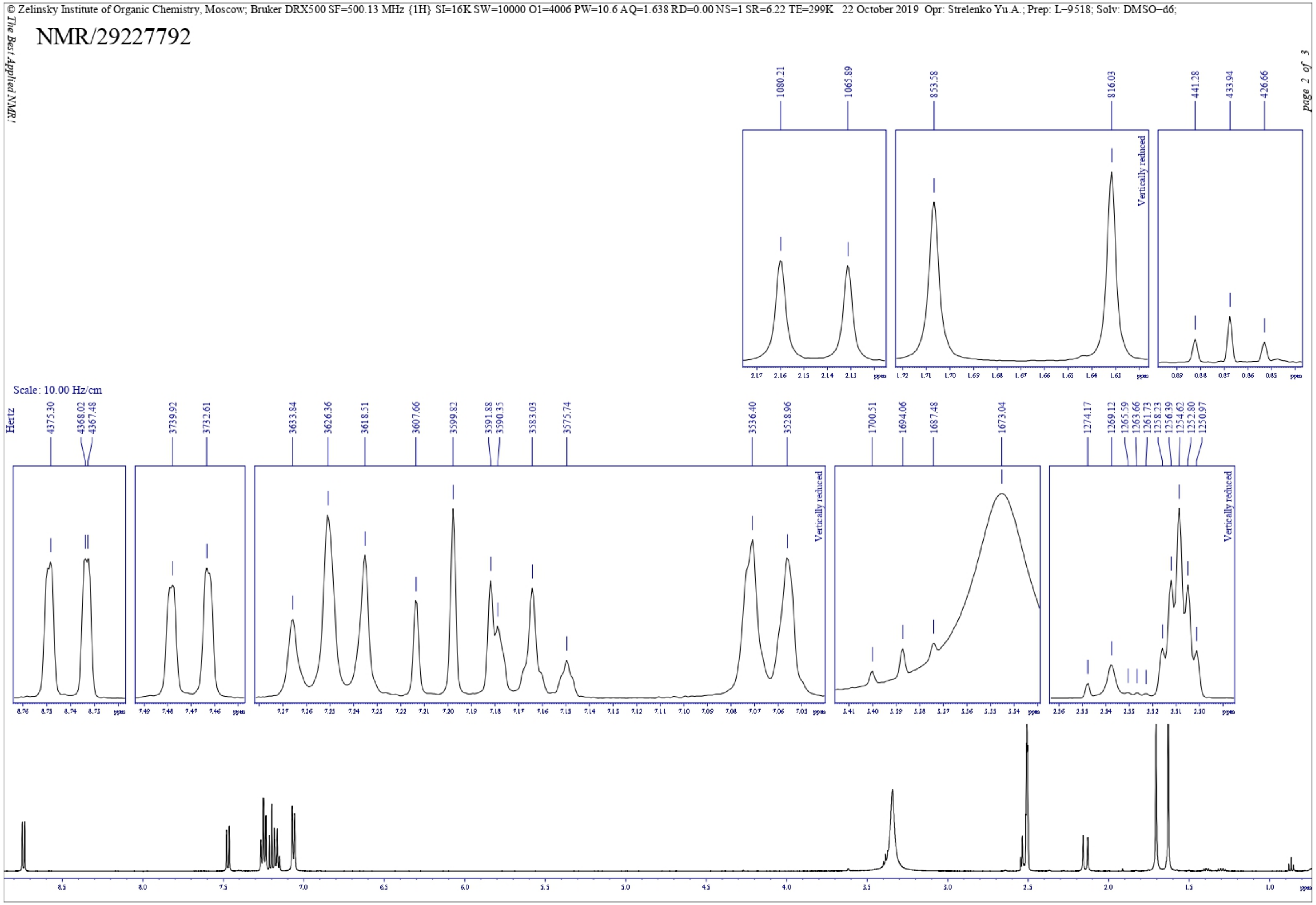
© Zelinsky Institute of Organic Chemistry, Moscow; Bruker DRX500 SF=500.13 MHz {1H} SI=16K SW=10000 O1=4006 PW=10.6 AQ=1.638 RD=0.00 NS=1 SR=6.22 TE=299K 22 October 2019 Opr: Strelenko Yu.A.; Prep: L−9518; Solv: DMSO−d6;
NMR/29227792
The Best Applied NMR!
page 2 of 3
1080.21
1065.89
853.58
816.03
Vertically reduced
441.28
433.94
426.66
Scale: 10.00 Hz/cm
Hertz
4375.30
4368.02
4367.48
3739.92
3732.61
3633.84
3626.36
3618.51
3607.66
3599.82
3591.88
3590.35
3583.03
3575.74
3536.40
3528.96
Vertically reduced
1700.51
1694.06
1687.48
1673.04
1274.17
1269.12
1265.59
1263.66
1261.73
1258.23
1256.39
1254.62
1252.80
1250.97
Vertically reduced
ppm

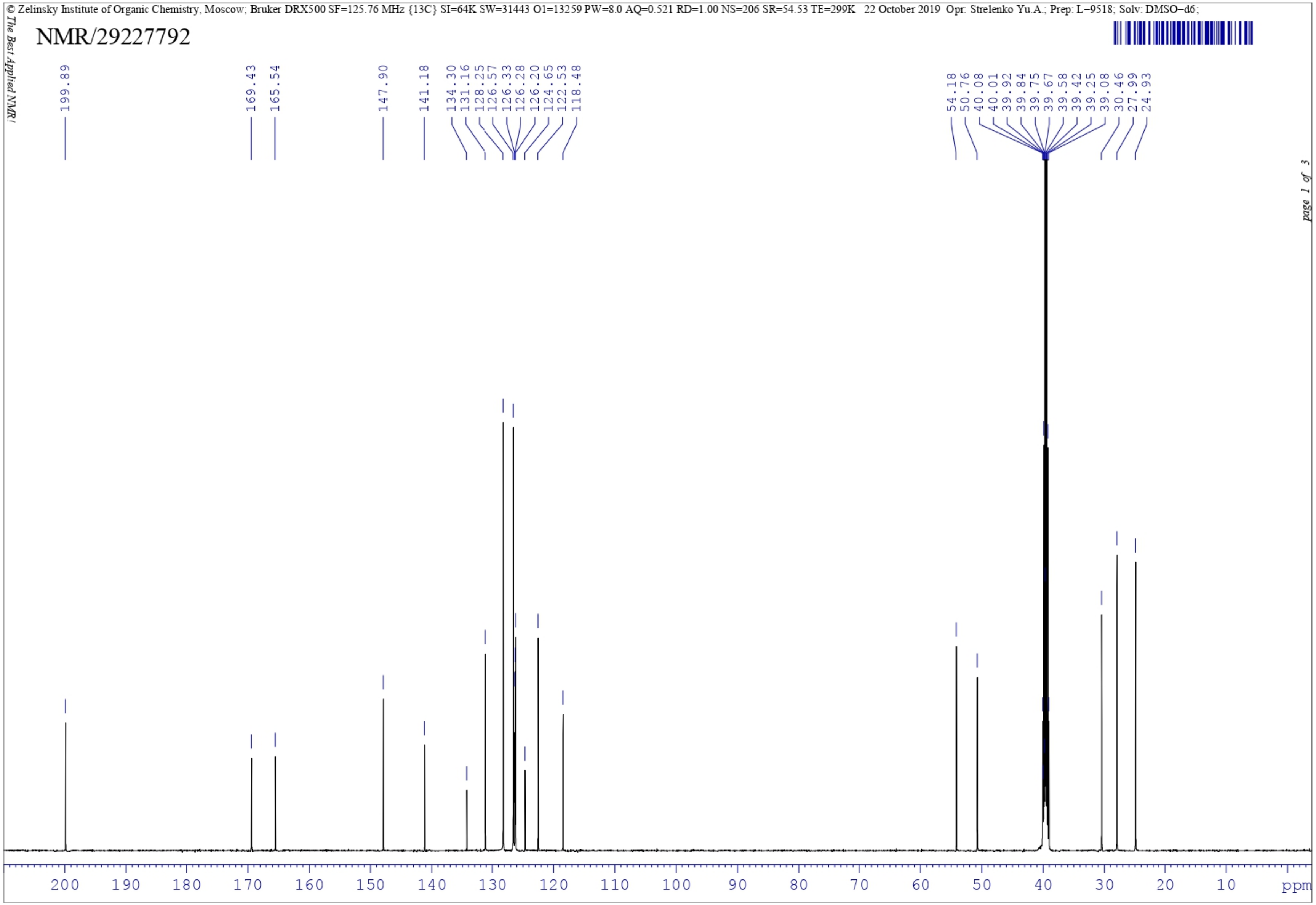
© Zelinsky Institute of Organic Chemistry, Moscow; Bruker DRX500 SF=125.76 MHz {13C} SI=64K SW=31443 O1=13259 PW=8.0 AQ=0.521 RD=1.00 NS=206 SR=54.53 TE=299K 22 October 2019 Opr: Strelenko Yu.A.; Prep: L−9518; Solv: DMSO−d6;
The Best Applied NMR!
NMR/29227792
199.89
169.43
165.54
147.90
141.18
134.30
131.16
128.25
126.57
126.33
126.28
126.20
124.65
122.53
118.48
54.18
50.76
40.08
40.01
39.92
39.84
39.75
39.67
39.58
39.42
39.25
39.08
30.46
27.99
24.93
page 1 of 3
200 190 180 170 160 150 140 130 120 110 100 90 80 70 60 50 40 30 20 10 ppm

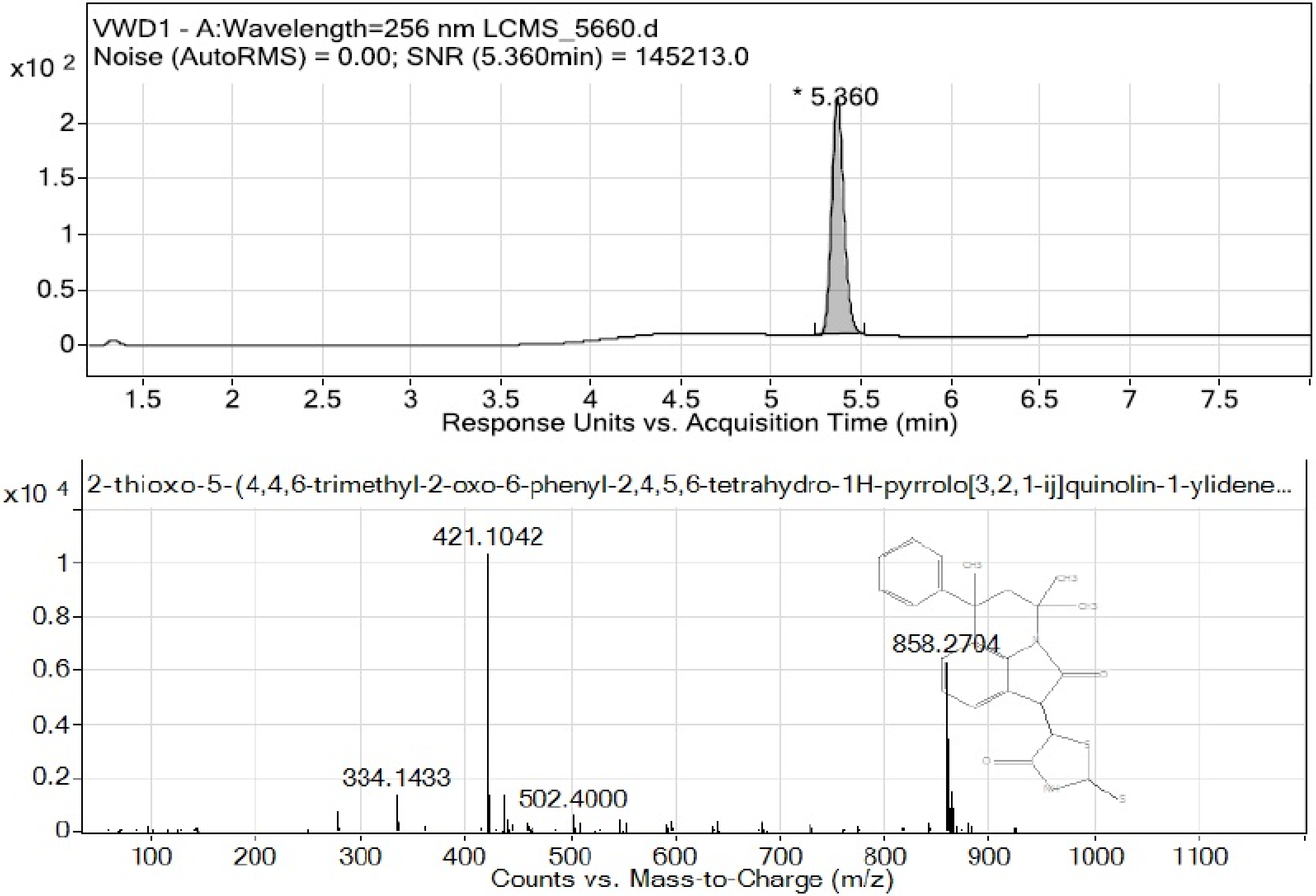
VWD1 - A:Wavelength=256 nm LCMS_5660.d
Noise (AutoRMS) = 0.00; SNR (5.360min) = 145213.0
x10 2
* 5.360
Response Units vs. Acquisition Time (min)
2-thioxo-5-(4,4,6-trimethyl-2-oxo-6-phenyl-2,4,5,6-tetrahydro-1H-pyrrolo[3,2,1-ij]quinolin-1-ylidene...
x10 4
421.1042
858.2704
334.1433
502.4000
Counts vs. Mass-to-Charge (m/z)

(*Z*)-5-(4,4,6,9-tetramethyl-2-oxo-6-phenyl-2,4,5,6-tetrahydro-1*H*-pyrrolo[3,2,1-*ij*]quinolin-1-ylidene)-2-thioxothiazolidin-4-one 7b

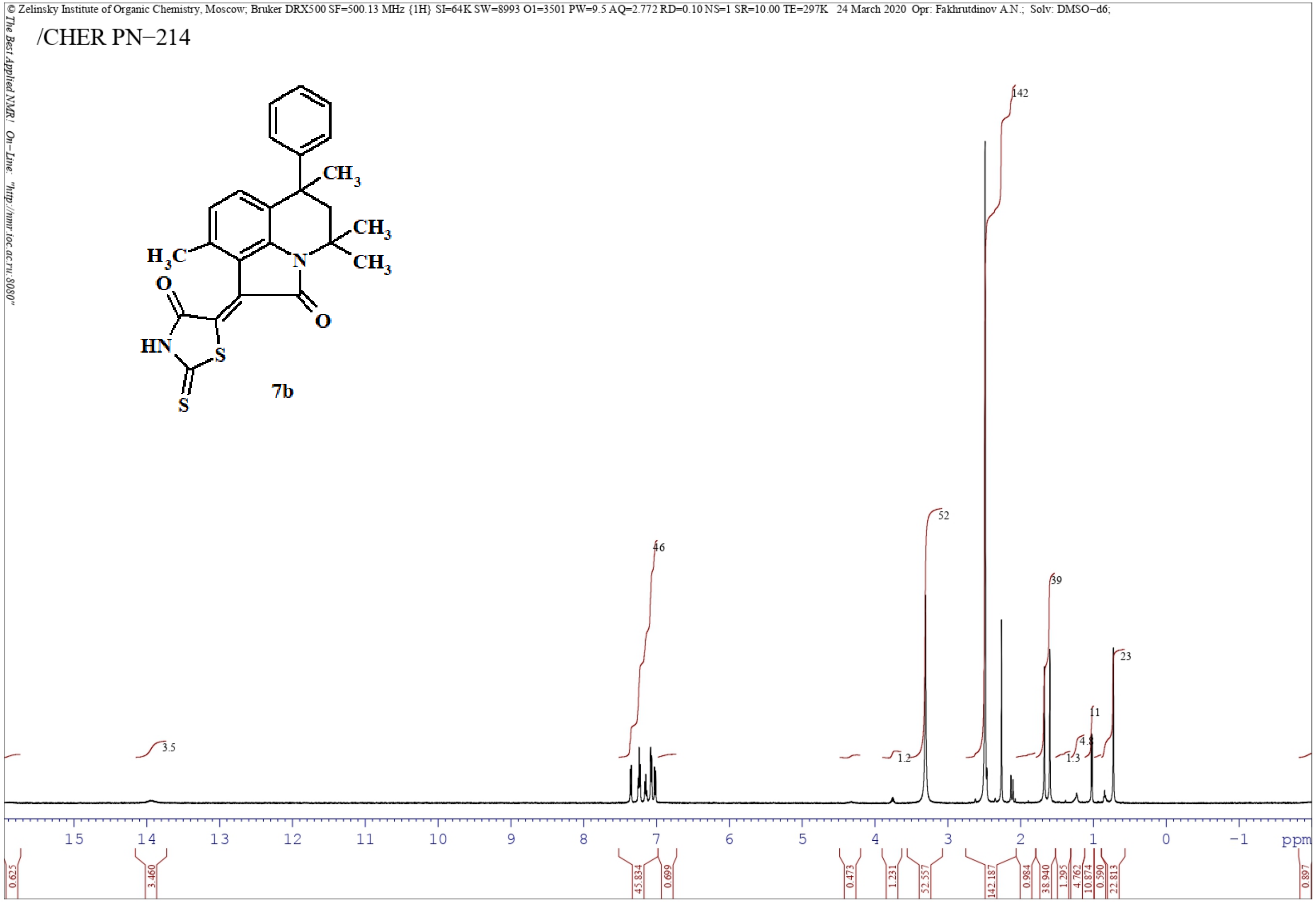

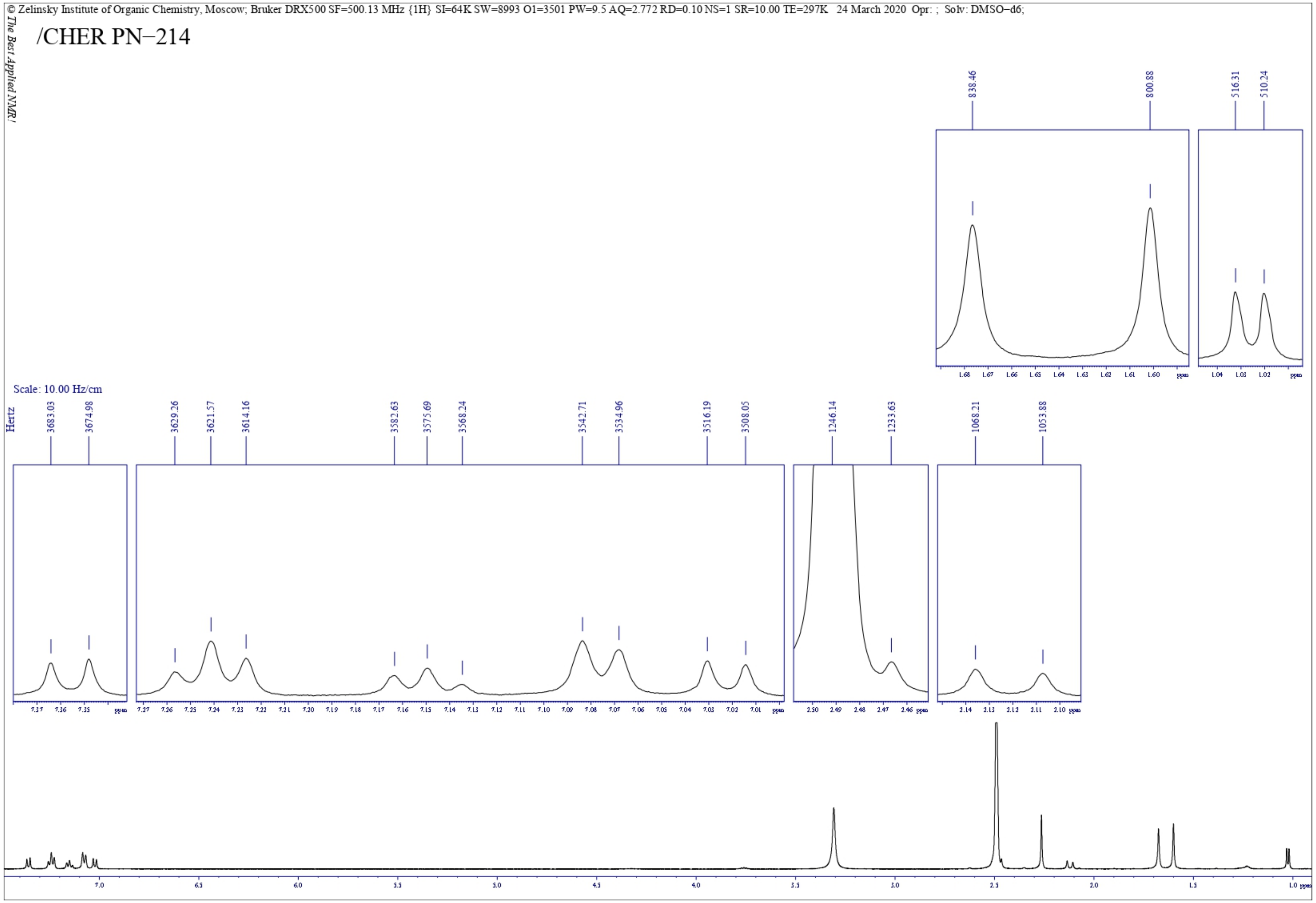
© Zelinsky Institute of Organic Chemistry, Moscow; Bruker DRX500 SF=500.13 MHz {1H} SI=64K SW=8993 O1=3501 PW=9.5 AQ=2.772 RD=0.10 NS=1 SR=10.00 TE=297K 24 March 2020 Opr: ; Solv: DMSO−d6;
The Best Applied NMR!
/CHER PN−214
Scale: 10.00 Hz/cm
Hertz
3683.03
3674.98
3629.26
3621.57
3614.16
3582.63
3575.69
3568.24
3542.71
3534.96
3516.19
3508.05
1246.14
1233.63
1068.21
1053.88
838.46
800.88
516.31
510.24

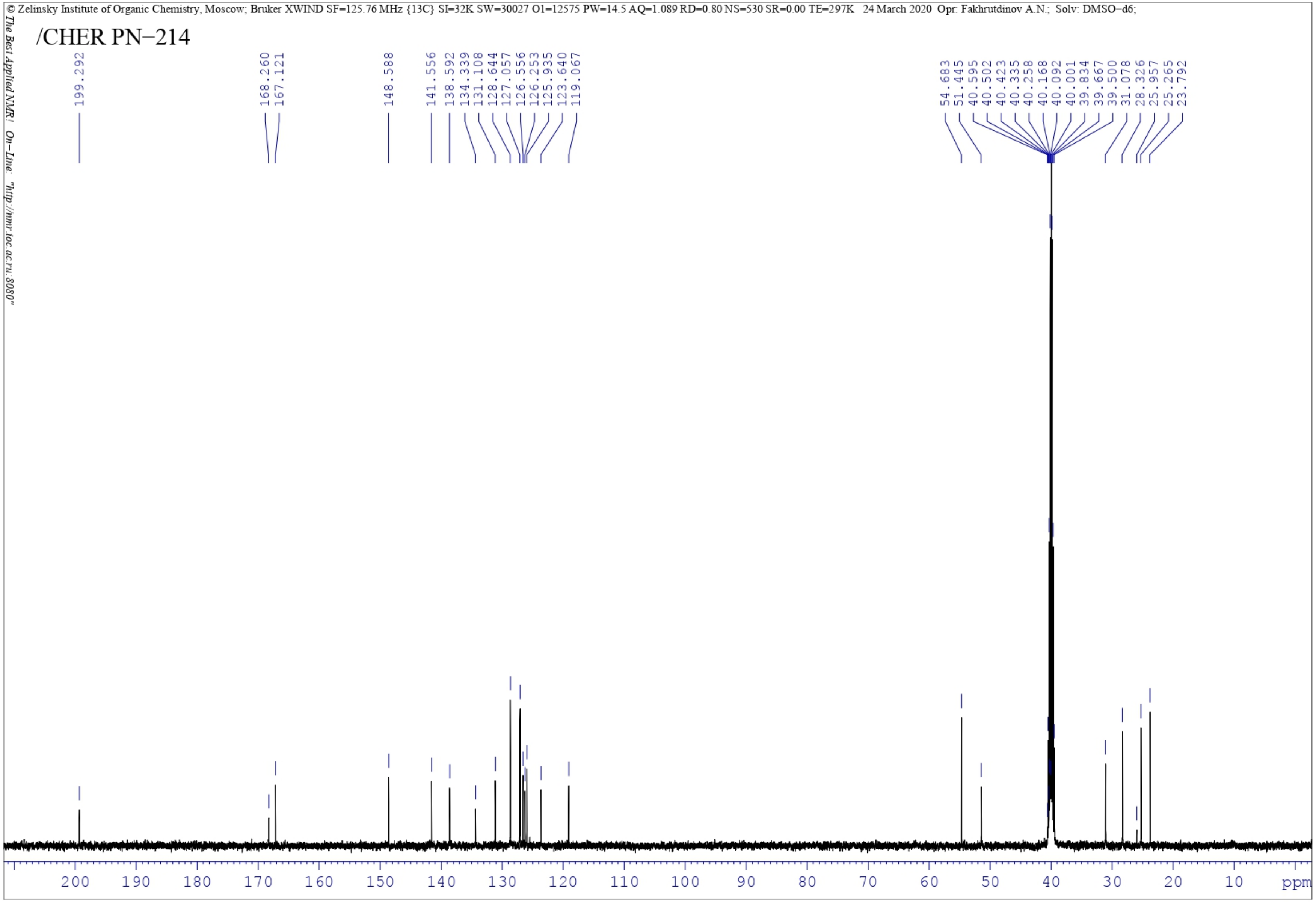
© Zelinsky Institute of Organic Chemistry, Moscow; Bruker XWIND SF=125.76 MHz {13C} SI=32K SW=30027 O1=12575 PW=14.5 AQ=1.089 RD=0.80 NS=530 SR=0.00 TE=297K 24 March 2020 Opr: Fakhrutdinov A.N.; Solv: DMSO−d6;
The Best Applied NMR! On−Line: "http://nmr.ioc.ac.ru:8080"
/CHER PN−214
199.292
168.260
167.121
148.588
141.556
138.592
134.339
131.108
128.644
127.057
126.556
126.253
125.935
123.640
119.067
54.683
51.445
40.595
40.502
40.423
40.335
40.258
40.168
40.092
40.001
39.834
39.667
39.500
31.078
28.326
25.957
25.265
23.792
200 190 180 170 160 150 140 130 120 110 100 90 80 70 60 50 40 30 20 10 ppm

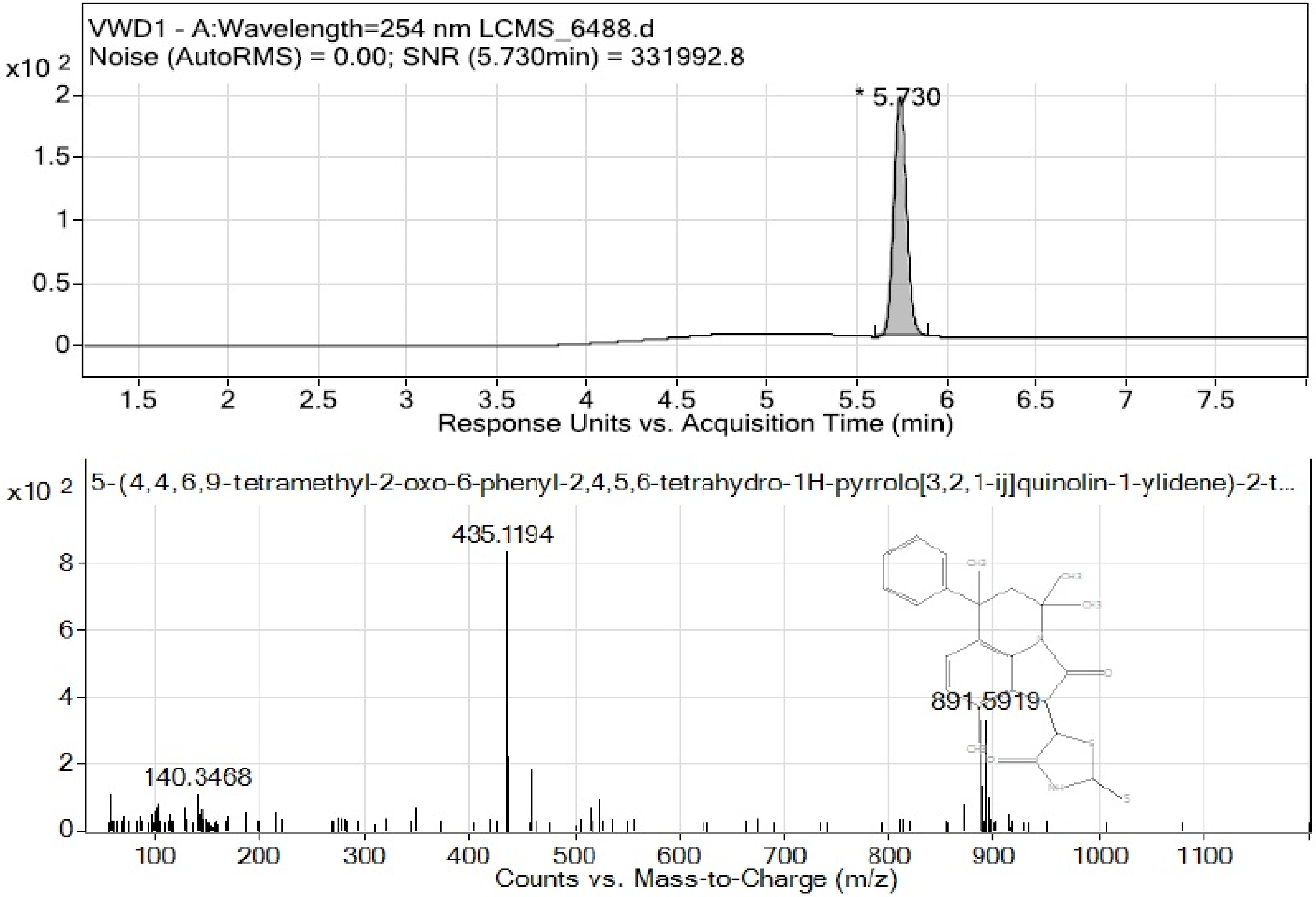
VWD1 - A:Wavelength=254 nm LCMS_6488.d
Noise (AutoRMS) = 0.00; SNR (5.730min) = 331992.8
x10 2
* 5.730
Response Units vs. Acquisition Time (min)
x10 2
5-(4,4,6,9-tetramethyl-2-oxo-6-phenyl-2,4,5,6-tetrahydro-1H-pyrrolo[3,2,1-ij]quinolin-1-ylidene)-2-t...
435.1194
891.5919
140.3468
Counts vs. Mass-to-Charge (m/z)

(*Z*)-5-(6-(4-chlorophenyl)-4,4,6,8-tetramethyl-2-oxo-2,4,5,6-tetrahydro-1*H*-pyrrolo[3,2,1-*ij*]quinolin-1-ylidene)-2-thioxothiazolidin-4-one 7c

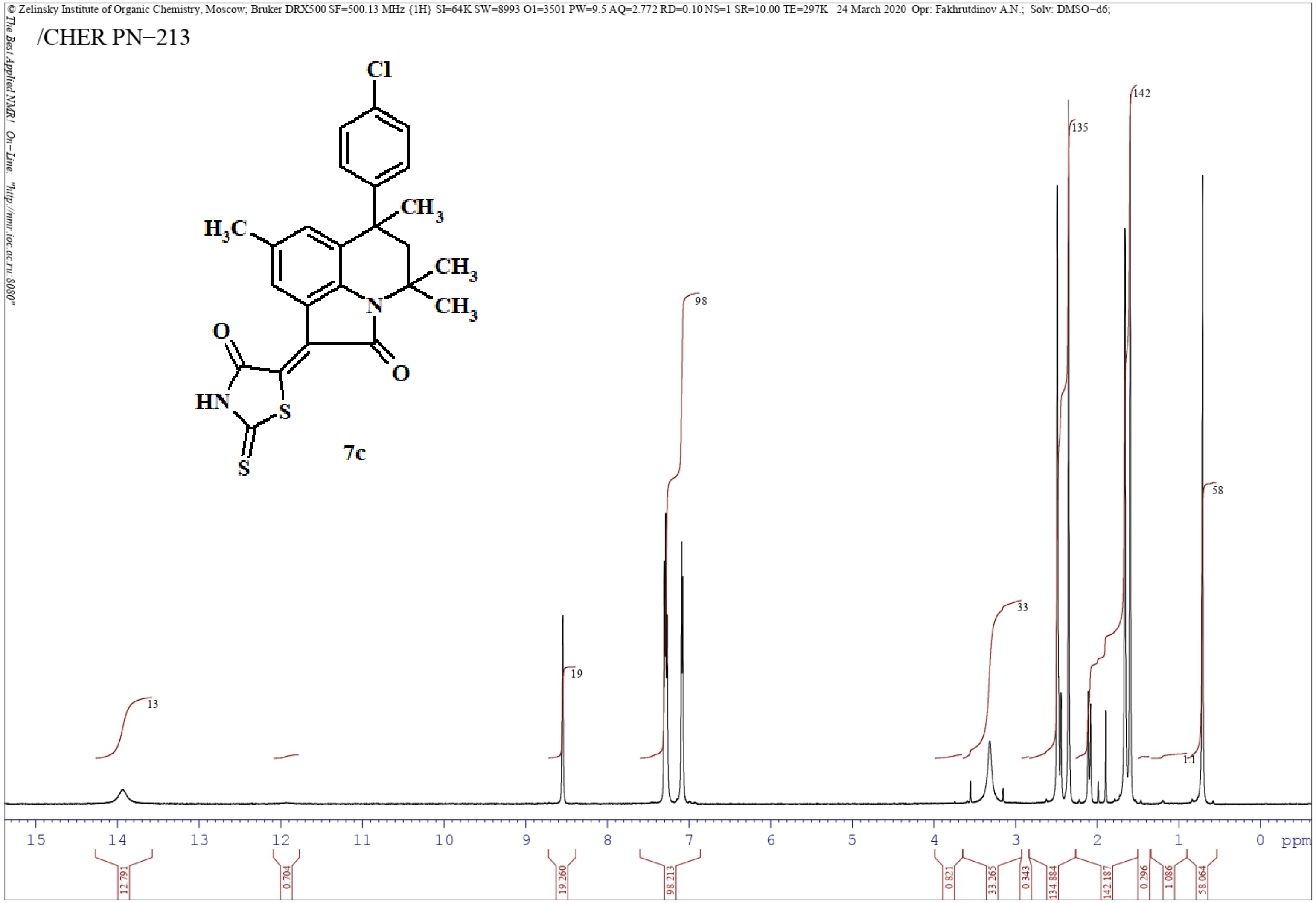

© Zelinsky Institute of Organic Chemistry, Moscow; Bruker DRX500 SF=500.13 MHz {1H} SI=64K SW=8993 O1=3501 PW=9.5 AQ=2.772 RD=0.10 NS=1 SR=10.00 TE=297K 24 March 2020 Opr: ; Solv: DMSO−d6;

/CHER PN−213/

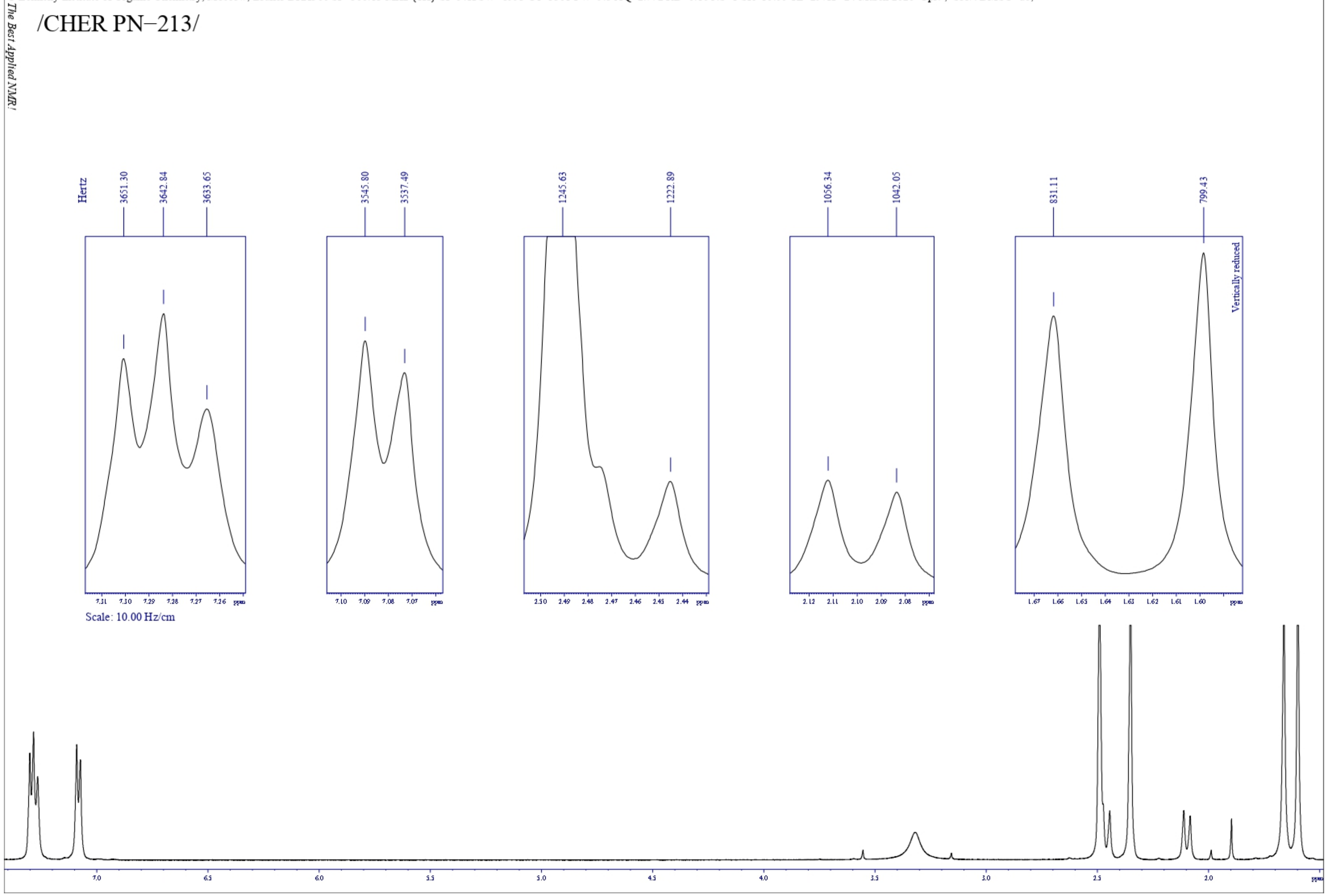

© Zelinsky Institute of Organic Chemistry, Moscow; Bruker XWIND SF=125.76 MHz {13C} SI=32K SW=30027 O1=12575 PW=14.5 AQ=1.089 RD=0.80 NS=436 SR=0.00 TE=297K 24 March 2020 Opr: Fakhrutdinov A.N.; Solv: DMSO-d6;

/CHER PN−213

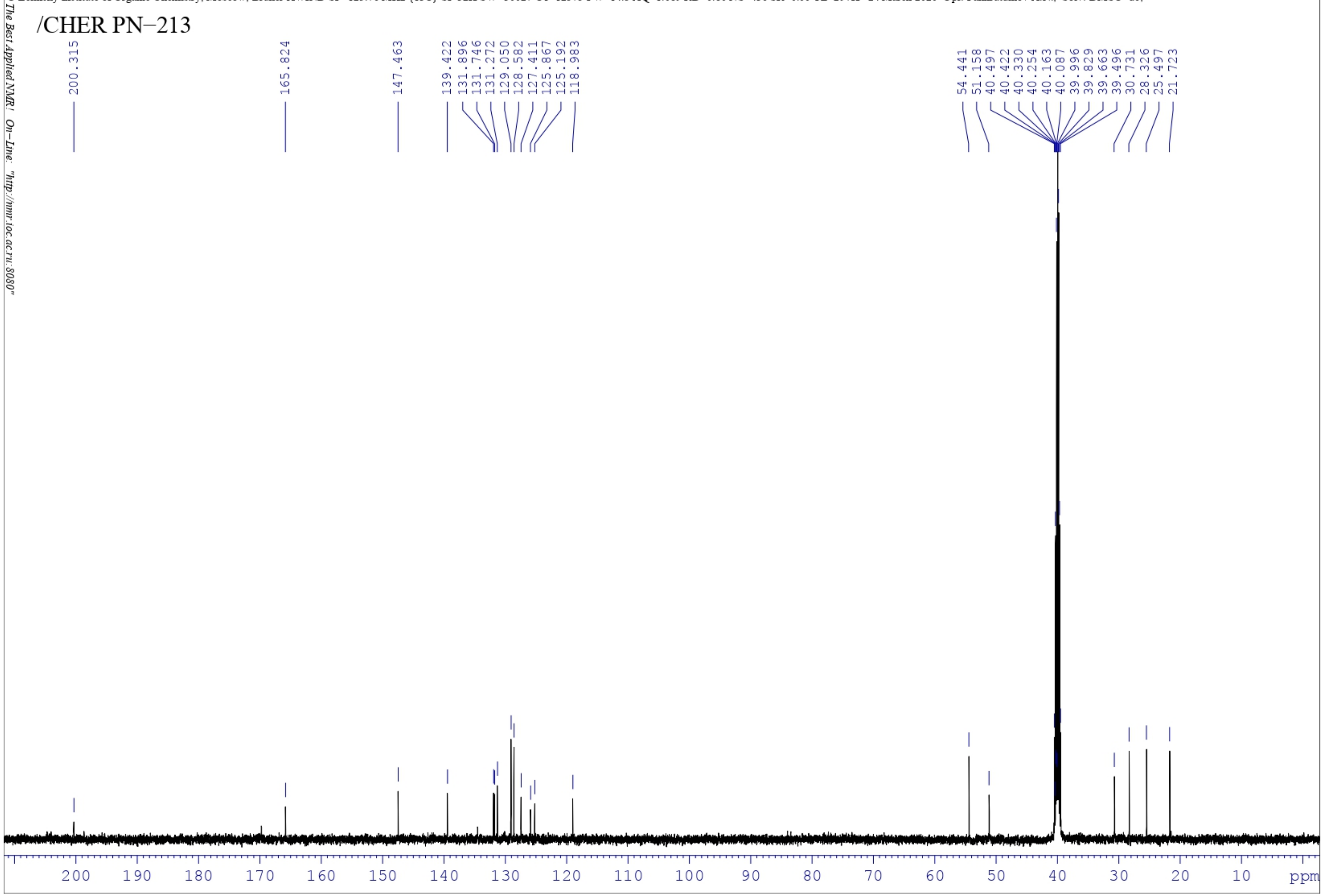

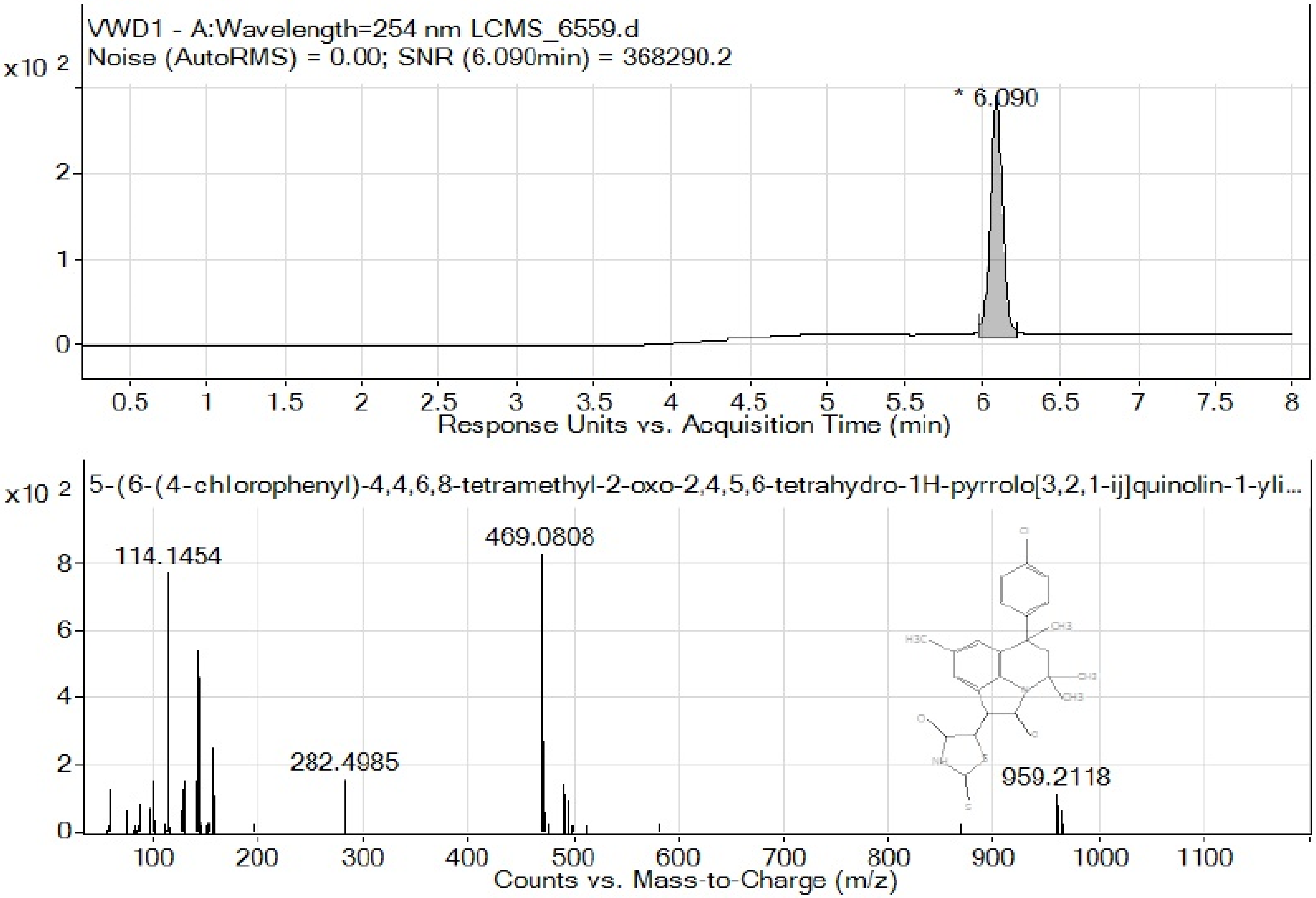
VWD1 - A:Wavelength=254 nm LCMS_6559.d
Noise (AutoRMS) = 0.00; SNR (6.090min) = 368290.2
x10 2
* 6.090
Response Units vs. Acquisition Time (min)
x10 2
5-(6-(4-chlorophenyl)-4,4,6,8-tetramethyl-2-oxo-2,4,5,6-tetrahydro-1H-pyrrolo[3,2,1-ij]quinolin-1-yli...
114.1454
469.0808
282.4985
959.2118
Counts vs. Mass-to-Charge (m/z)

^{1}H, ^{13}C NMR spectra and data of HPLC-MS-ESI analysis of PQD 14

Ethyl 4-((8-methoxy-4,4-dimethyl-1,2-dioxo--2,4-dihydro-1*H*-pyrrolo[3,2,1-*ij*]quinolin-6-yl)methyl)piperazine-1-carboxylate 14a

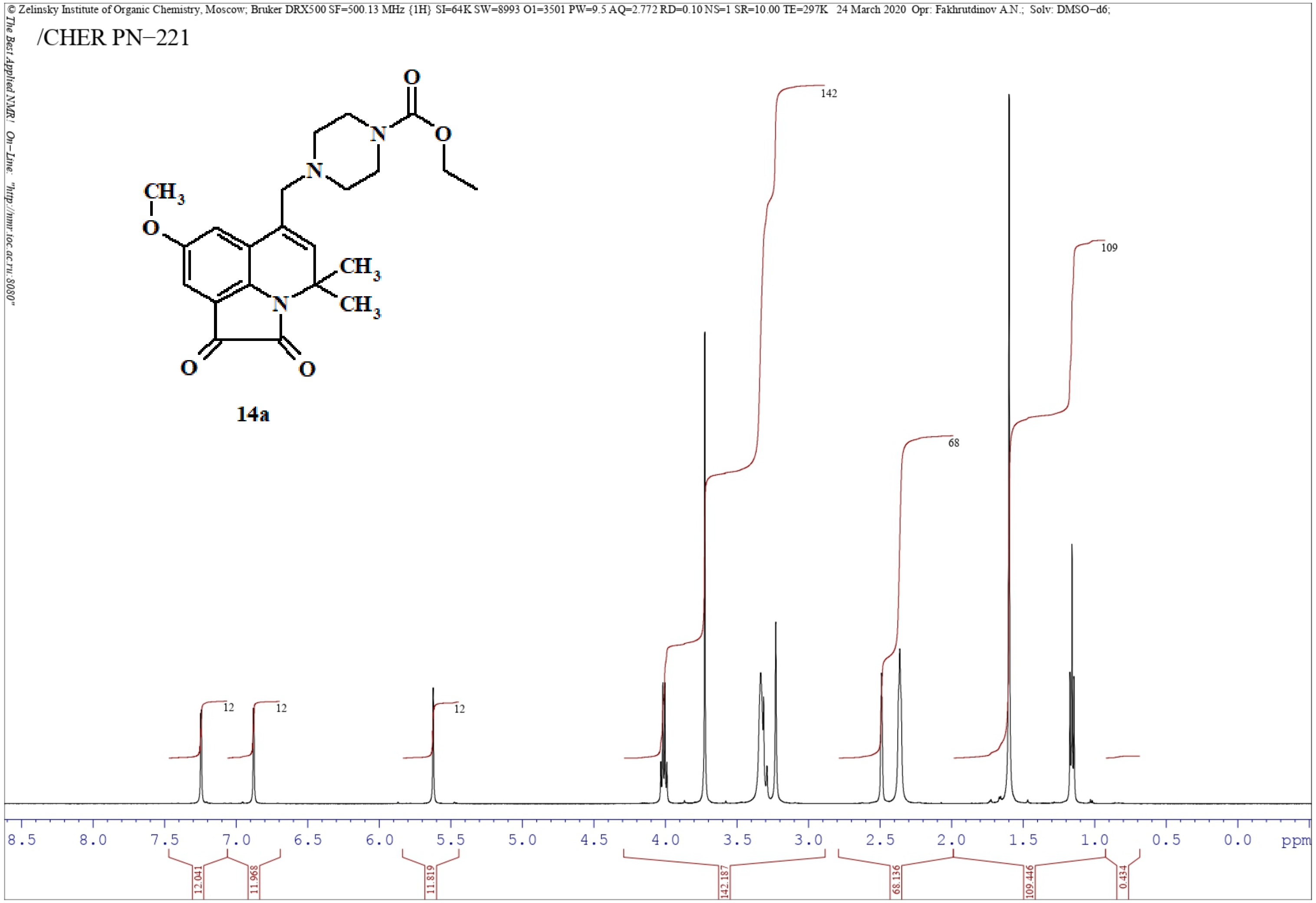

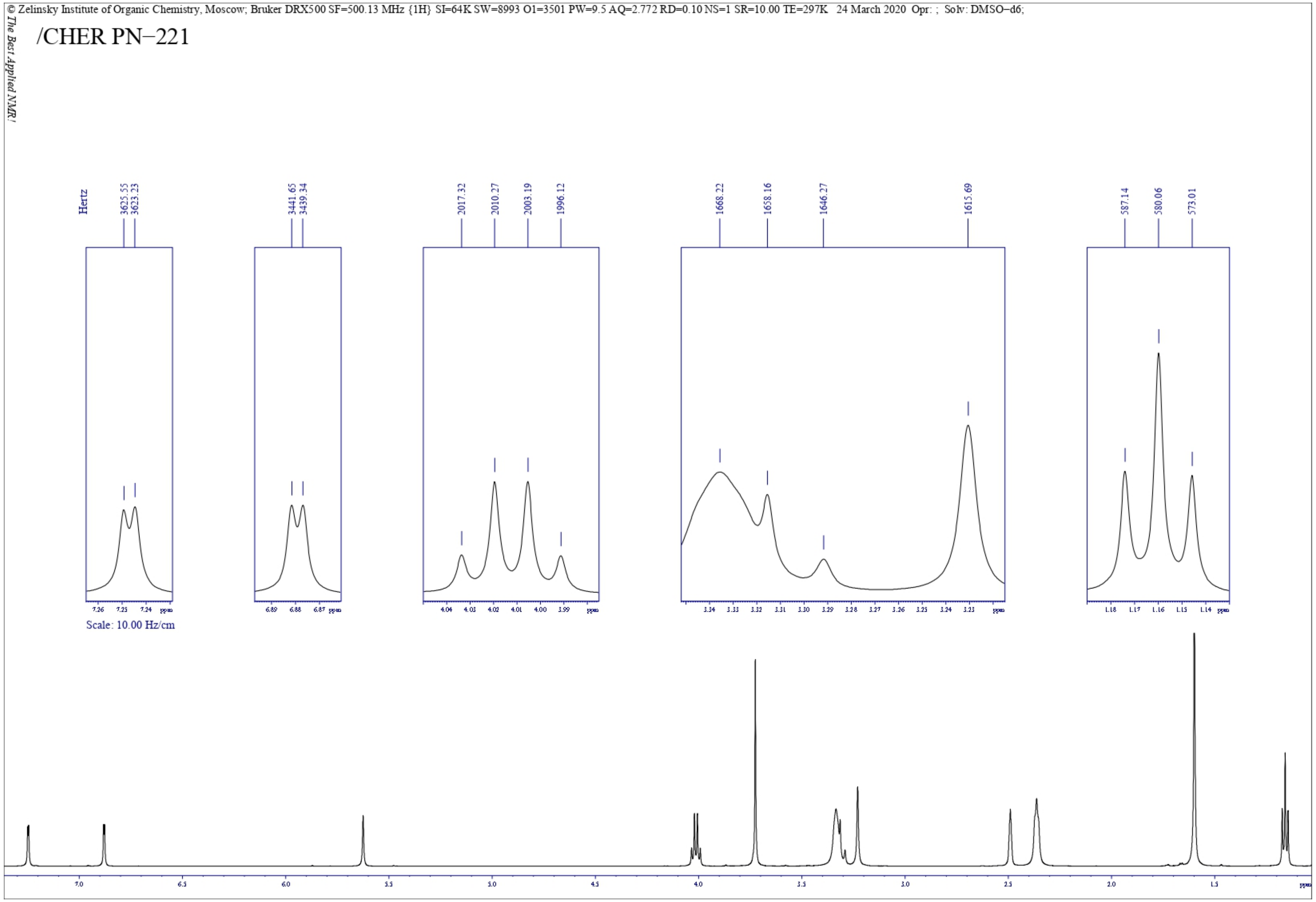
© Zelinsky Institute of Organic Chemistry, Moscow; Bruker DRX500 SF=500.13 MHz {1H} SI=64K SW=8993 O1=3501 PW=9.5 AQ=2.772 RD=0.10 NS=1 SR=10.00 TE=297K 24 March 2020 Opr: ; Solv: DMSO−d6;
The Best Applied NMR!
/CHER PN−221
Hertz
3625.55
3623.23
3441.65
3439.34
2017.32
2010.27
2003.19
1996.12
1668.22
1658.16
1646.27
1615.69
587.14
580.06
573.01
Scale: 10.00 Hz/cm

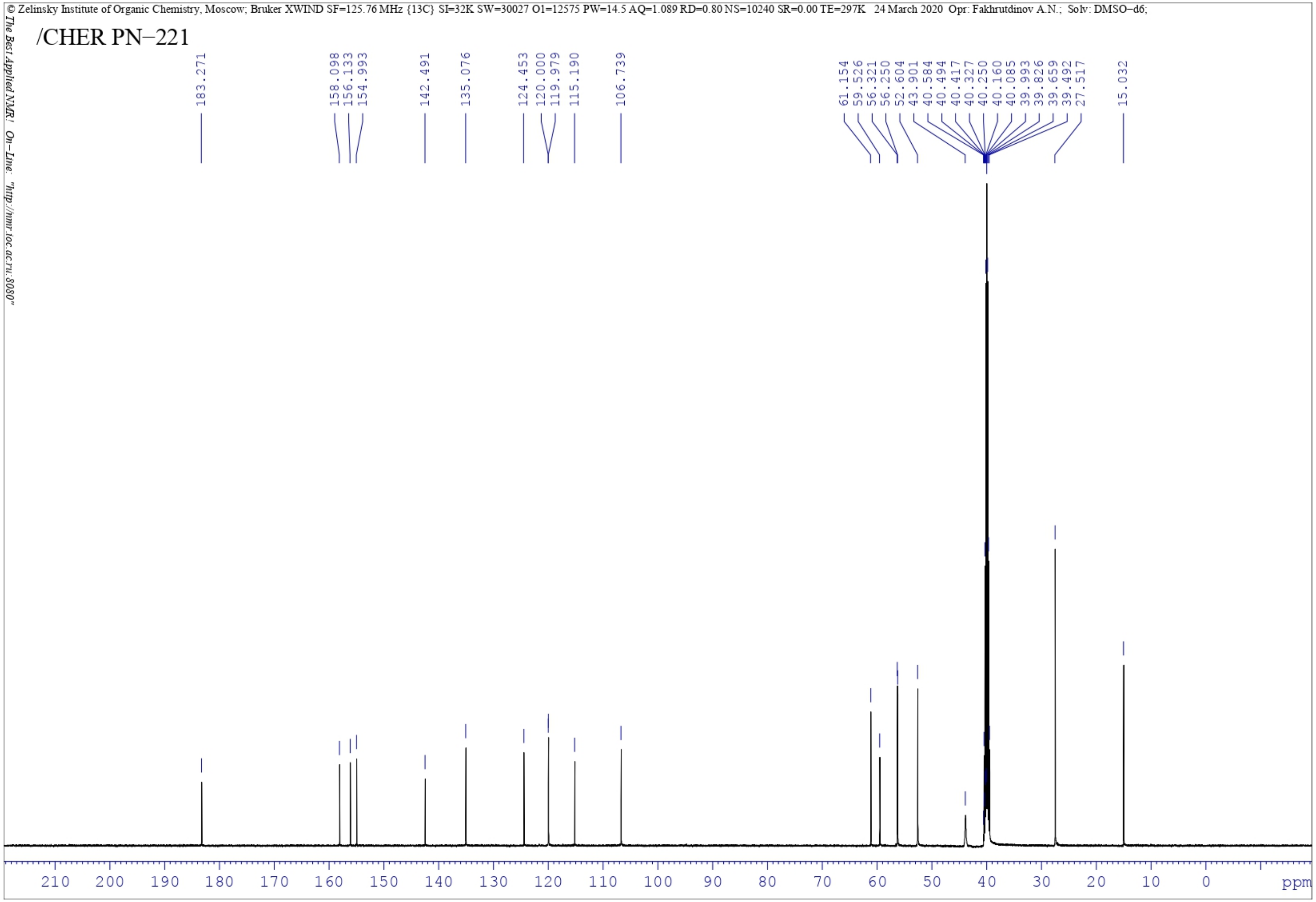
© Zelinsky Institute of Organic Chemistry, Moscow; Bruker XWIND SF=125.76 MHz {13C} SI=32K SW=30027 O1=12575 PW=14.5 AQ=1.089 RD=0.80 NS=10240 SR=0.00 TE=297K 24 March 2020 Opr: Fakhrutdinov A.N.; Solv: DMSO−d6;
The Best Applied NMR! On−Line: "http://nmr.ioc.ac.ru:8080"
/CHER PN−221
183.271
158.098
156.133
154.993
142.491
135.076
124.453
120.000
119.979
115.190
106.739
61.154
59.526
56.321
56.250
52.604
43.901
40.584
40.494
40.417
40.327
40.250
40.160
40.085
39.993
39.826
39.659
39.492
27.517
15.032
210 200 190 180 170 160 150 140 130 120 110 100 90 80 70 60 50 40 30 20 10 0 ppm

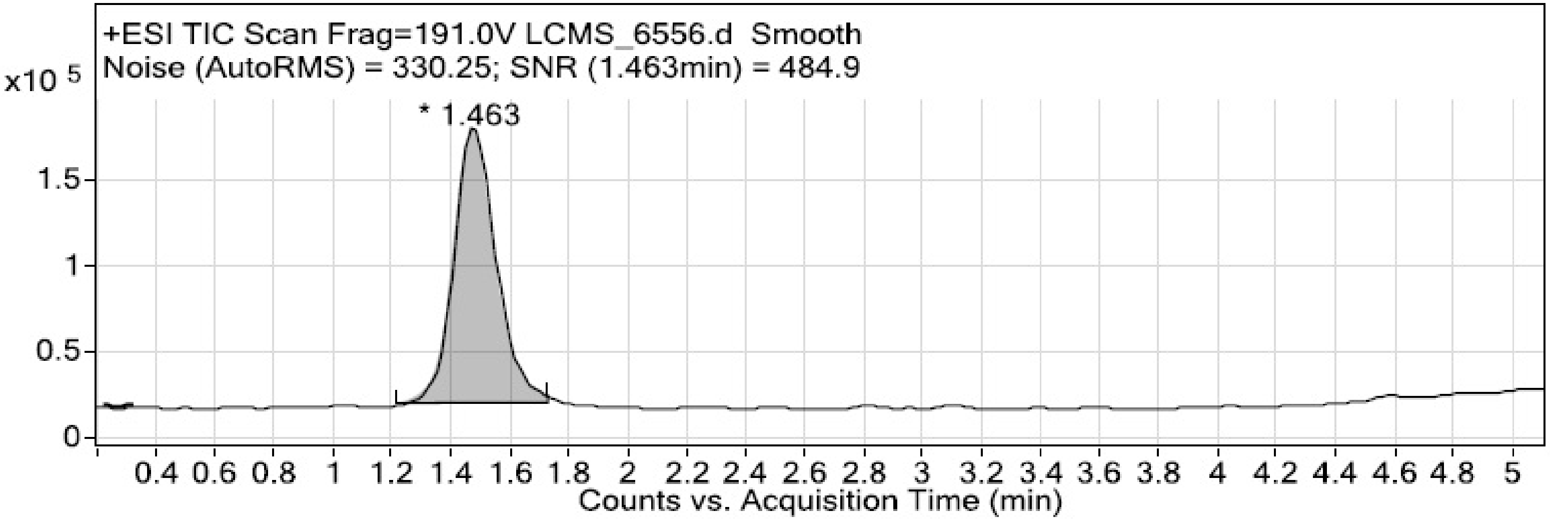
+ESI TIC Scan Frag=191.0V LCMS_6556.d Smooth
Noise (AutoRMS) = 330.25; SNR (1.463min) = 484.9
x10 5
* 1.463
1.5
1
0.5
0
0.4 0.6 0.8 1 1.2 1.4 1.6 1.8 2 2.2 2.4 2.6 2.8 3 3.2 3.4 3.6 3.8 4 4.2 4.4 4.6 4.8 5
Counts vs. Acquisition Time (min)

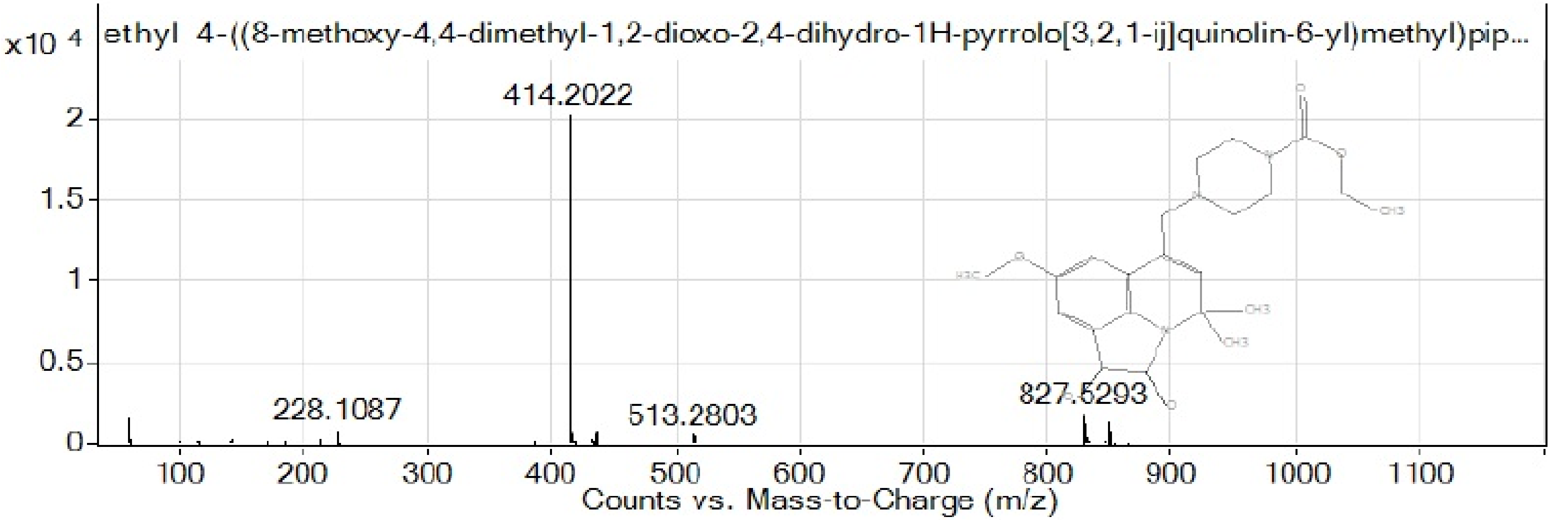
x10 4
ethyl 4-((8-methoxy-4,4-dimethyl-1,2-dioxo-2,4-dihydro-1H-pyrrolo[3,2,1-ij]quinolin-6-yl)methyl)pip...
414.2022
228.1087
513.2803
827.5293
2
1.5
1
0.5
0
100 200 300 400 500 600 700 800 900 1000 1100
Counts vs. Mass-to-Charge (m/z)

8-Methoxy-4,4-dimethyl-6-((4-phenylpiperazine-1-yl)methyl)-1*H*-pyrrolo[3,2,1-*ij*]quinolin-1,2(4*H*)-dione 14b

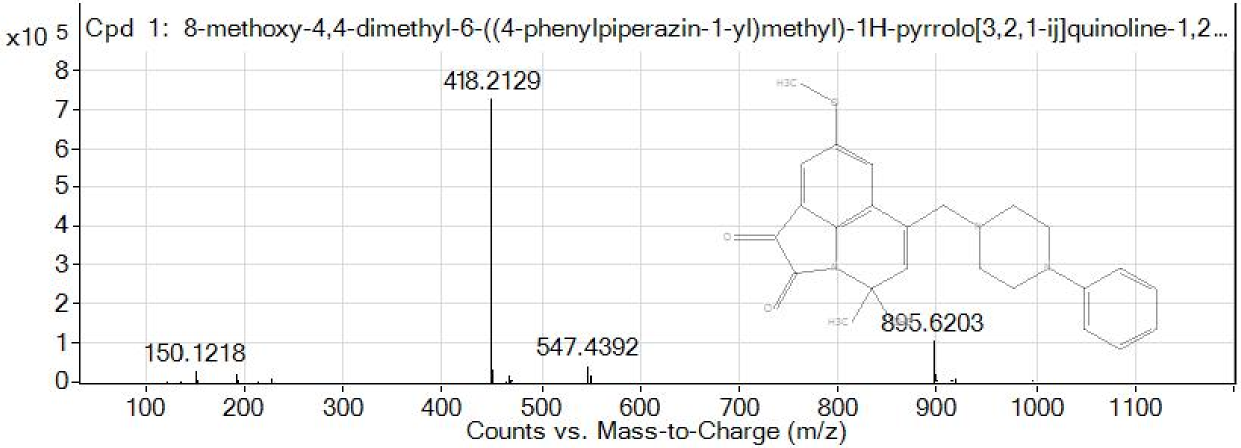

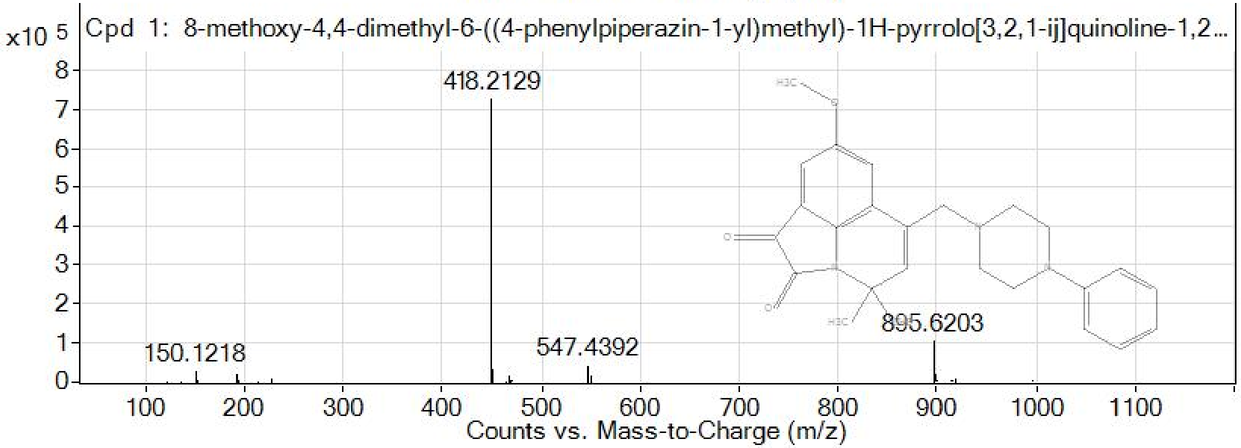

Ethyl 4-((8-fluoro-4,4-dimethyl-1,2-dioxo-2,4-dihydro-1*H*-pyrrolo[3,2,1-*ij*]quinolin-6-yl)methyl)piperazine-1-carboxylate 14c

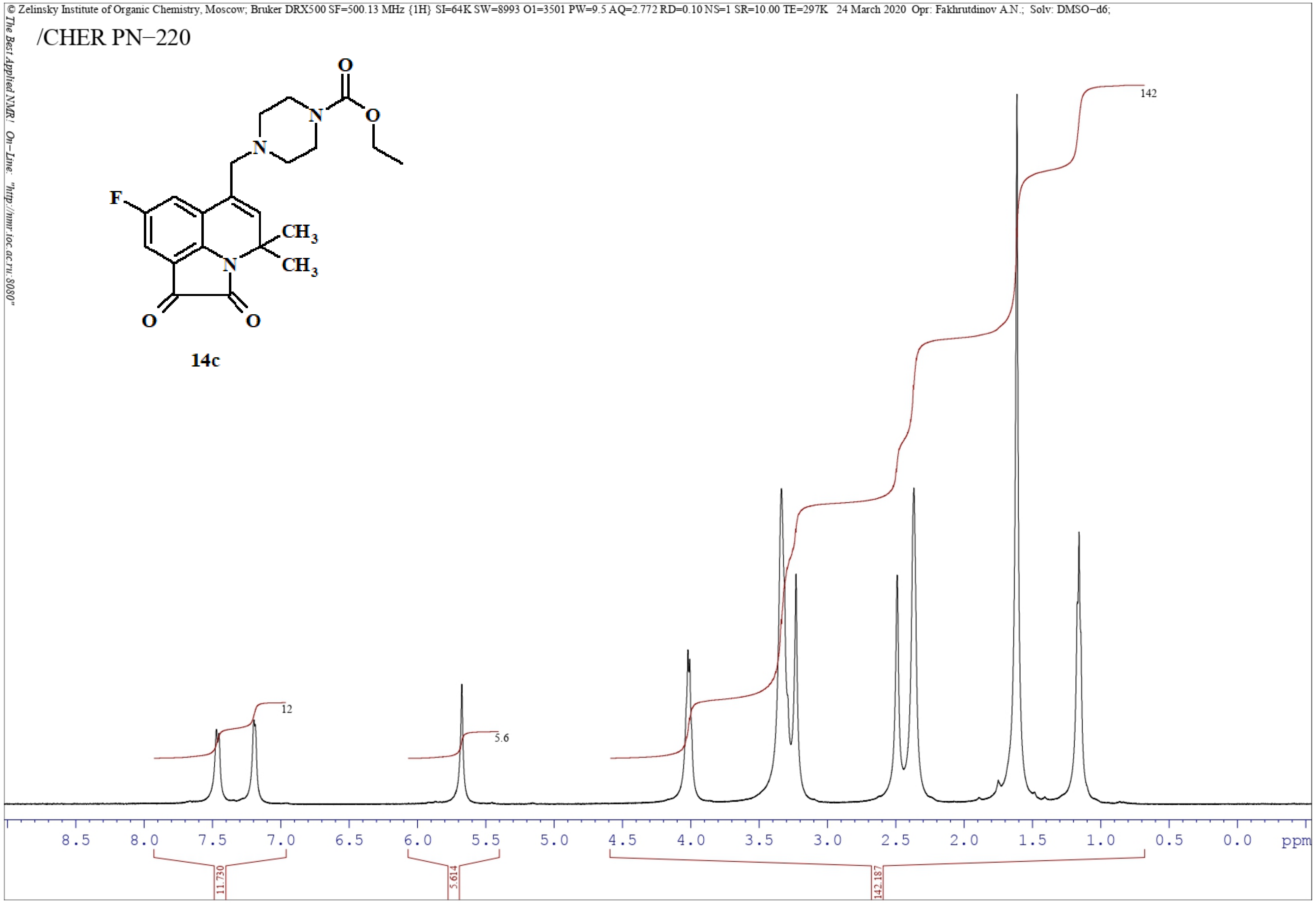

© Zelinsky Institute of Organic Chemistry, Moscow; Bruker DRX500 SF=500.13 MHz {1H} SI=64K SW=8993 O1=3501 PW=9.5 AQ=2.772 RD=0.10 NS=1 SR=10.00 TE=297K 24 March 2020 Opr: ; Solv: DMSO−d6;

The Best Applied NMR!

/CHER PN−220

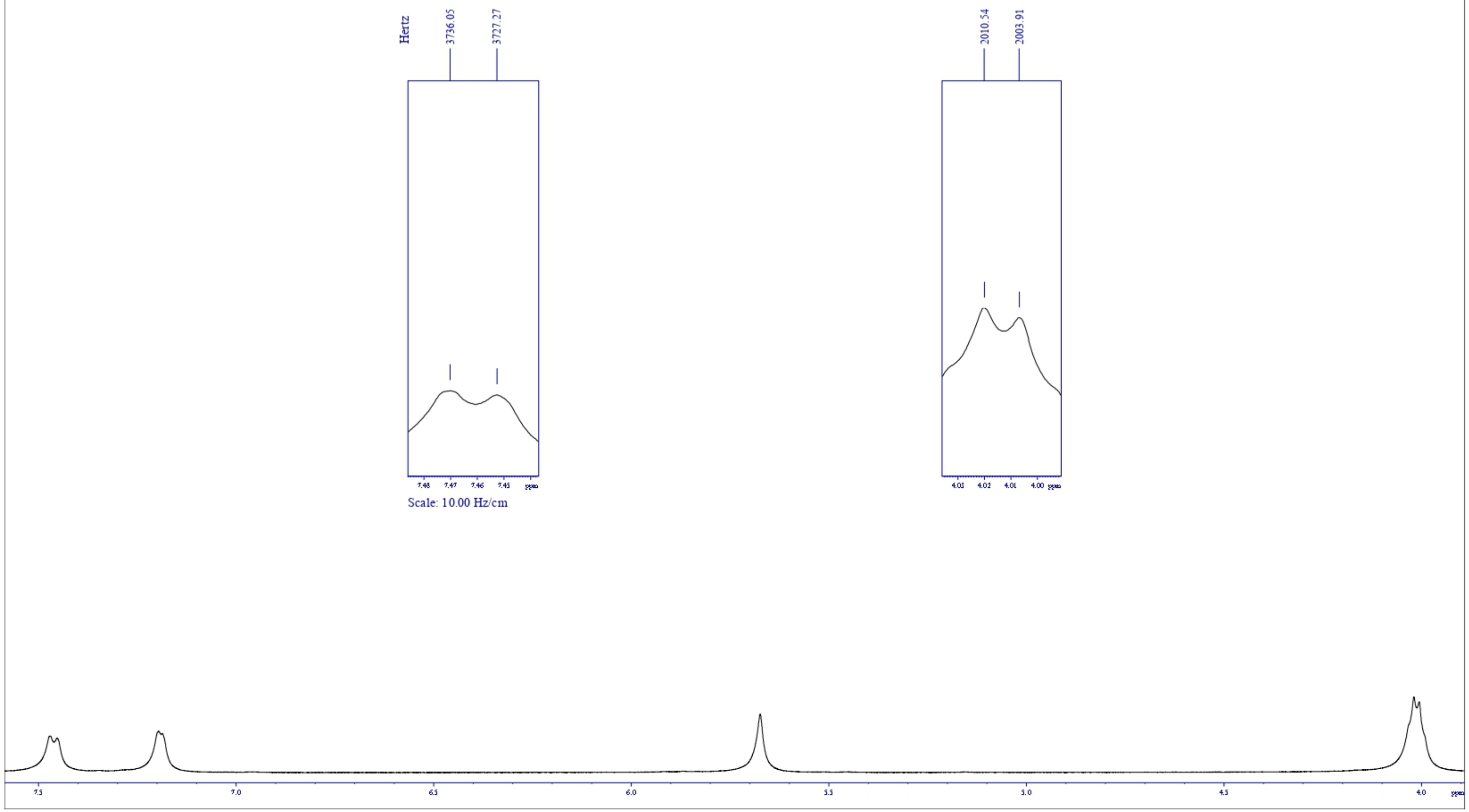

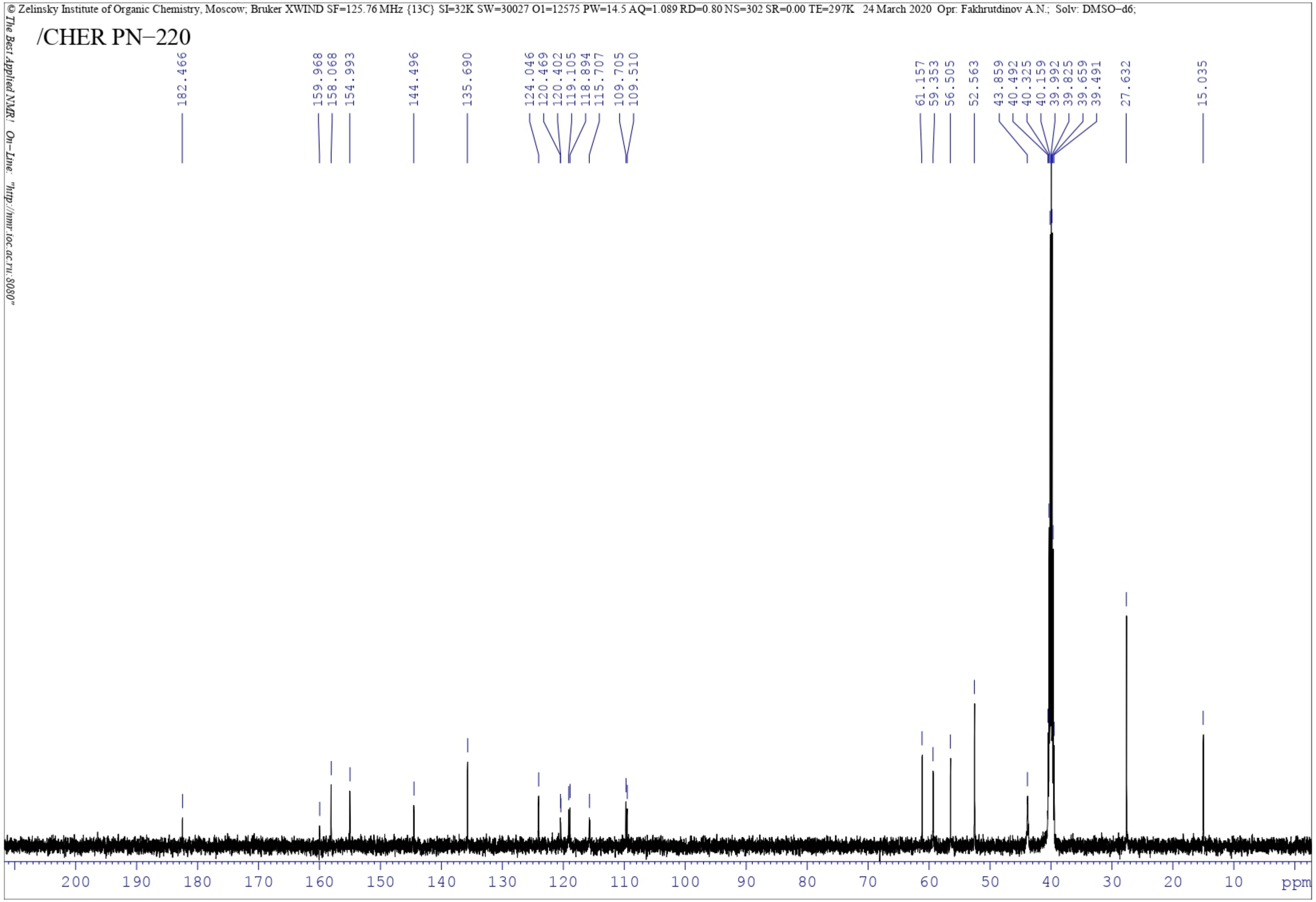
© Zelinsky Institute of Organic Chemistry, Moscow; Bruker XWIND SF=125.76 MHz {13C} SI=32K SW=30027 O1=12575 PW=14.5 AQ=1.089 RD=0.80 NS=302 SR=0.00 TE=297K 24 March 2020 Opr: Fakhrutdinov A.N.; Solv: DMSO−d6;
The Best Applied NMR! On−Line: "http://nmr.ioc.ac.ru:8080"
/CHER PN−220
182.466
159.968
158.068
154.993
144.496
135.690
124.046
120.469
120.402
119.105
118.894
115.707
109.705
109.510
61.157
59.353
56.505
52.563
43.859
40.492
40.325
40.159
39.992
39.825
39.659
39.491
27.632
15.035
200 190 180 170 160 150 140 130 120 110 100 90 80 70 60 50 40 30 20 10 ppm

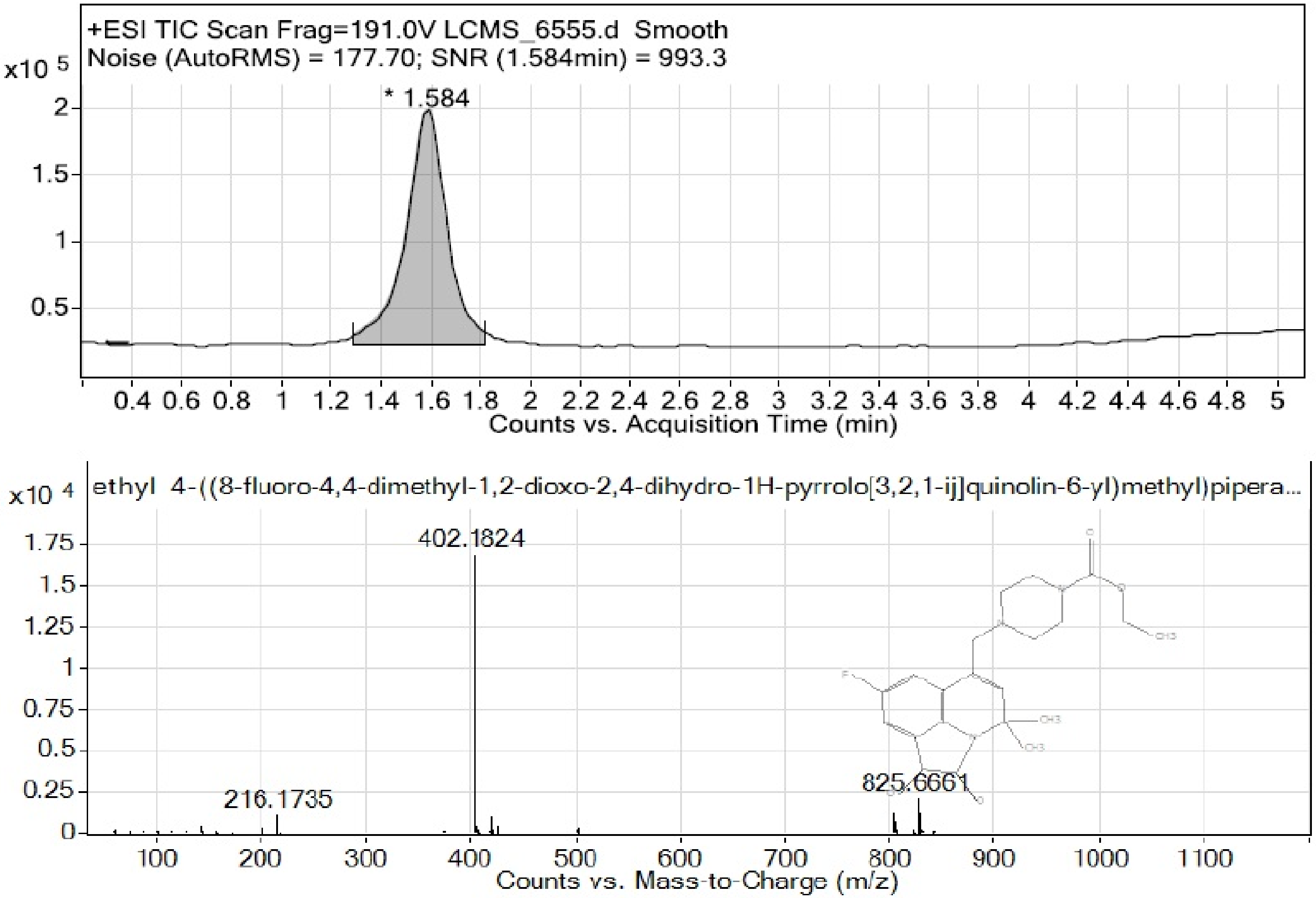

+ESI TIC Scan Frag=191.0V LCMS_6555.d Smooth
Noise (AutoRMS) = 177.70; SNR (1.584min) = 993.3
x10 5
* 1.584
Counts vs. Acquisition Time (min)
ethyl 4-((8-fluoro-4,4-dimethyl-1,2-dioxo-2,4-dihydro-1H-pyrrolo[3,2,1-ij]quinolin-6-yl)methyl)pipera...
x10 4
402.1824
216.1735
825.6661
Counts vs. Mass-to-Charge (m/z)

6-((4-benzo[*d*][1,3]dioxol-5-ylmethyl)piperazine-1-yl)methyl)-8-methoxy-4,4-dimethyl-1*H*-pyrrolo[3,2,1-*ij*]quinolin-1,2(4*H*)-dione

14d

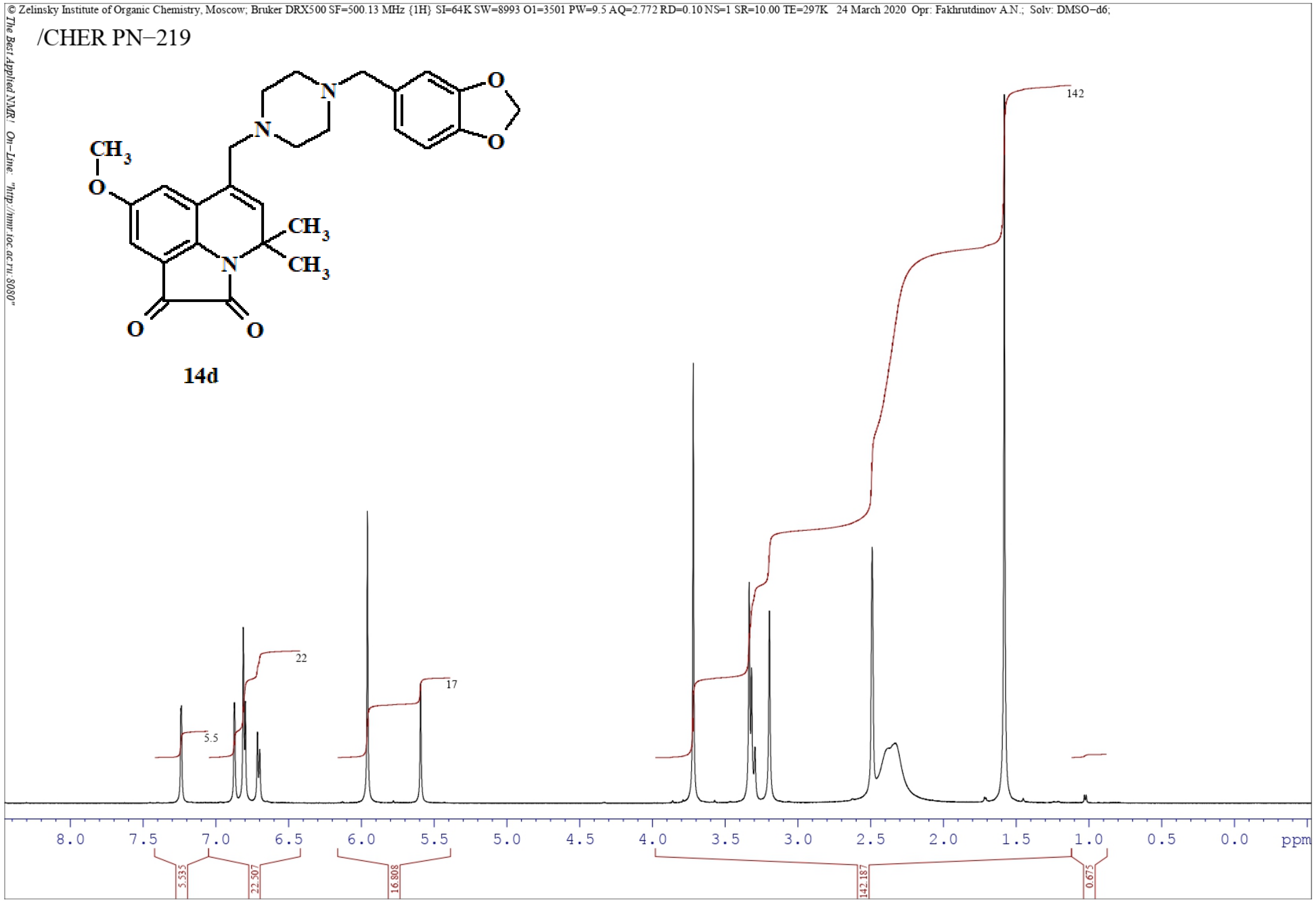

© Zelinsky Institute of Organic Chemistry, Moscow; Bruker DRX500 SF=500.13 MHz {1H} SI=64K SW=8993 O1=3501 PW=9.5 AQ=2.772 RD=0.10 NS=1 SR=10.00 TE=297K 24 March 2020 Opr: ; Solv: DMSO-d6;

/CHER PN−219

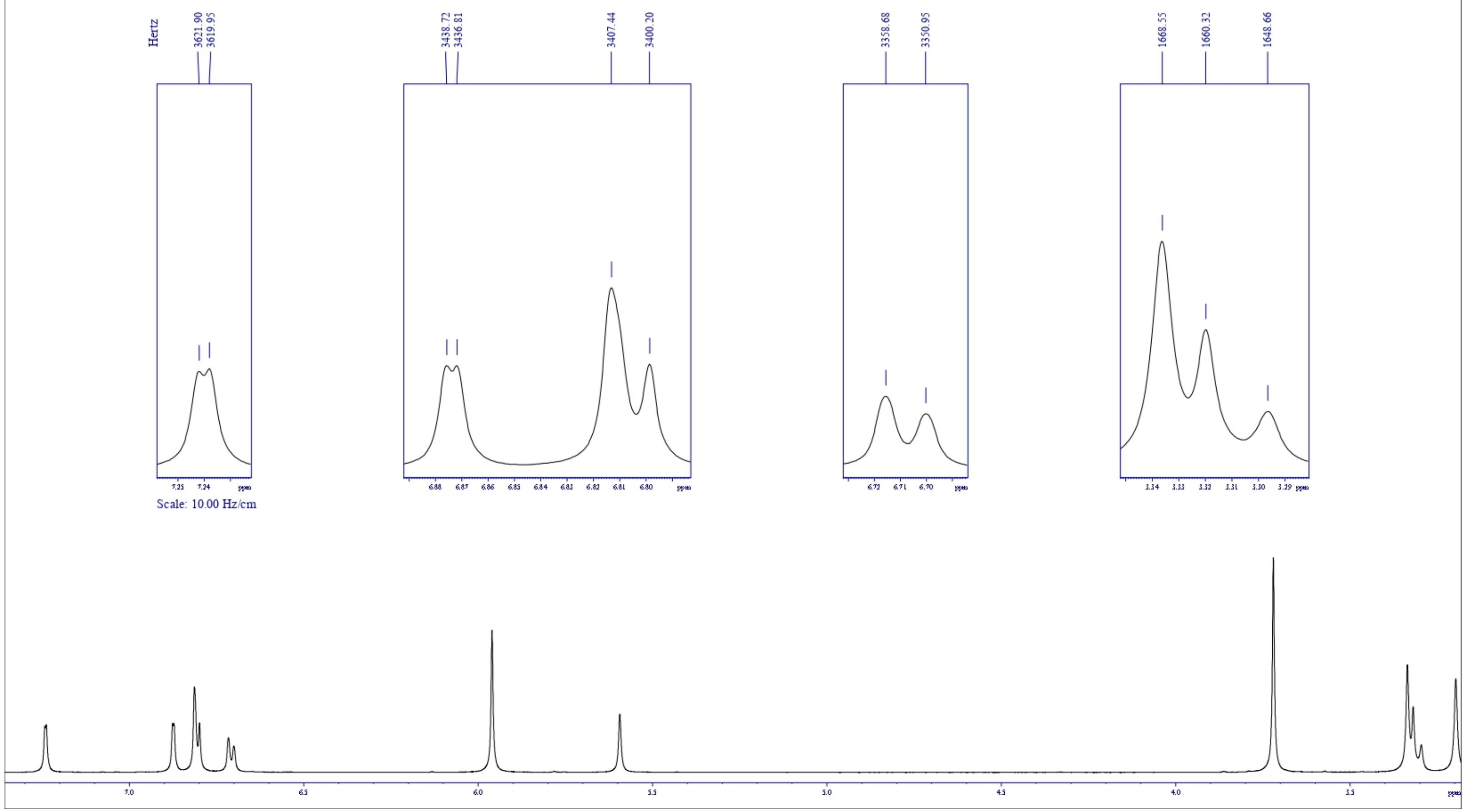

© Zelinsky Institute of Organic Chemistry, Moscow; Bruker XWIND SF=125.76 MHz {13C} SI=32K SW=30027 O1=12575 PW=14.5 AQ=1.089 RD=0.80 NS=641 SR=0.00 TE=297K 24 March 2020 Opr: Fakhrutdinov A.N.; Solv: DMSO−d6;

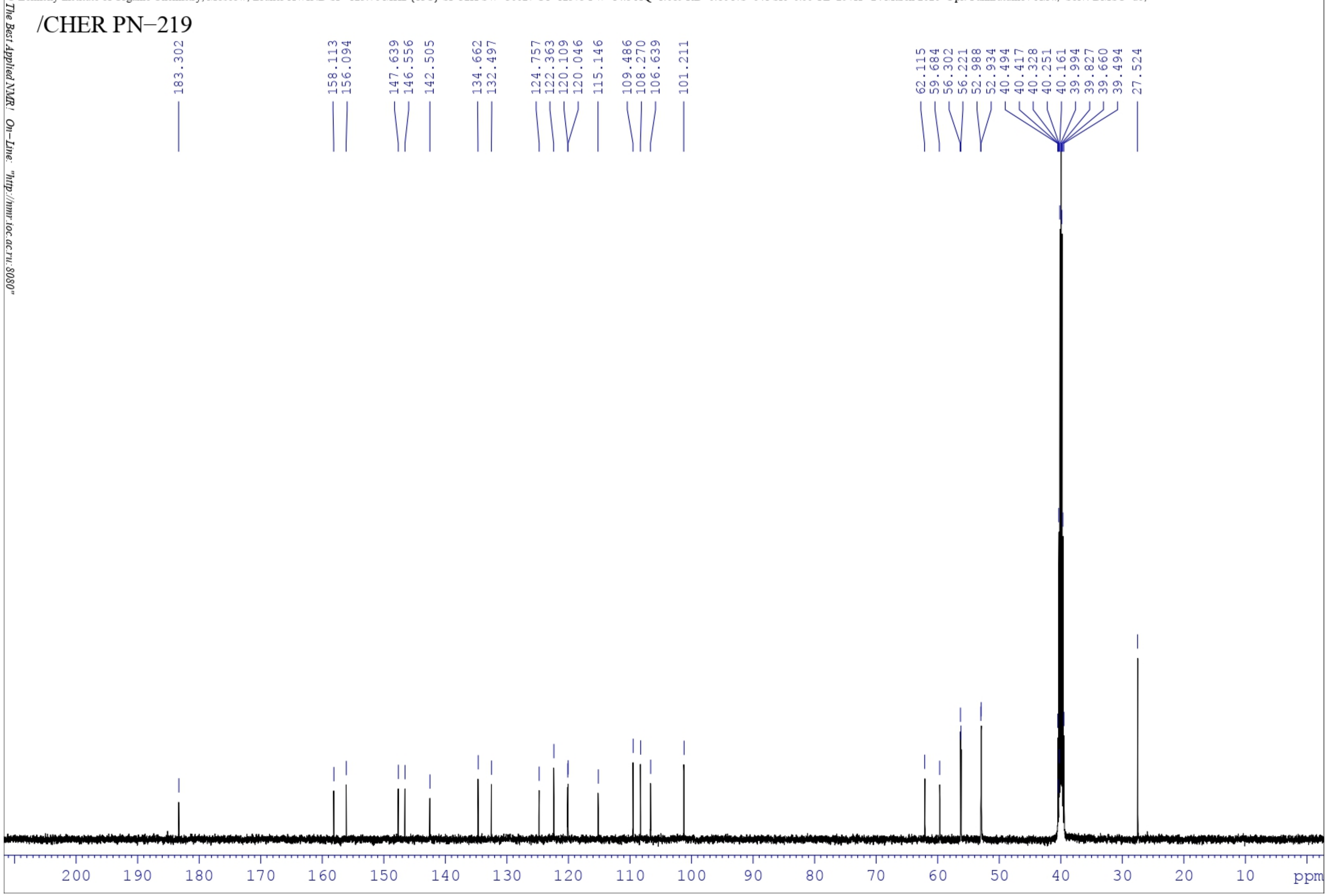

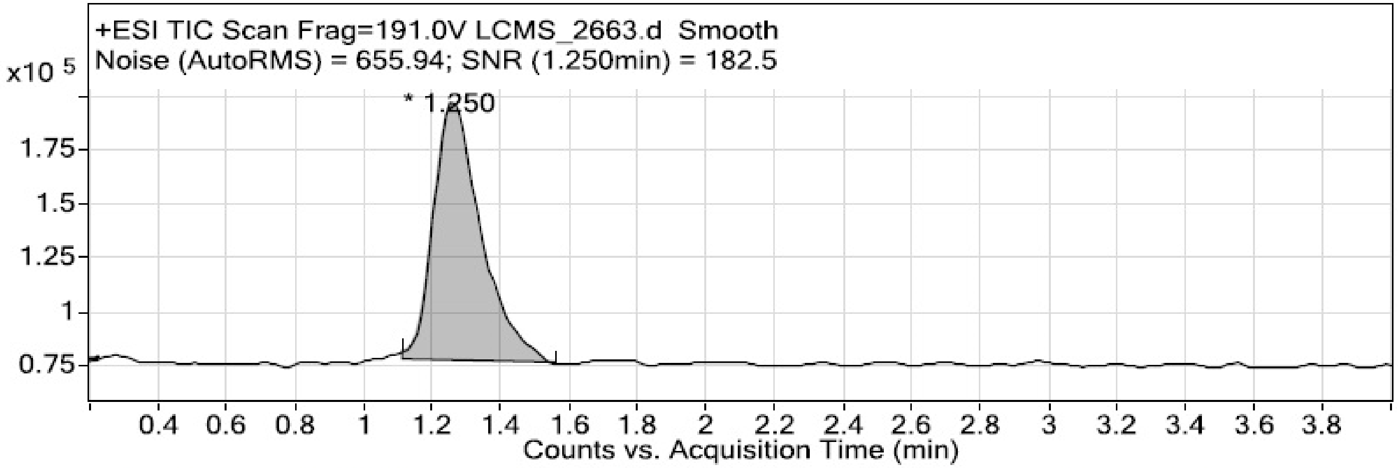
+ESI TIC Scan Frag=191.0V LCMS_2663.d Smooth
Noise (AutoRMS) = 655.94; SNR (1.250min) = 182.5
x10 5
* 1.250
1.75
1.5
1.25
1
0.75
0.4 0.6 0.8 1 1.2 1.4 1.6 1.8 2 2.2 2.4 2.6 2.8 3 3.2 3.4 3.6 3.8
Counts vs. Acquisition Time (min)

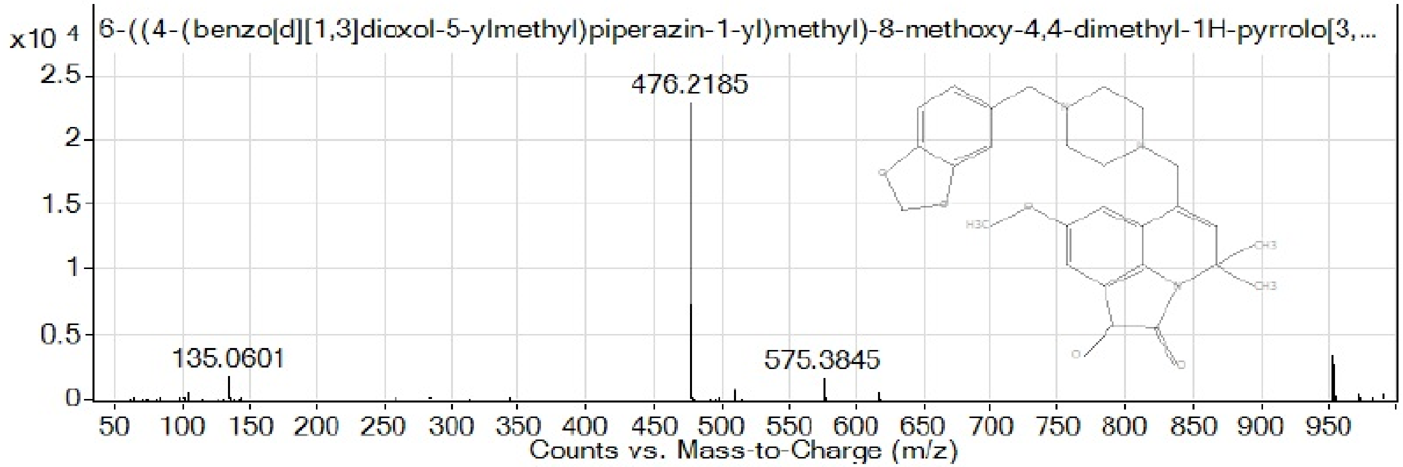
x10 4
6-((4-(benzo[d][1,3]dioxol-5-ylmethyl)piperazin-1-yl)methyl)-8-methoxy-4,4-dimethyl-1H-pyrrolo[3,...
2.5
2
1.5
1
0.5
0
476.2185
135.0601
575.3845
50 100 150 200 250 300 350 400 450 500 550 600 650 700 750 800 850 900 950
Counts vs. Mass-to-Charge (m/z)

6-((4-(4-fluorophenyl)piperazine-1-yl)methyl)-8-methoxy-4,4-dimethyl-1*H*-pyrrolo[3,2,1-*ij*]quinolin-1,2(4*H*)-dione 14e

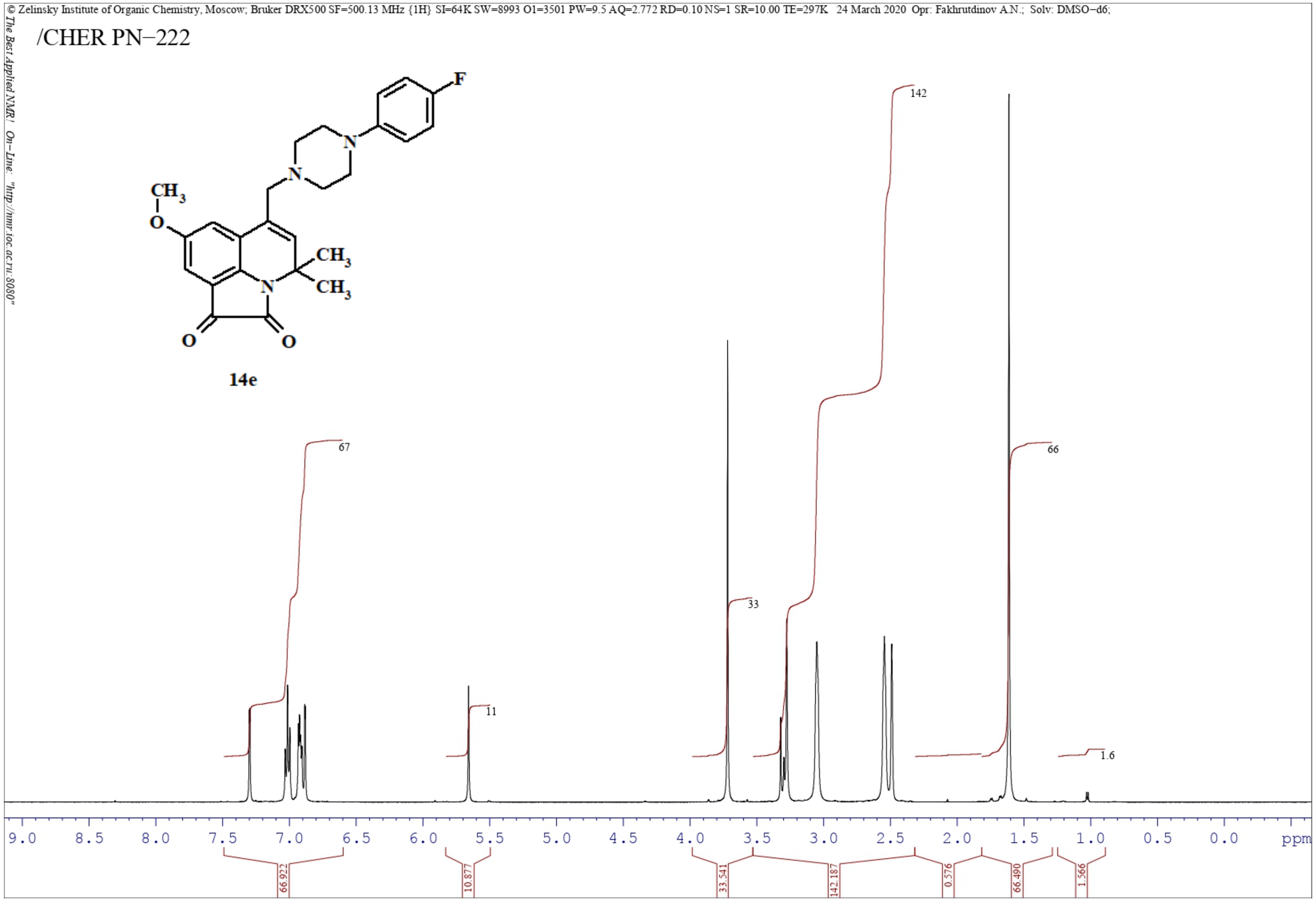

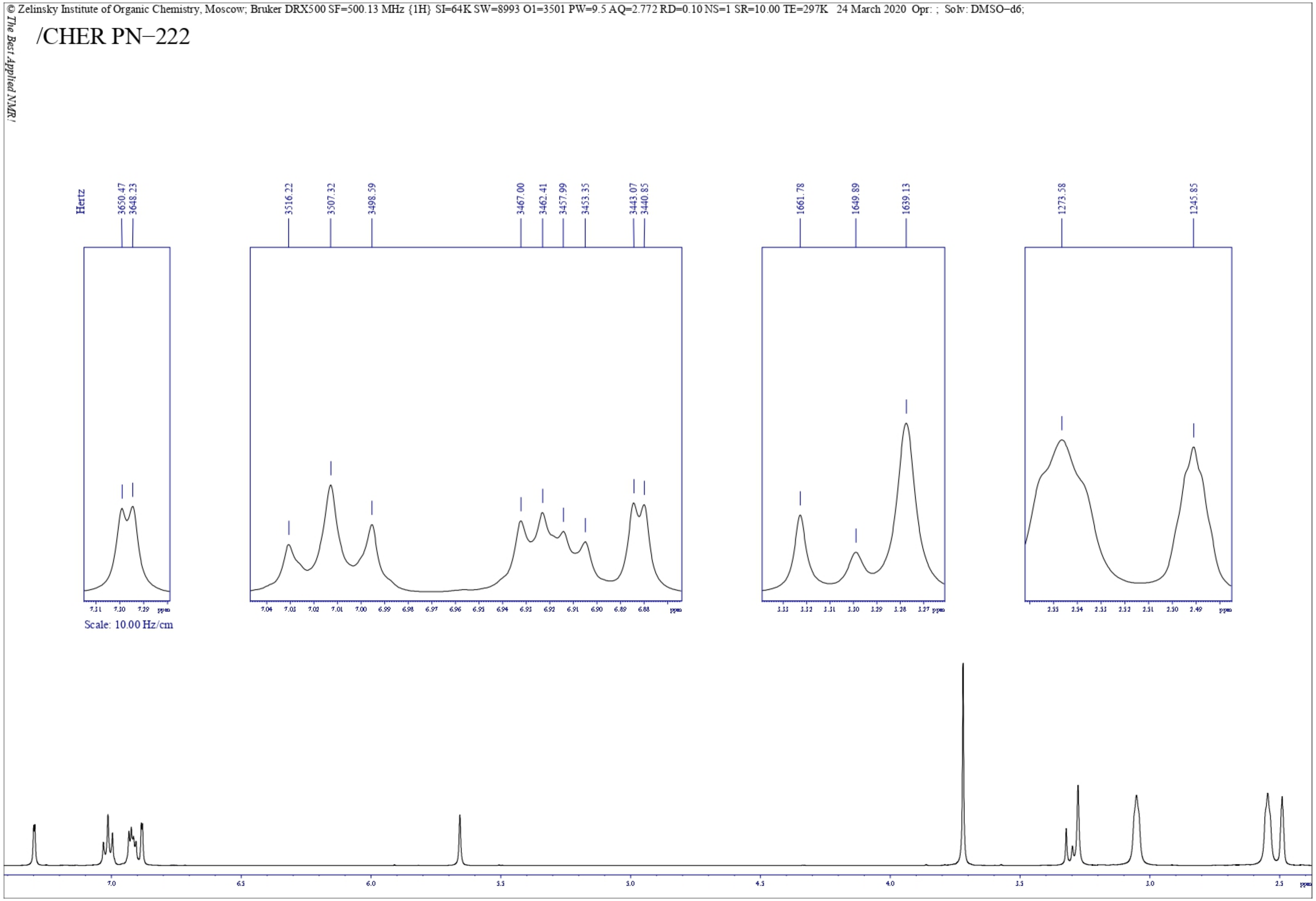

© Zelinsky Institute of Organic Chemistry, Moscow; Bruker DRX500 SF=500.13 MHz {1H} SI=64K SW=8993 O1=3501 PW=9.5 AQ=2.772 RD=0.10 NS=1 SR=10.00 TE=297K 24 March 2020 Opr: ; Solv: DMSO−d6;
The Best Applied NMR!
/CHER PN−222
Hertz
3650.47
3648.23
3516.22
3507.32
3498.59
3467.00
3462.41
3457.99
3453.35
3443.07
3440.85
1661.78
1649.89
1639.13
1273.58
1245.85
Scale: 10.00 Hz/cm
ppm

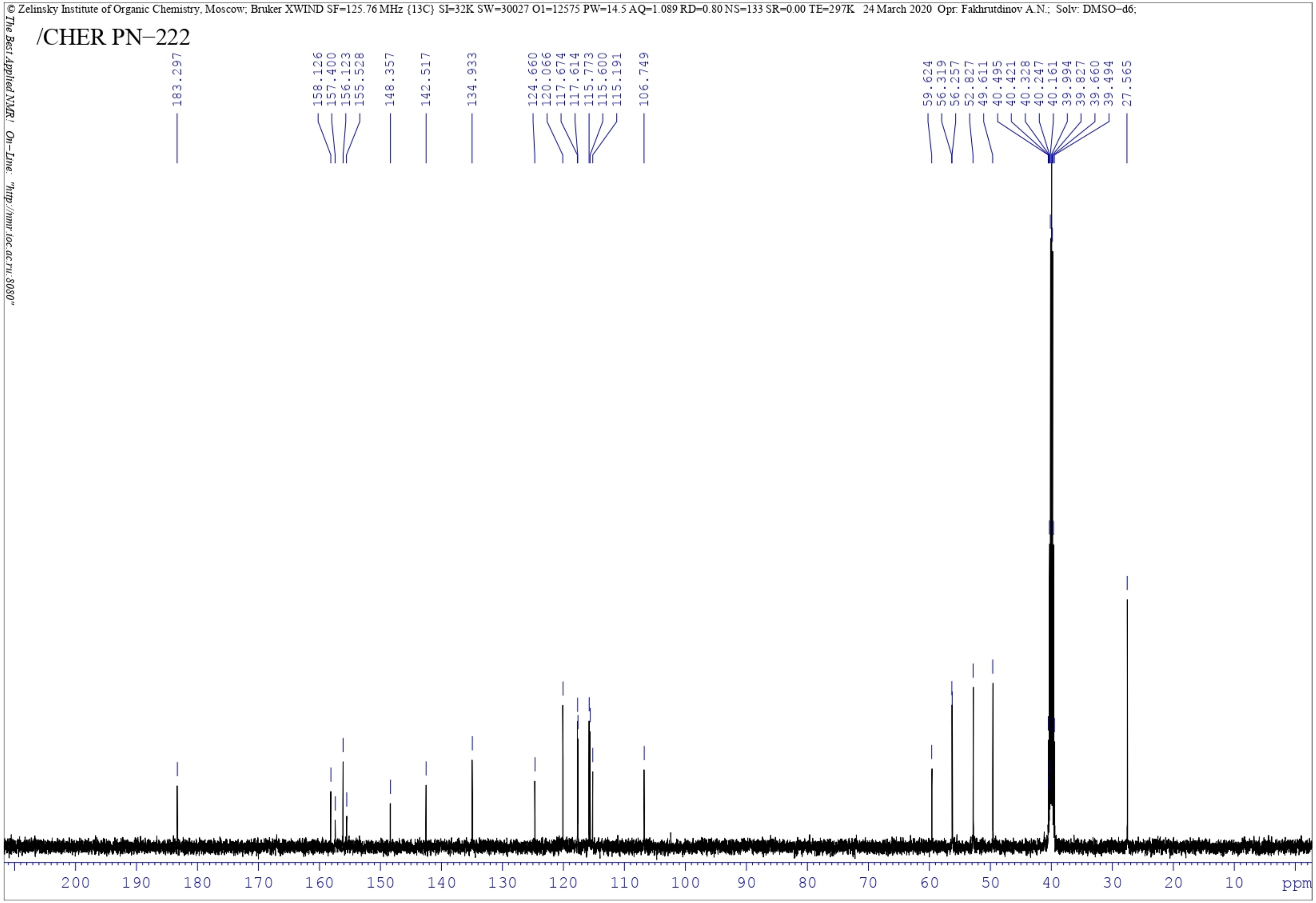
© Zelinsky Institute of Organic Chemistry, Moscow; Bruker XWIND SF=125.76 MHz {13C} SI=32K SW=30027 O1=12575 PW=14.5 AQ=1.089 RD=0.80 NS=133 SR=0.00 TE=297K 24 March 2020 Opr: Fakhrutdinov A.N.; Solv: DMSO−d6;
The Best Applied NMR! On−Line: "http://nmr.ioc.ac.ru:8080"
/CHER PN−222
183.297
158.126
157.400
156.123
155.528
148.357
142.517
134.933
124.660
120.066
117.674
117.614
115.773
115.600
115.191
106.749
59.624
56.319
56.257
52.827
49.611
40.495
40.421
40.328
40.247
40.161
39.994
39.827
39.660
39.494
27.565
200
190
180
170
160
150
140
130
120
110
100
90
80
70
60
50
40
30
20
10
ppm

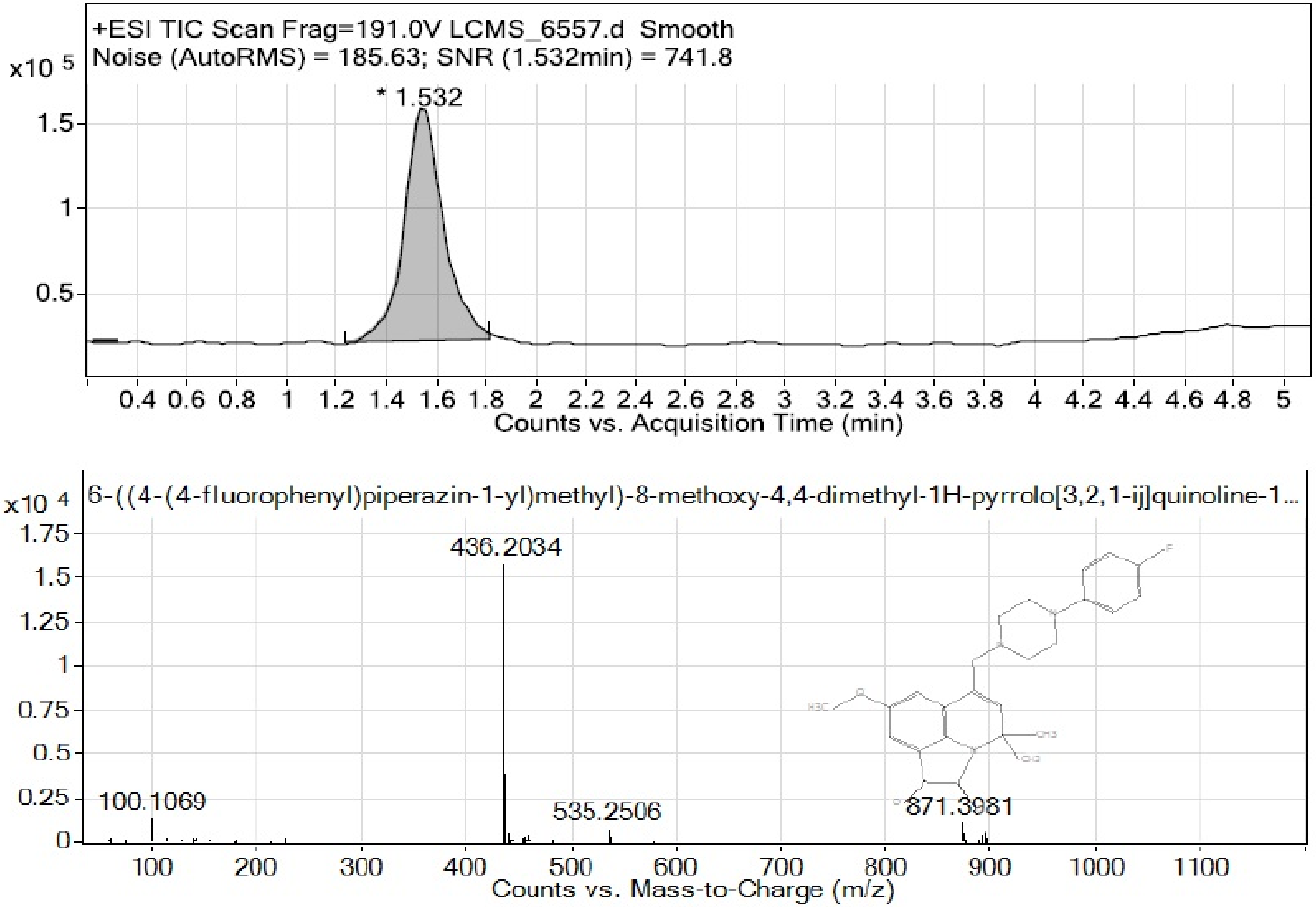
+ESI TIC Scan Frag=191.0V LCMS_6557.d Smooth
Noise (AutoRMS) = 185.63; SNR (1.532min) = 741.8
x10 5
* 1.532
Counts vs. Acquisition Time (min)
x10 4
6-((4-(4-fluorophenyl)piperazin-1-yl)methyl)-8-methoxy-4,4-dimethyl-1H-pyrrolo[3,2,1-ij]quinoline-1...
436.2034
100.1069
535.2506
871.3981
Counts vs. Mass-to-Charge (m/z)

6-((4-benzo[*d*][1,3]dioxol-5-ylmethyl)piperazine-1-yl)methyl)-8-fluoro-4,4-dimethyl-1*H*-pyrrolo[3,2,1-*ij*]quinolin-1,2(4*H*)-dione

14f

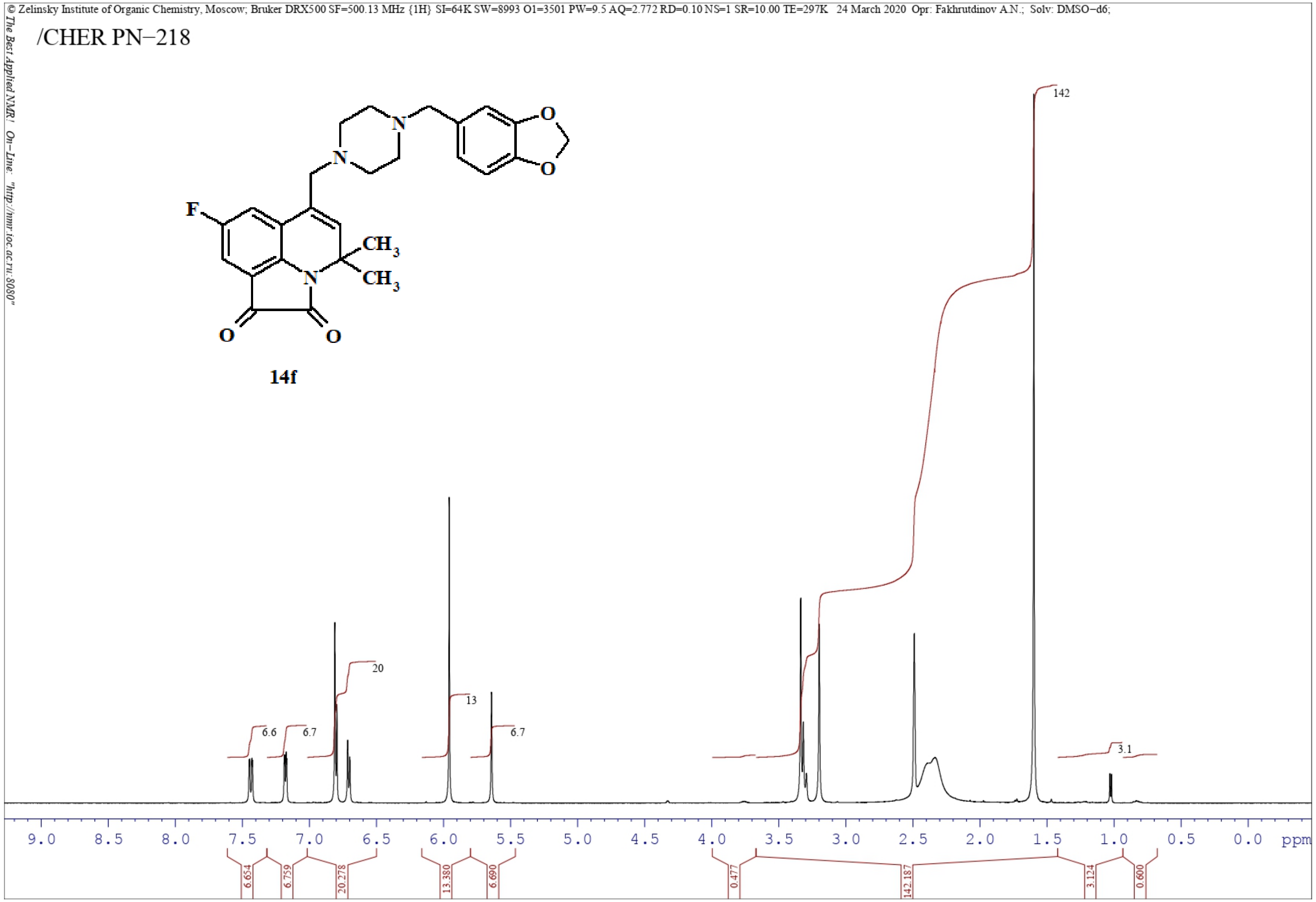

© Zelinsky Institute of Organic Chemistry, Moscow; Bruker DRX500 SF=500.13 MHz {1H} SI=64K SW=8993 O1=3501 PW=9.5 AQ=2.772 RD=0.10 NS=1 SR=10.00 TE=297K 24 March 2020 Opr: ; Solv: DMSO−d6;

The Best Applied NMR!

/CHER PN−218

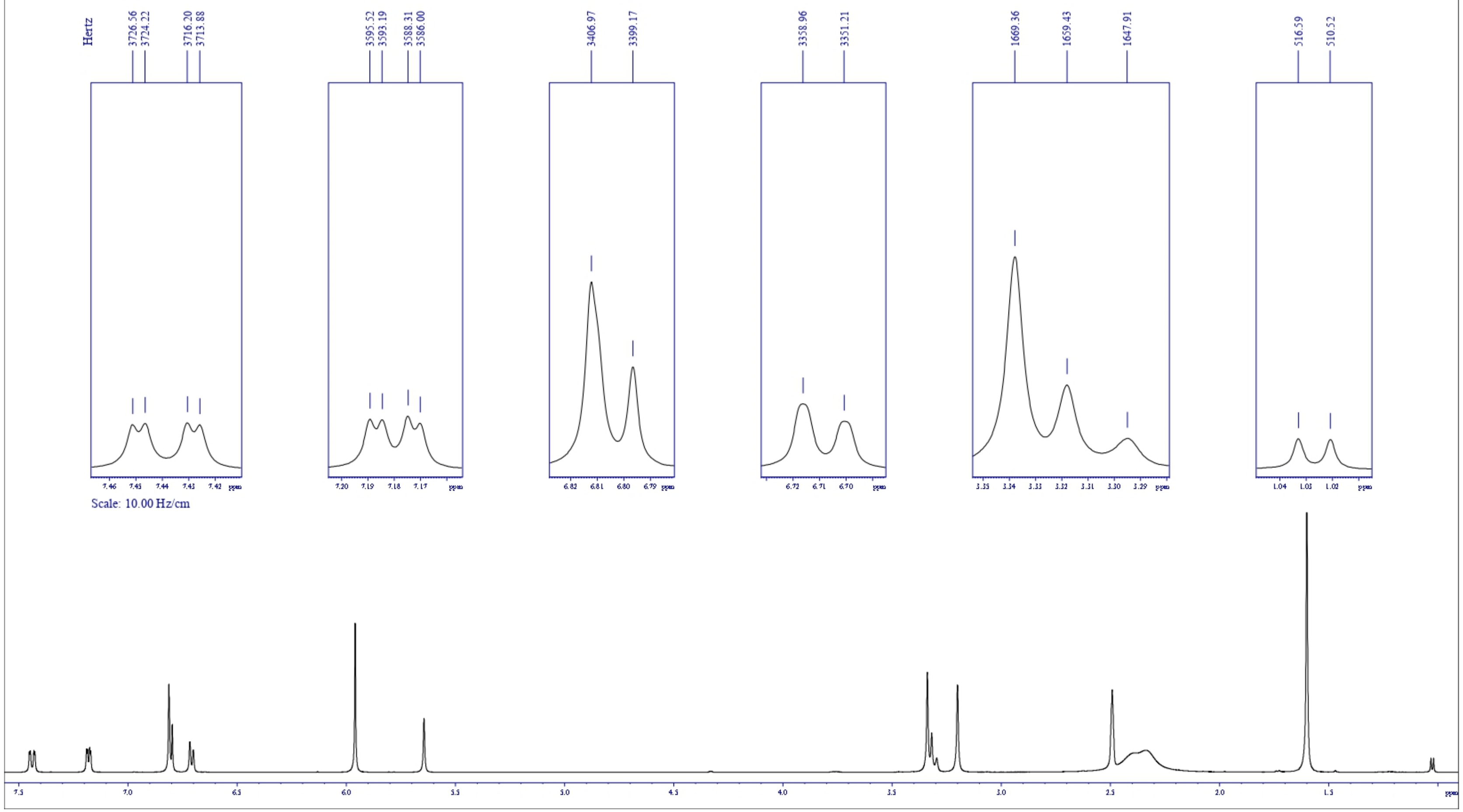

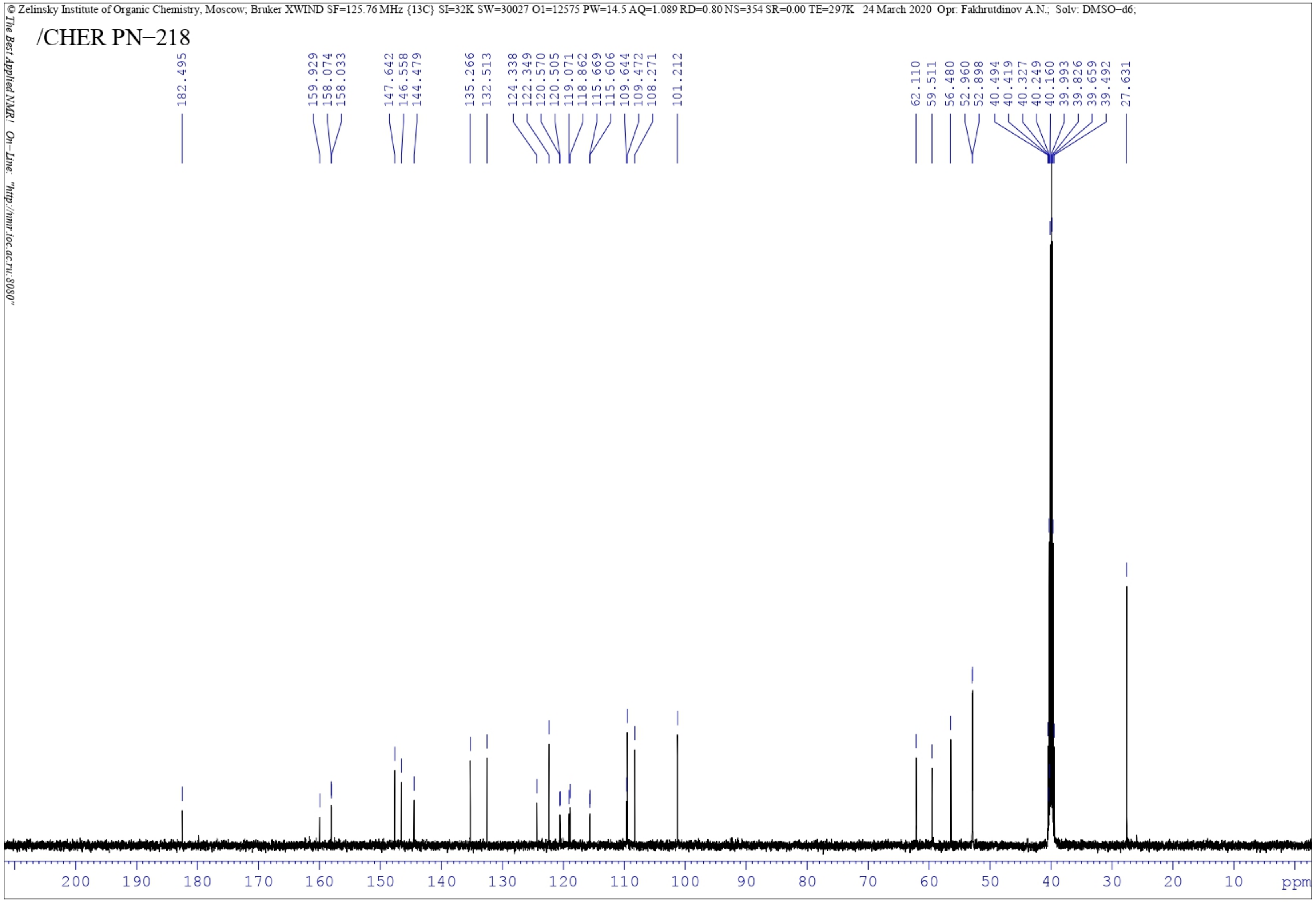

© Zelinsky Institute of Organic Chemistry, Moscow; Bruker XWIND SF=125.76 MHz {13C} SI=32K SW=30027 O1=12575 PW=14.5 AQ=1.089 RD=0.80 NS=354 SR=0.00 TE=297K 24 March 2020 Opr. Fakhrutdinov A.N.; Solv: DMSO-d6;
The Best Applied NMR! On-Line: "http://nmr.ioc.ac.ru:8080"
/CHER PN-218
182.495
159.929
158.074
158.033
147.642
146.558
144.479
135.266
132.513
124.338
122.349
120.570
120.505
119.071
118.862
115.669
115.606
109.644
109.472
108.271
101.212
62.110
59.511
56.480
52.960
52.898
40.494
40.419
40.327
40.249
40.160
39.993
39.826
39.659
39.492
27.631
200 190 180 170 160 150 140 130 120 110 100 90 80 70 60 50 40 30 20 10 ppm

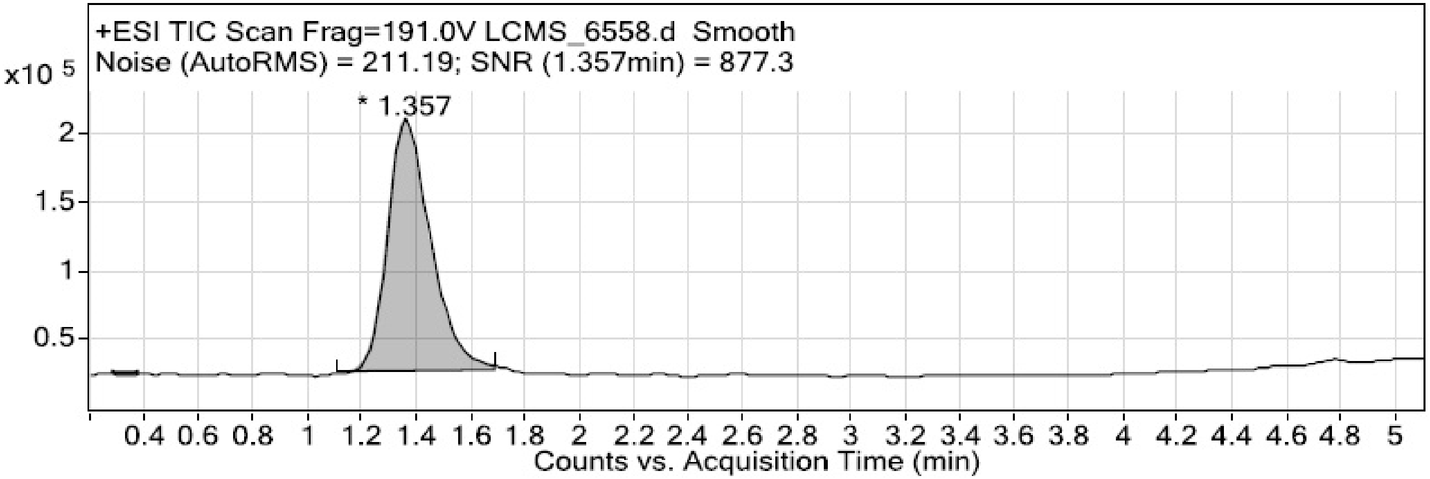
+ESI TIC Scan Frag=191.0V LCMS_6558.d Smooth
Noise (AutoRMS) = 211.19; SNR (1.357min) = 877.3
x10 5
* 1.357
Counts vs. Acquisition Time (min)

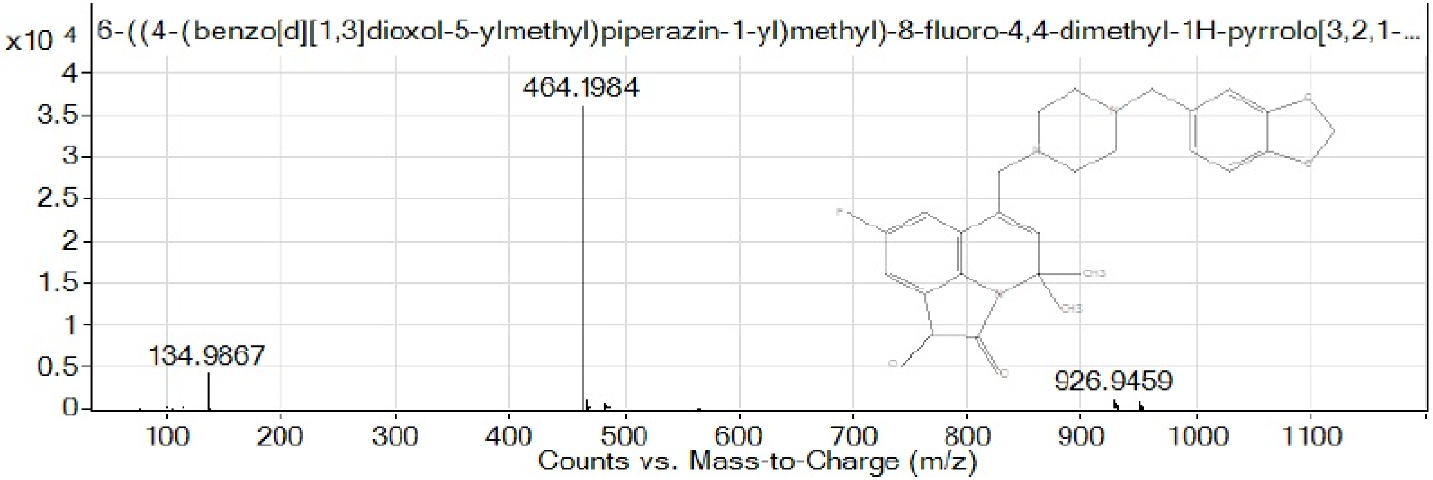
x10 4
6-((4-(benzo[d][1,3]dioxol-5-ylmethyl)piperazin-1-yl)methyl)-8-fluoro-4,4-dimethyl-1H-pyrrolo[3,2,1-...
464.1984
134.9867
926.9459
Counts vs. Mass-to-Charge (m/z)

^{1}H, ^{13}C NMR spectra and data of HPLC-MS-ESI analysis of PQ 9

(*Z*)-Ethyl 4-((8-methoxy-4,4-dimethyl-2-oxo-1-(4-oxo-2-thioxothiazolidin-5-ylidene)-2,4-dihydro-1*H*-pyrrolo[3,2,1-*ij*]quinolin-6-yl)methyl)piperazine-1-carboxylate 9a

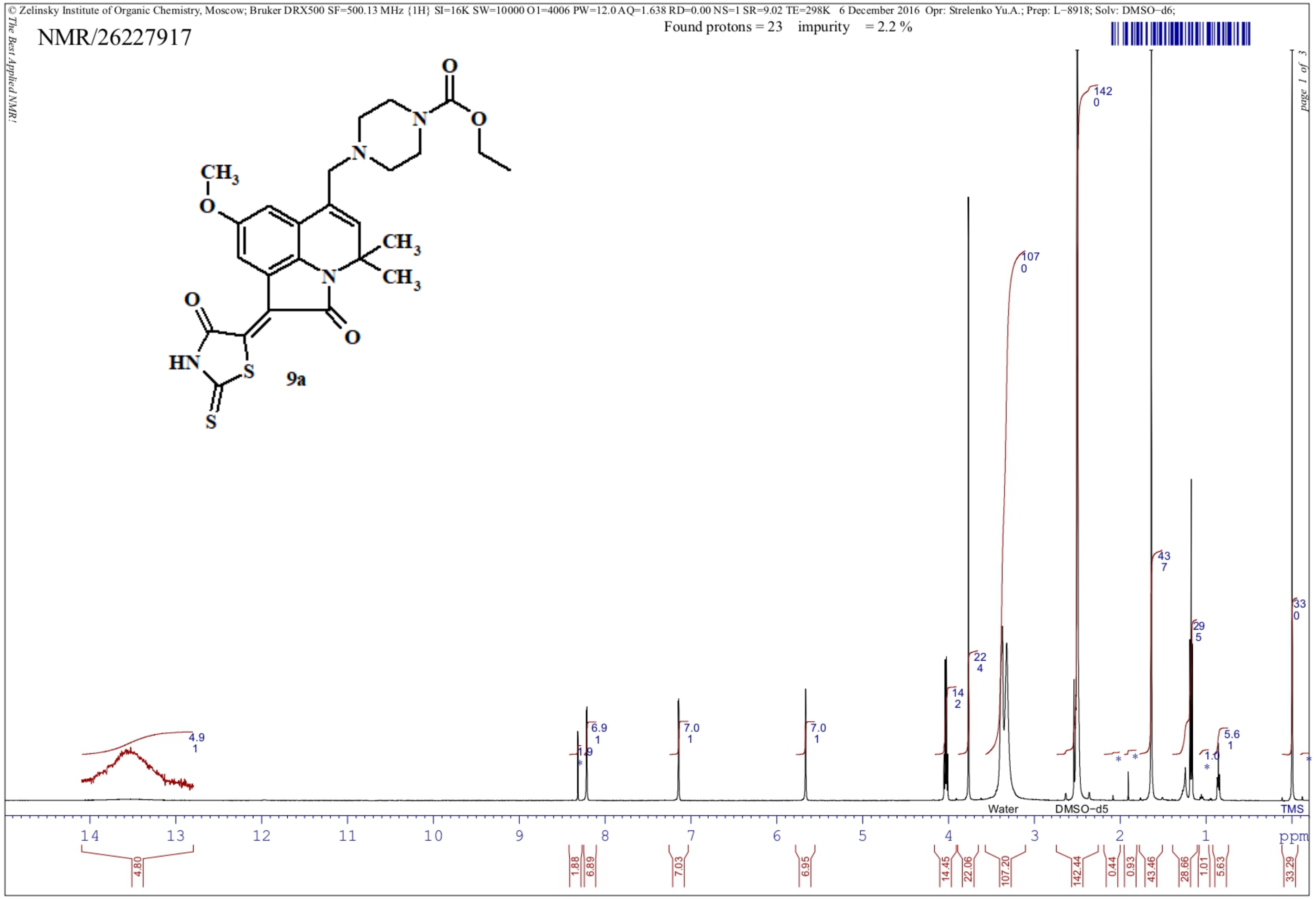

© Zelinsky Institute of Organic Chemistry, Moscow; Bruker DRX500 SF=500.13 MHz {1H} SI=16K SW=10000 O1=4006 PW=12.0 AQ=1.638 RD=0.00 NS=1 SR=9.02 TE=298K 6 December 2016 Opr: Strelenko Yu.A.; Prep: L−8918; Solv: DMSO−d6;

The Best Applied NMR!

NMR/26227917

page 2 of 3

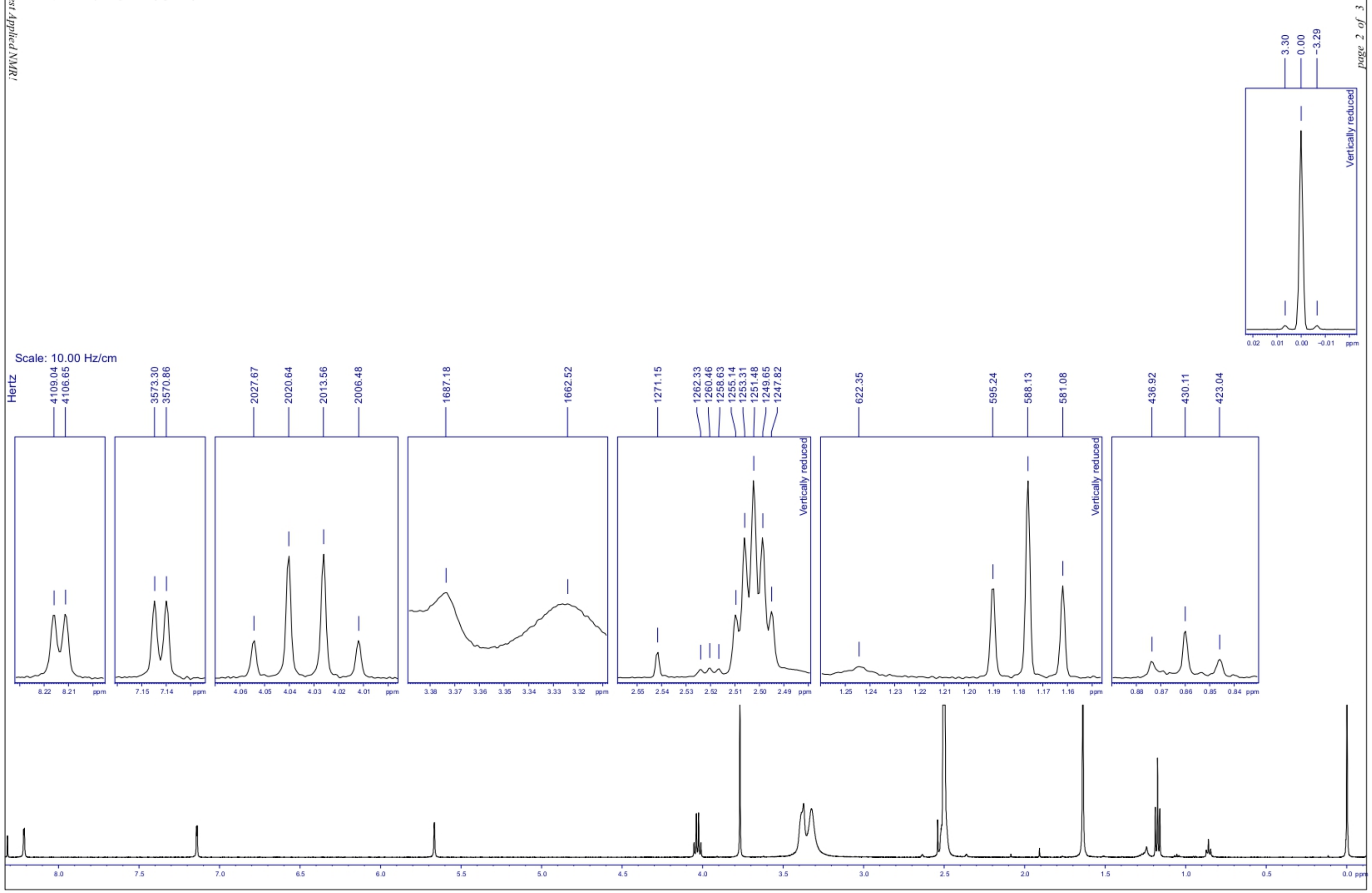

© Zelinsky Institute of Organic Chemistry, Moscow; Bruker DRX500 SF=125.76 MHz {13C} SI=64K SW=31443 O1=13259 PW=8.0 AQ=0.521 RD=1.00 NS=381 SR=72.96 TE=299K 18 February 2020 Opr: Strelenko Yu.A.; Prep: L−9604; Solv: DMSO−d6;

The Best Applied NMR!

NMR/29317389

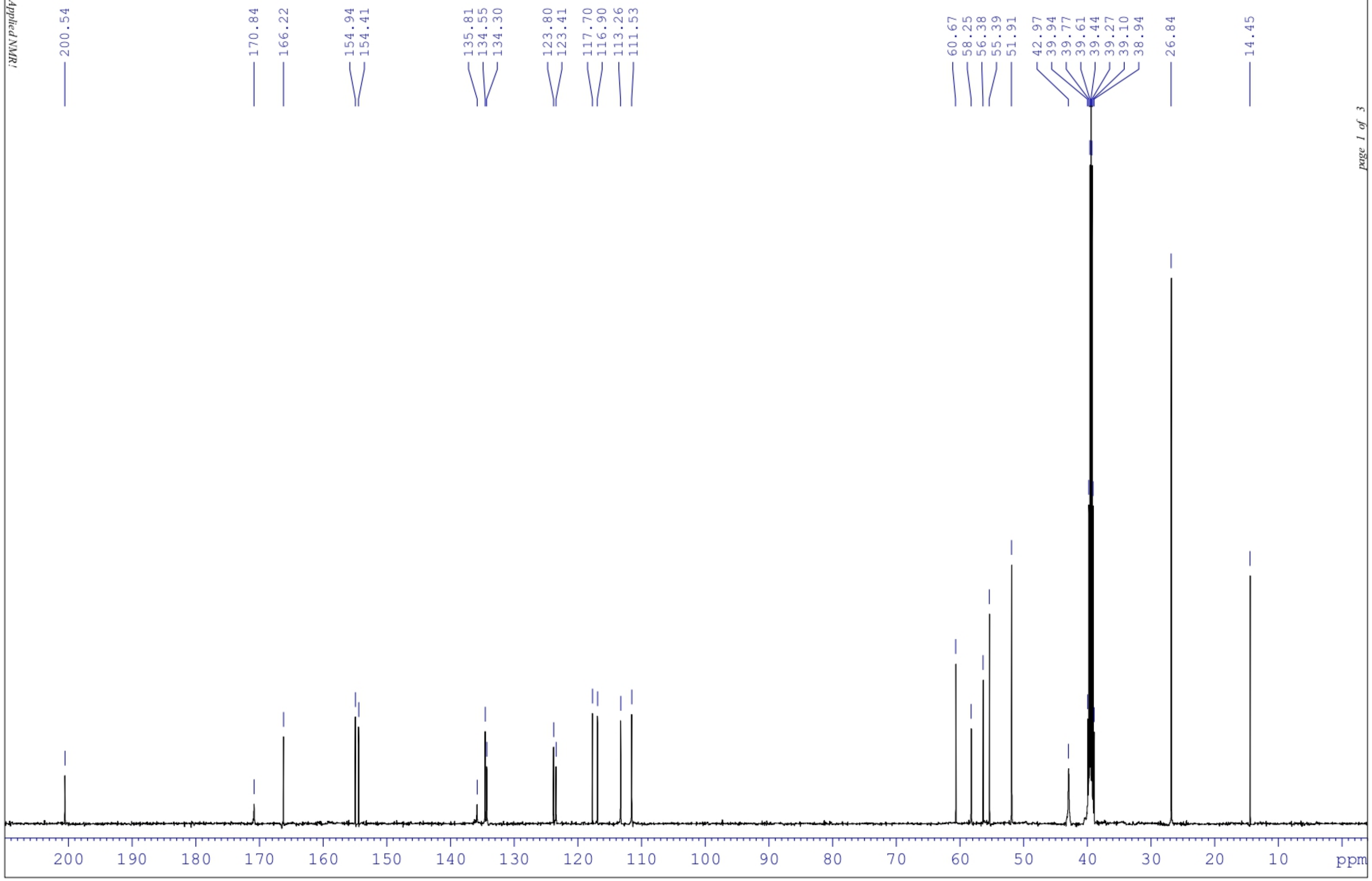

page 1 of 3

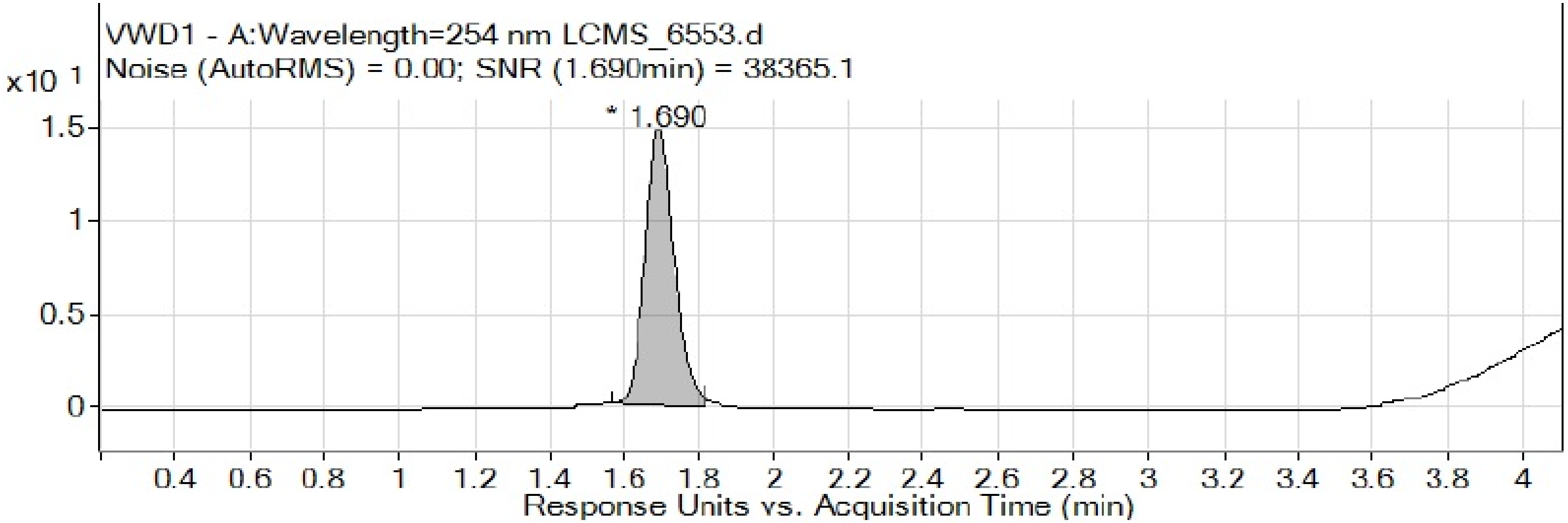
VWD1 - A:Wavelength=254 nm LCMS_6553.d
Noise (AutoRMS) = 0.00; SNR (1.690min) = 38365.1
x10 1
* 1.690
Response Units vs. Acquisition Time (min)

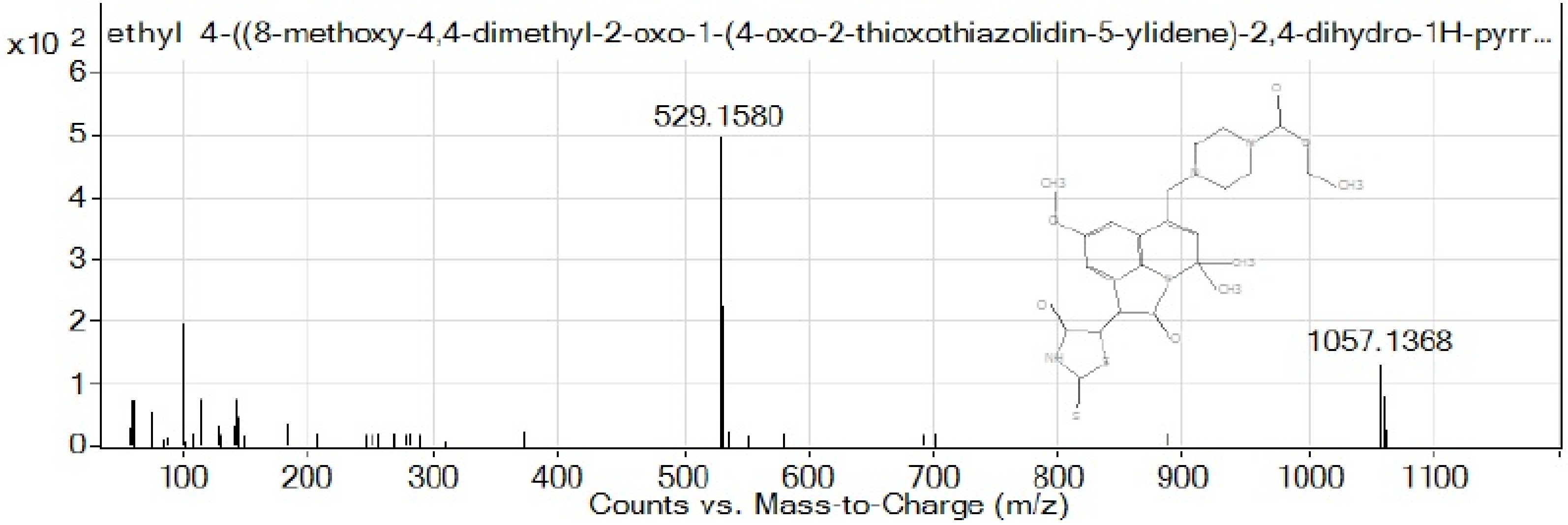
ethyl 4-((8-methoxy-4,4-dimethyl-2-oxo-1-(4-oxo-2-thioxothiazolidin-5-ylidene)-2,4-dihydro-1H-pyrr...
x10 2
529.1580
1057.1368
Counts vs. Mass-to-Charge (m/z)

(*Z*)-5-(8-methoxy-4,4-dimethyl-2-oxo-6-((4-phenylpiperazin-1-yl)methyl)-2,4-dihydro-1*H*-pyrrolo[3,2,1-*ij*]quinolin-1-ylidene)-2-thioxothiazolidin-4-one 9b

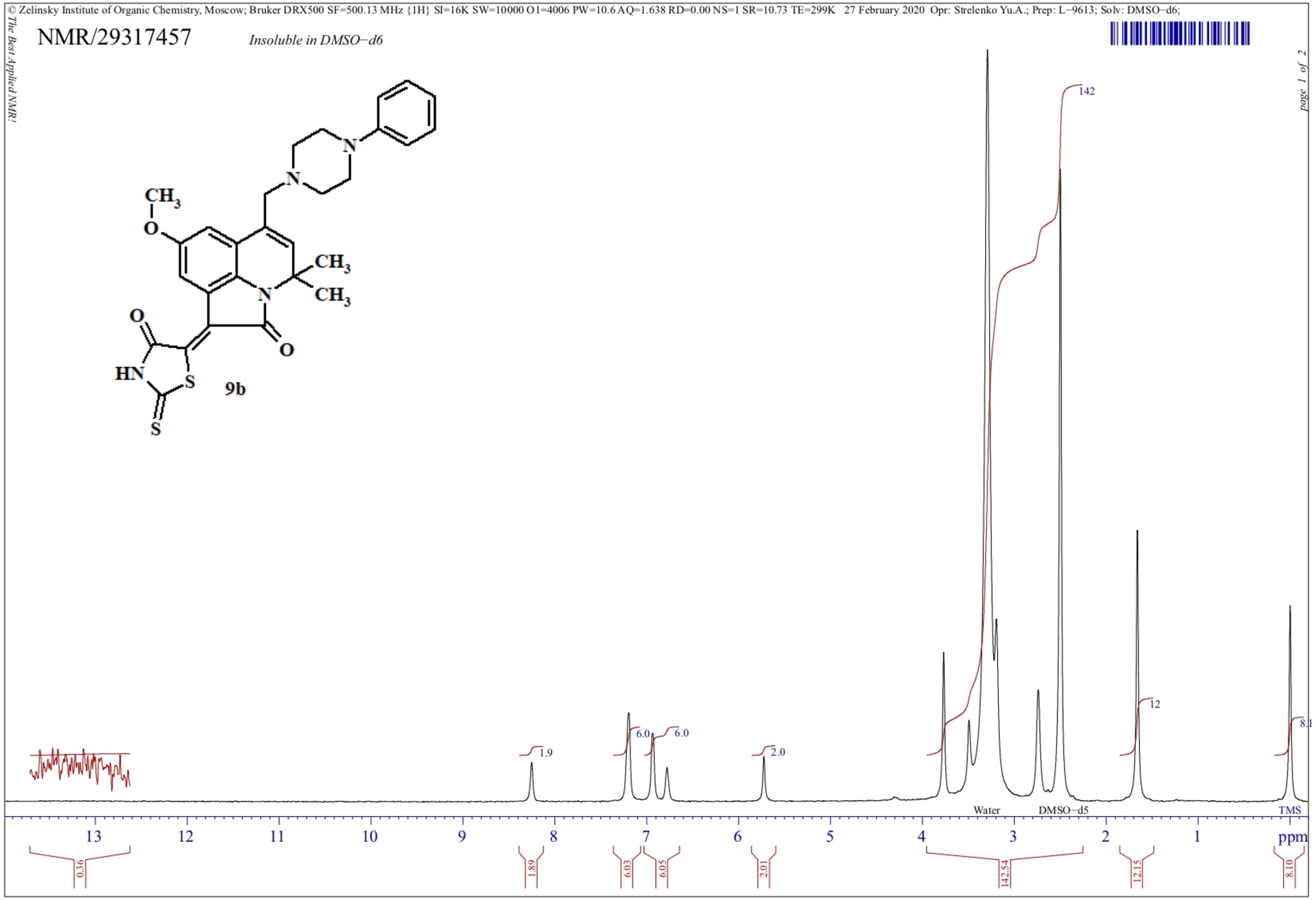

© Zelinsky Institute of Organic Chemistry, Moscow; Bruker DRX500 SF=125.76 MHz {13C} SI=64K SW=31443 O1=13259 PW=8.0 AQ=0.521 RD=1.00 NS=580 SR=72.11 TE=353K 3 March 2020 Opr: Strelenko Yu.A.; Solv: DMSO−d6;

The Best Applied NMR!

NMR/29317457

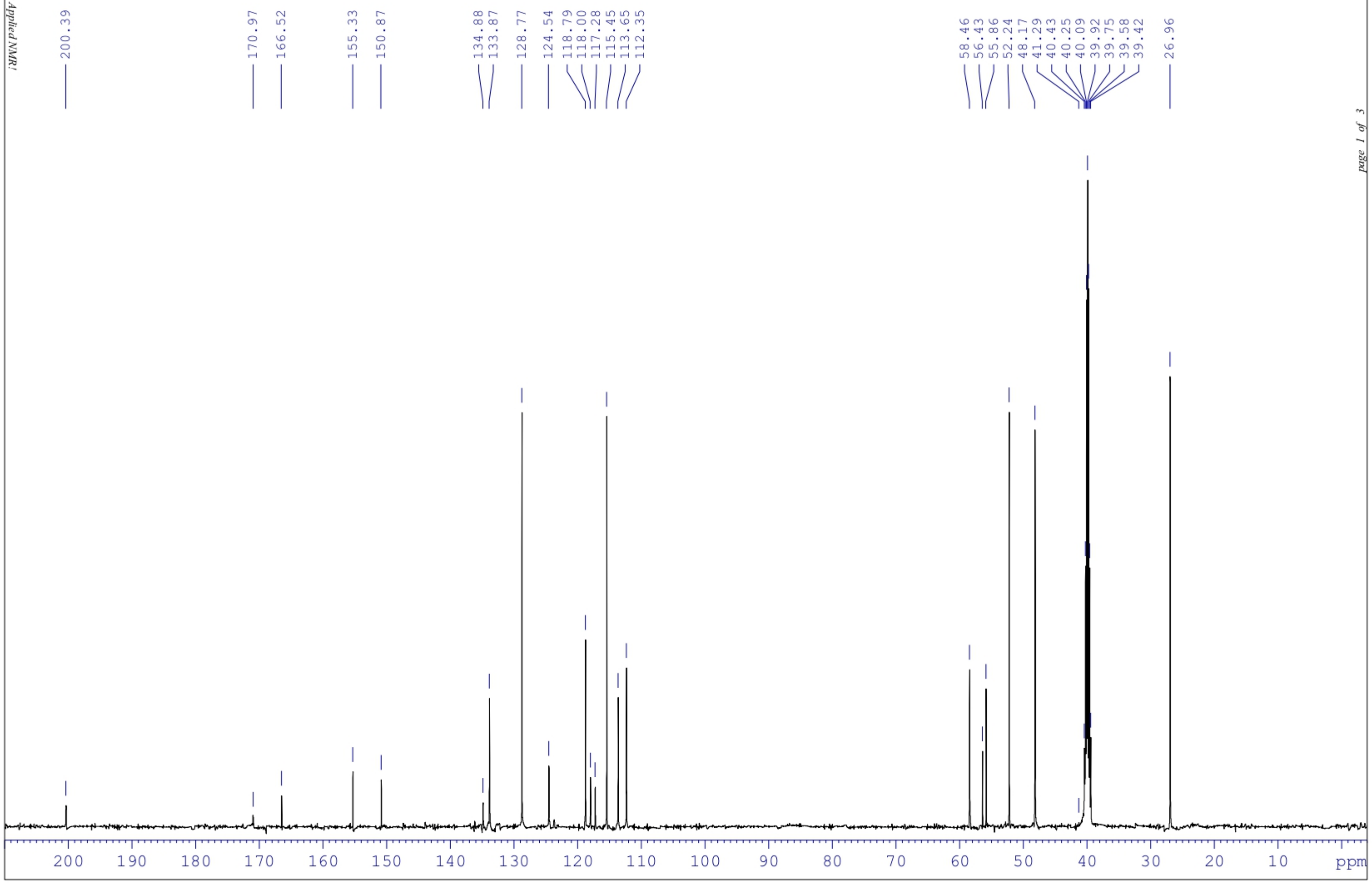

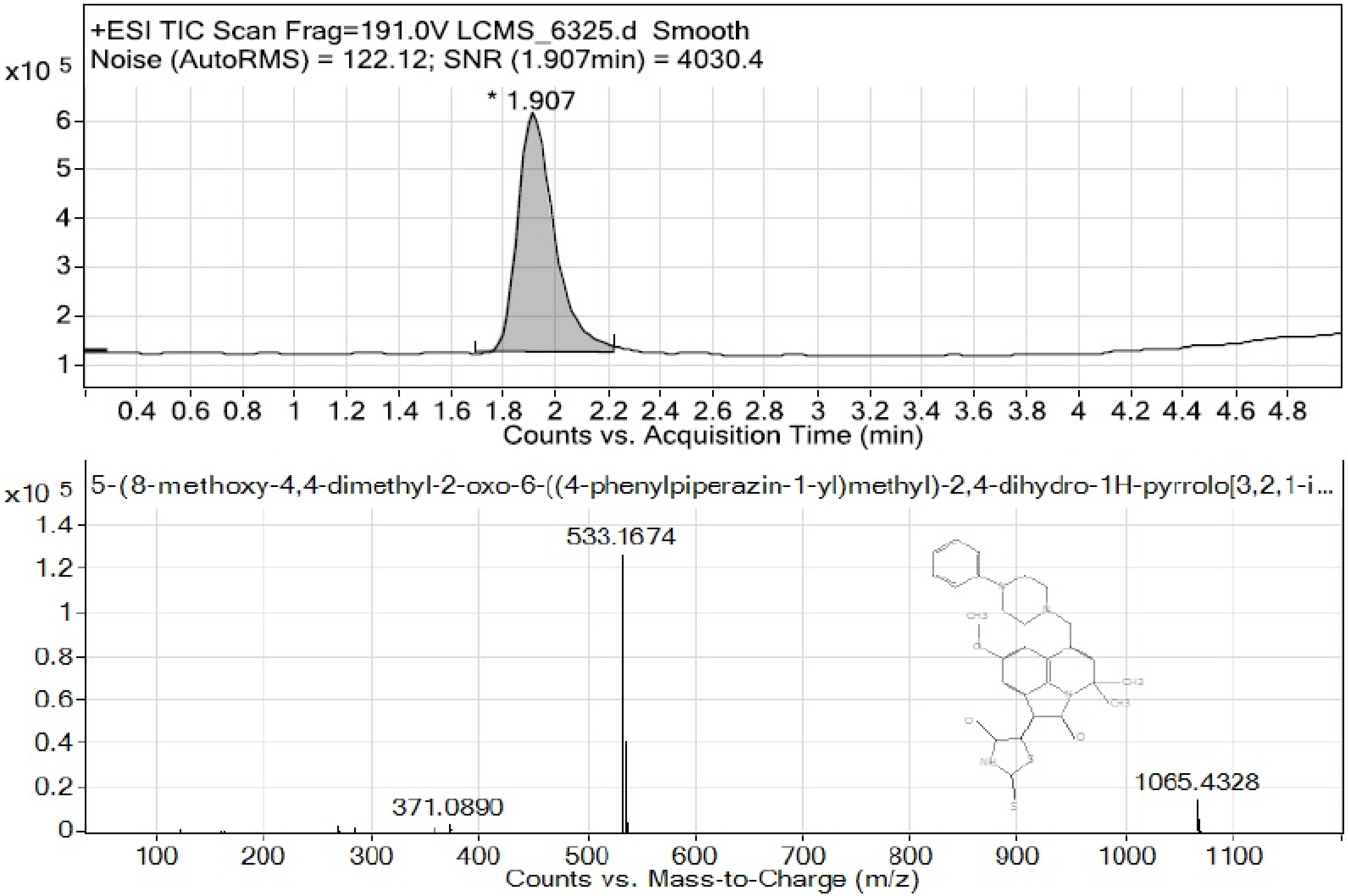
+ESI TIC Scan Frag=191.0V LCMS_6325.d Smooth
Noise (AutoRMS) = 122.12; SNR (1.907min) = 4030.4
x10 5
* 1.907
Counts vs. Acquisition Time (min)
5-(8-methoxy-4,4-dimethyl-2-oxo-6-((4-phenylpiperazin-1-yl)methyl)-2,4-dihydro-1H-pyrrolo[3,2,1-i...
533.1674
371.0890
1065.4328
Counts vs. Mass-to-Charge (m/z)

(Z)-Ethyl 4-((8-fluoro-4,4-dimethyl-2-oxo-1-(4-oxo-2-thioxothiazolidin-5-ylidene)-2,4-dihydro-1*H*-pyrrolo[3,2,1-*ij*]quinolin-6-yl)methyl)piperazine-1-carboxylate 9c

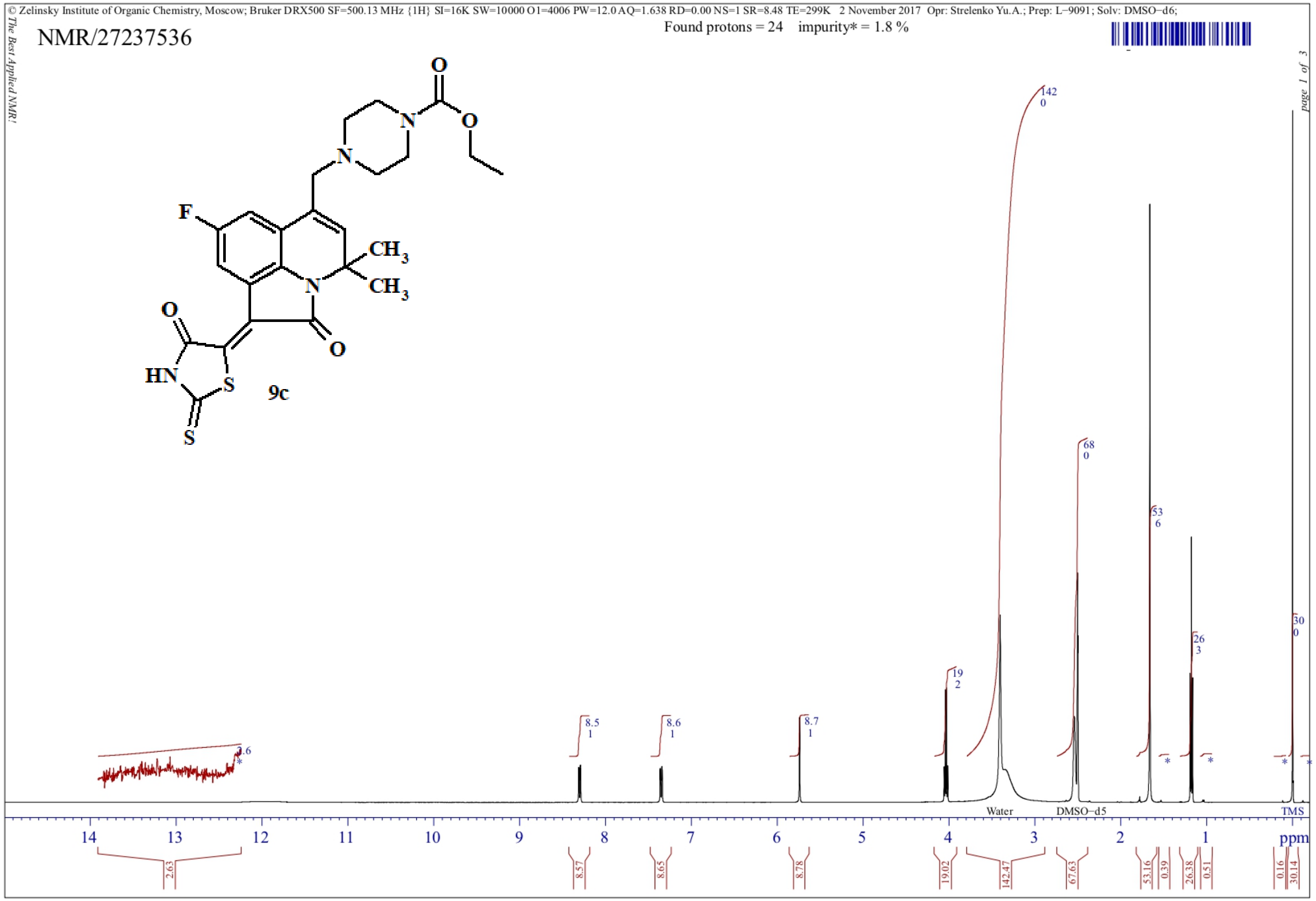

© Zelinsky Institute of Organic Chemistry, Moscow; Bruker DRX500 SF=500.13 MHz {1H} SI=16K SW=10000 O1=4006 PW=12.0 AQ=1.638 RD=0.00 NS=1 SR=8.48 TE=299K 2 November 2017 Opr: Strelenko Yu.A.; Prep: L−9091; Solv: DMSO−d6;

The Best Applied NMR!

NMR/27237536

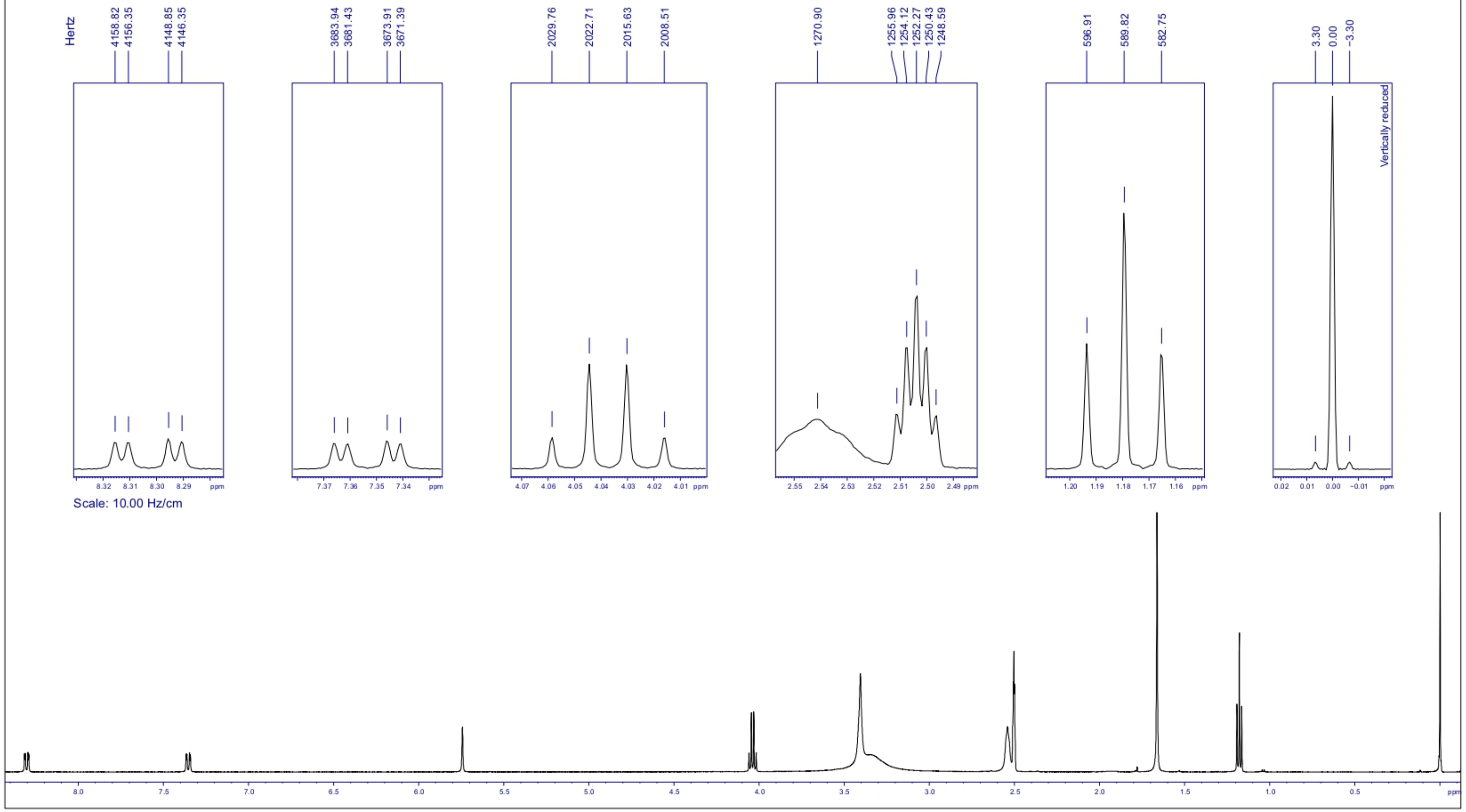

© Zelinsky Institute of Organic Chemistry, Moscow; Bruker DRX500 SF=125.76 MHz {13C} SI=64K SW=31443 O1=13259 PW=8.0 AQ=0.521 RD=1.00 NS=424 SR=72.96 TE=298K 13 February 2020 Opr: Strelenko Yu.A.; Prep: L−9601; Solv: DMSO−d6;

NMR/29317395

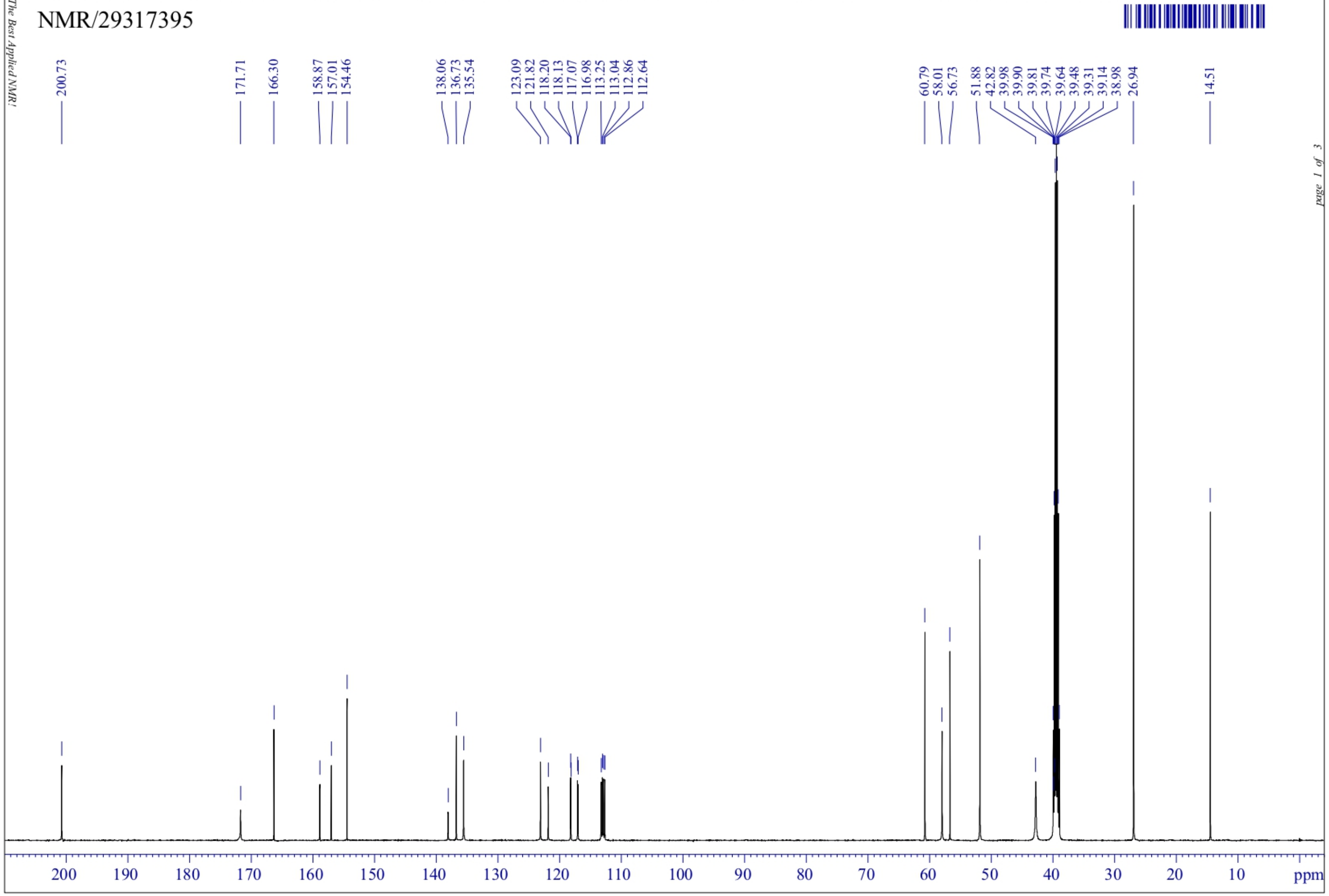

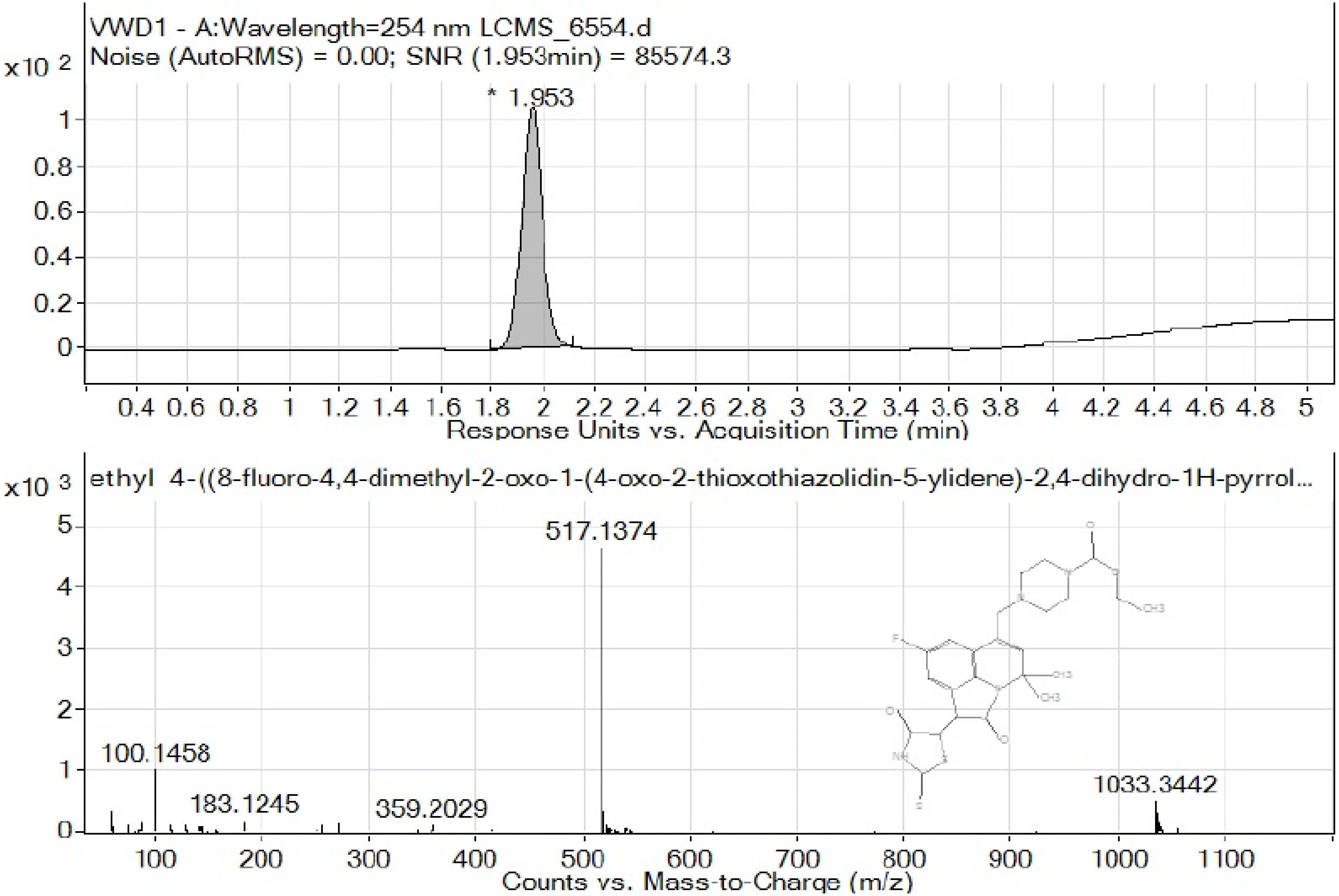
VWD1 - A:Wavelength=254 nm LCMS_6554.d
Noise (AutoRMS) = 0.00; SNR (1.953min) = 85574.3
x10 2
* 1.953
Response Units vs. Acquisition Time (min)
x10 3
ethyl 4-((8-fluoro-4,4-dimethyl-2-oxo-1-(4-oxo-2-thioxothiazolidin-5-ylidene)-2,4-dihydro-1H-pyrrol...
517.1374
100.1458
183.1245
359.2029
1033.3442
Counts vs. Mass-to-Charge (m/z)

(Z)-5-(6-((4-(benzo[*d*][1,3]dioxol-5-ylmethyl)piperazin-1-yl)methyl)-8-methoxy-4,4-dimethyl-2-oxo-2,4-dihydro-1*H*-pyrrolo[3,2,1-*ij*]quinolin-1-ylidene)-2-thioxothiazolidin-4-one 9d

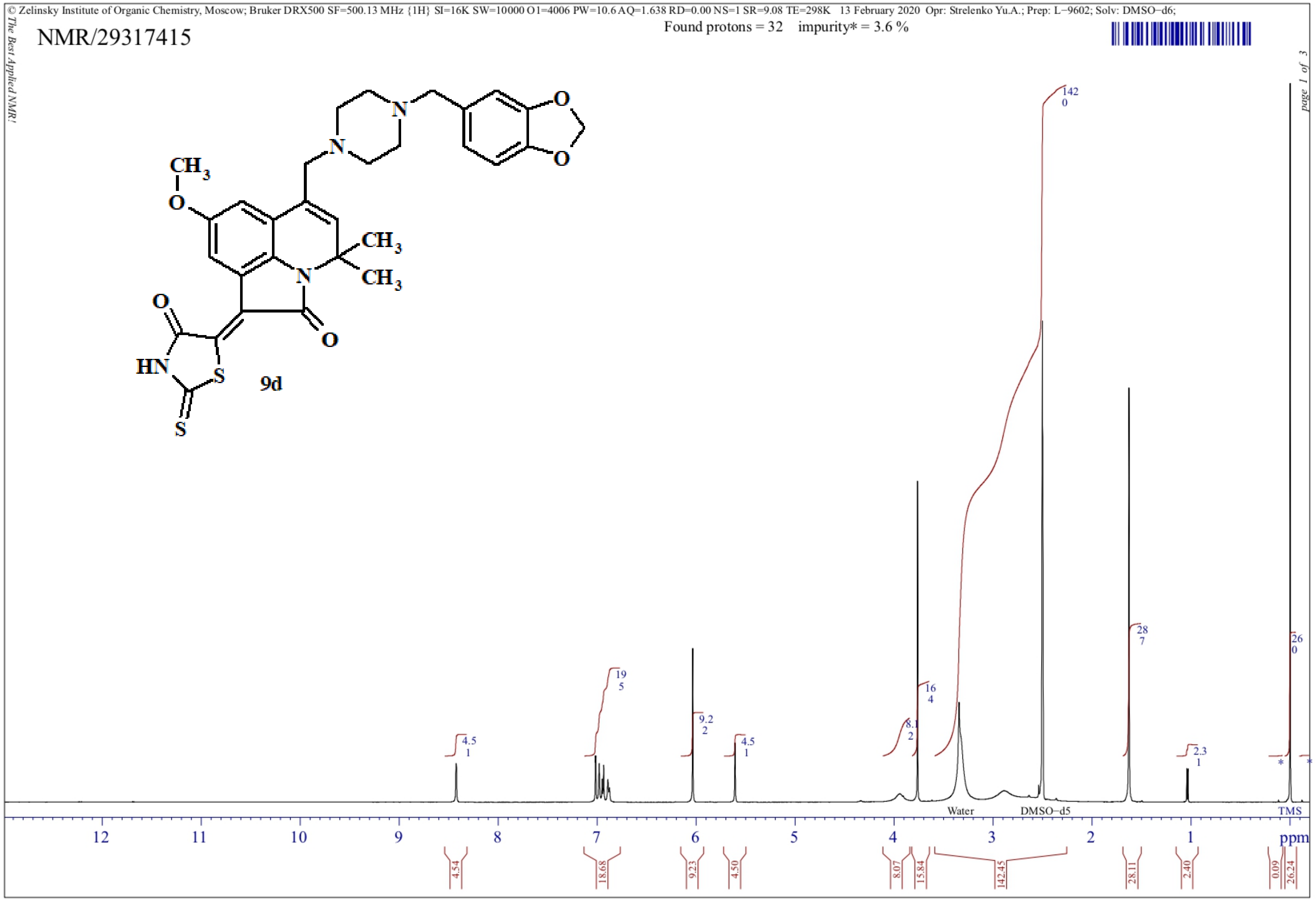

© Zelinsky Institute of Organic Chemistry, Moscow; Bruker DRX500 SF=500.13 MHz {1H} SI=16K SW=10000 O1=4006 PW=10.6 AQ=1.638 RD=0.00 NS=1 SR=9.08 TE=298K 13 February 2020 Opr: Strelenko Yu.A.; Prep: L−9602; Solv: DMSO−d6;

NMR/29317415

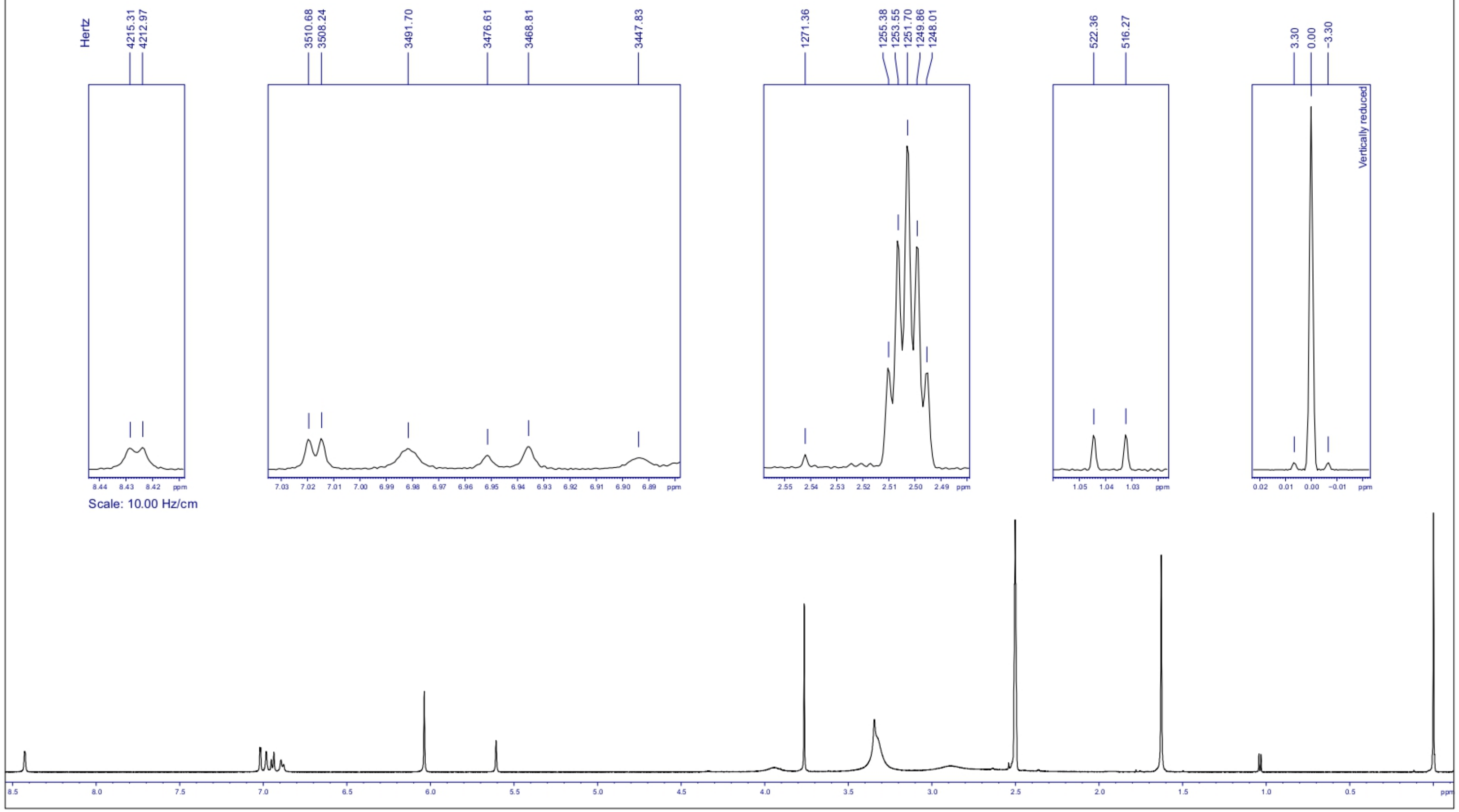

© Zelinsky Institute of Organic Chemistry, Moscow; Bruker DRX500 SF=125.76 MHz {13C} SI=64K SW=31443 O1=13259 PW=8.0 AQ=0.521 RD=1.00 NS=728 SR=72.96 TE=299K 18 February 2020 Opr: Strelenko Yu.A.; Prep: L−9604; Solv: DMSO−d6;

NMR/29317415

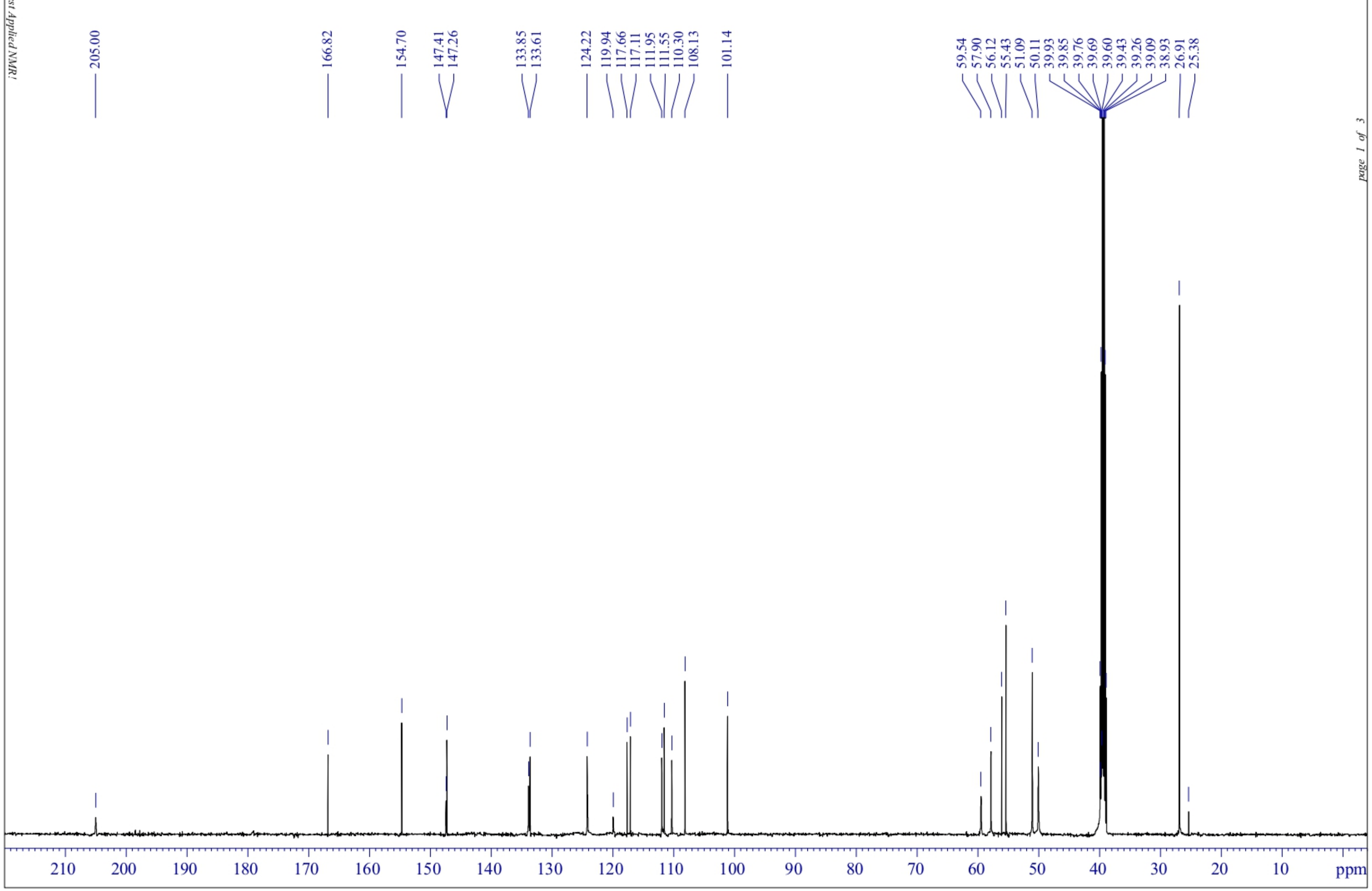

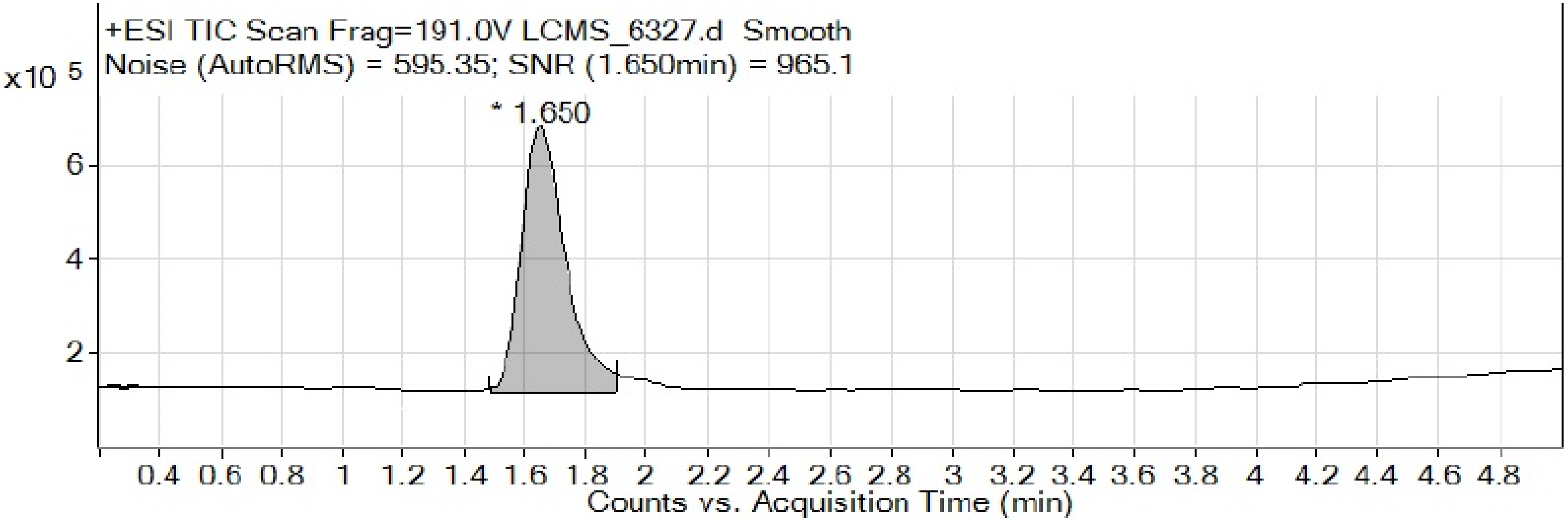

+ESI TIC Scan Frag=191.0V LCMS_6327.d Smooth
Noise (AutoRMS) = 595.35; SNR (1.650min) = 965.1
x10 5
* 1.650
6
4
2
0.4 0.6 0.8 1 1.2 1.4 1.6 1.8 2 2.2 2.4 2.6 2.8 3 3.2 3.4 3.6 3.8 4 4.2 4.4 4.6 4.8
Counts vs. Acquisition Time (min)

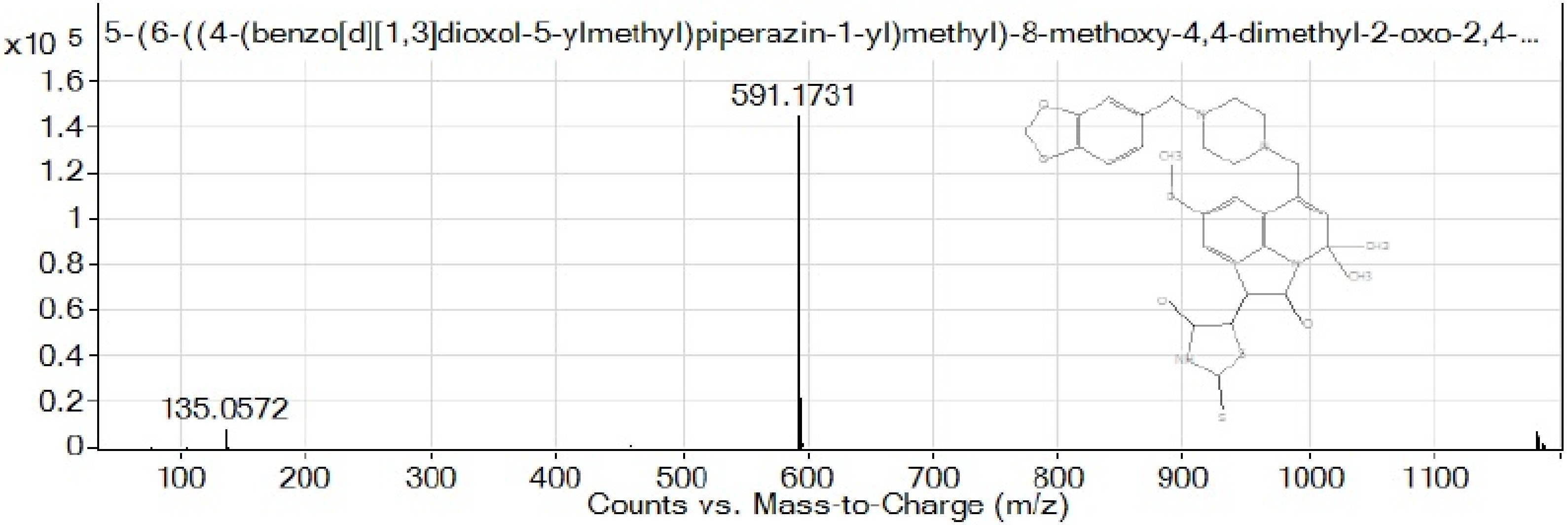

x10 5
5-(6-((4-(benzo[d][1,3]dioxol-5-ylmethyl)piperazin-1-yl)methyl)-8-methoxy-4,4-dimethyl-2-oxo-2,4-...
1.6
1.4
1.2
1
0.8
0.6
0.4
0.2
0
591.1731
135.0572
100 200 300 400 500 600 700 800 900 1000 1100
Counts vs. Mass-to-Charge (m/z)

(*Z*)-5-(6-((4-(4-fluorophenyl)piperazin-1-yl)methyl)-8-methoxy-4,4-dimethyl-2-oxo-2,4-dihydro-1*H*-pyrrolo[3,2,1-*ij*]quinolin-1-ylidene)-2-thioxothiazolidin-4-one 9e

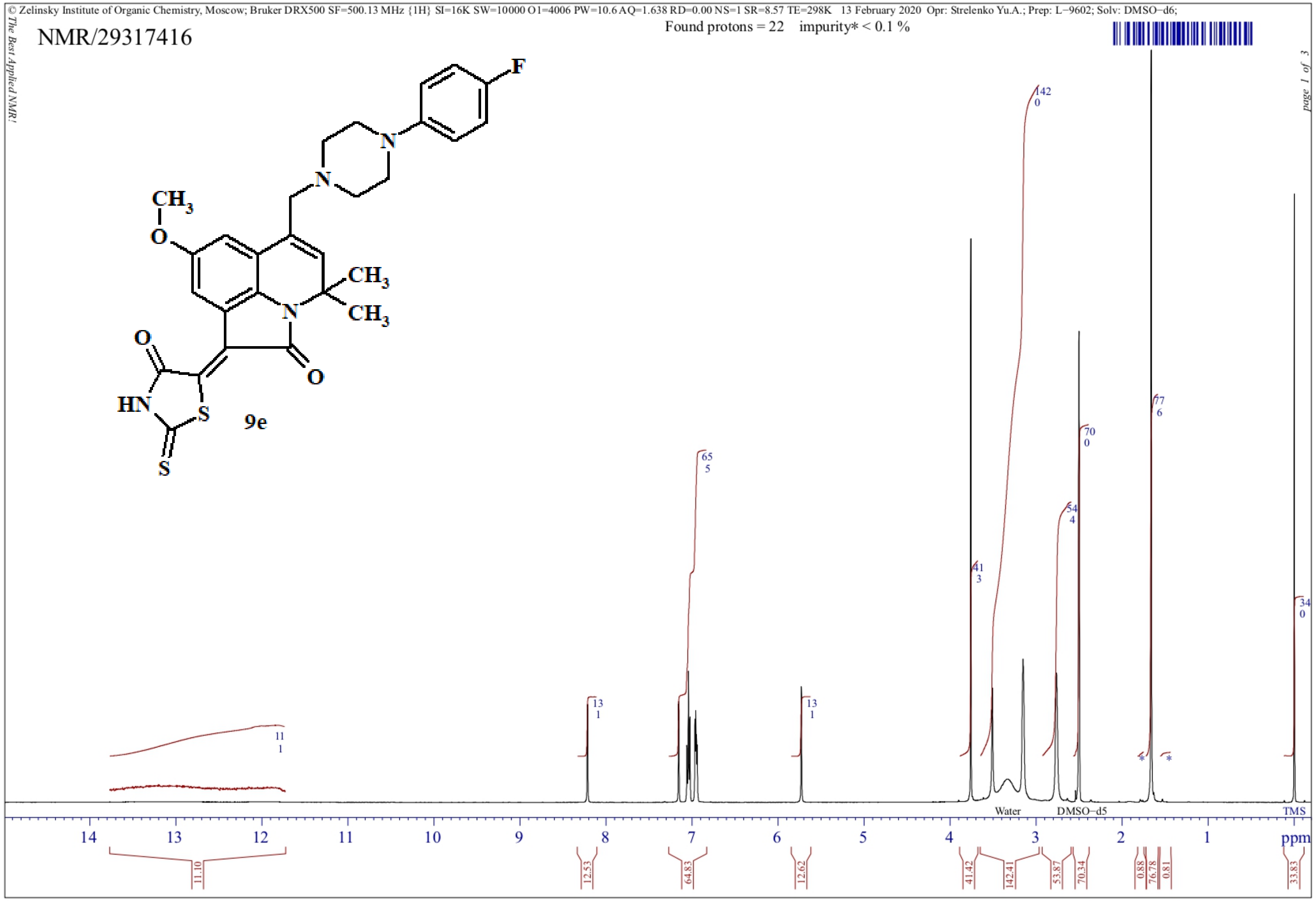

© Zelinsky Institute of Organic Chemistry, Moscow; Bruker DRX500 SF=500.13 MHz {1H} SI=16K SW=10000 O1=4006 PW=10.6 AQ=1.638 RD=0.00 NS=1 SR=8.57 TE=298K 13 February 2020 Opr: Strelenko Yu.A.; Prep: L−9602; Solv: DMSO−d6;

NMR/29317416

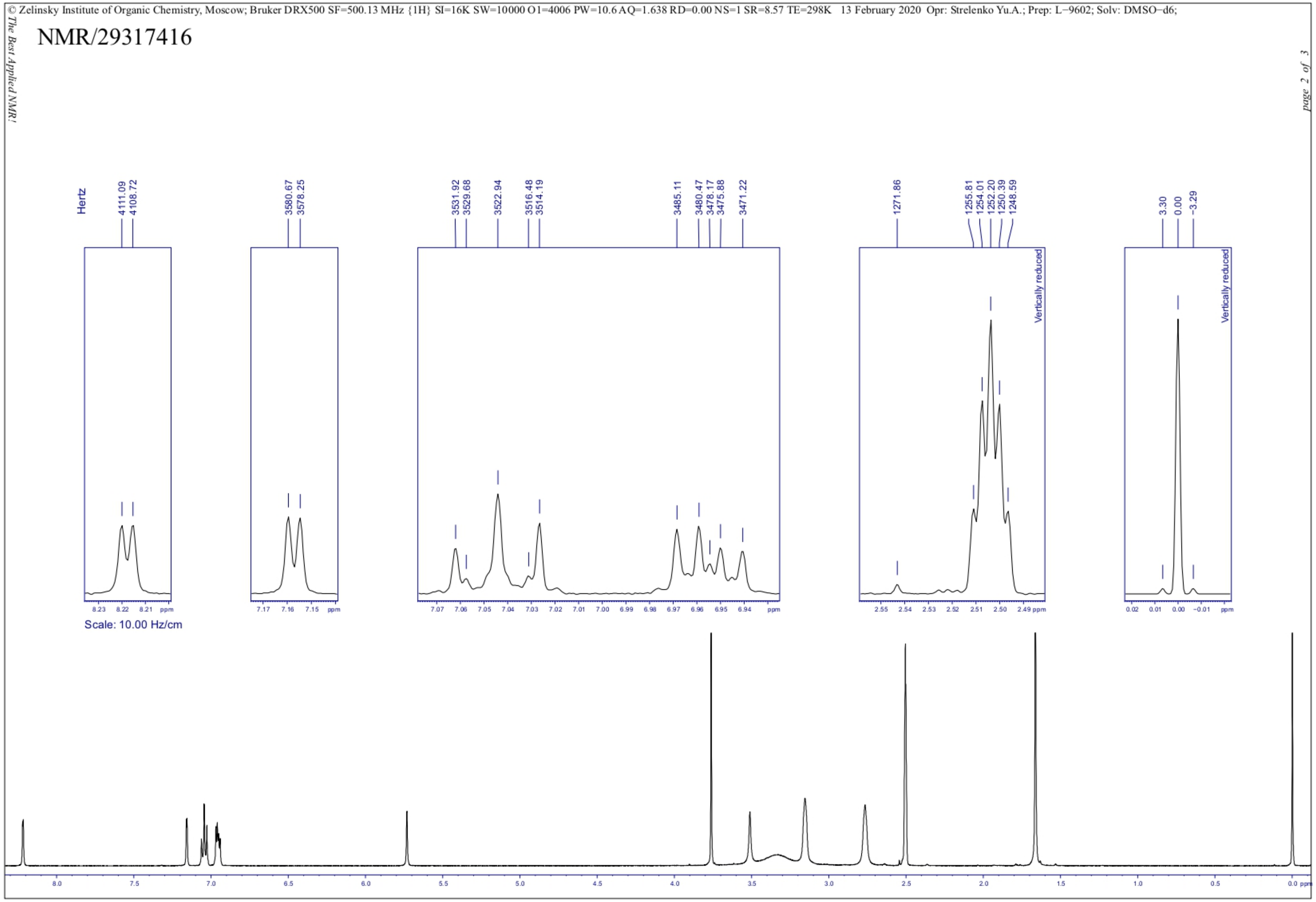

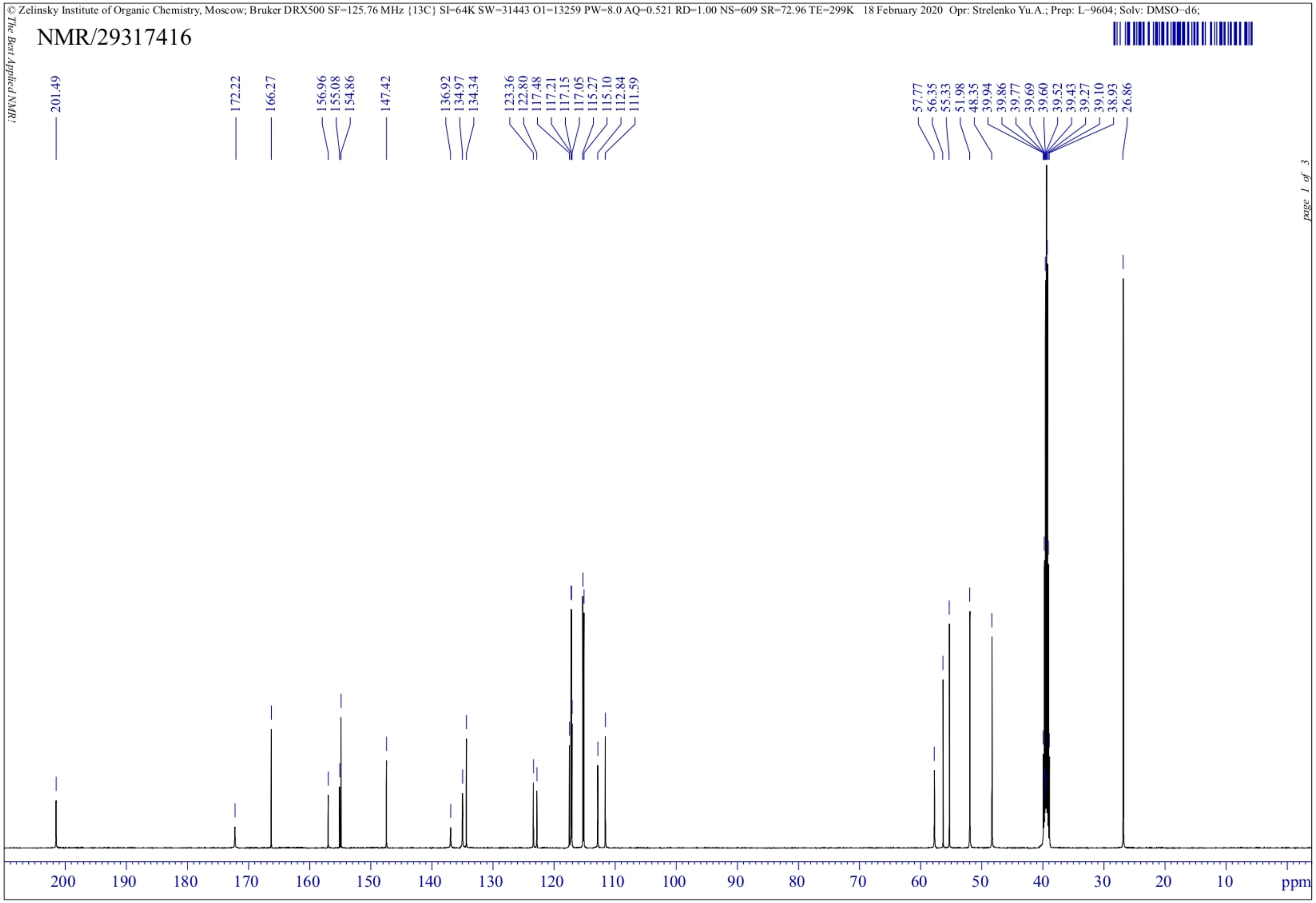
© Zelinsky Institute of Organic Chemistry, Moscow; Bruker DRX500 SF=125.76 MHz {13C} SI=64K SW=31443 O1=13259 PW=8.0 AQ=0.521 RD=1.00 NS=609 SR=72.96 TE=299K 18 February 2020 Opr: Strelenko Yu.A.; Prep: L–9604; Solv: DMSO–d6;
The Best Applied NMR!
NMR/29317416
page 1 of 3
201.49
172.22
166.27
156.96
155.08
154.86
147.42
136.92
134.97
134.34
123.36
122.80
117.48
117.21
117.15
117.05
115.27
115.10
112.84
111.59
57.77
56.35
55.33
51.98
48.35
39.94
39.86
39.77
39.69
39.60
39.52
39.43
39.27
39.10
38.93
26.86
200
190
180
170
160
150
140
130
120
110
100
90
80
70
60
50
40
30
20
10
ppm

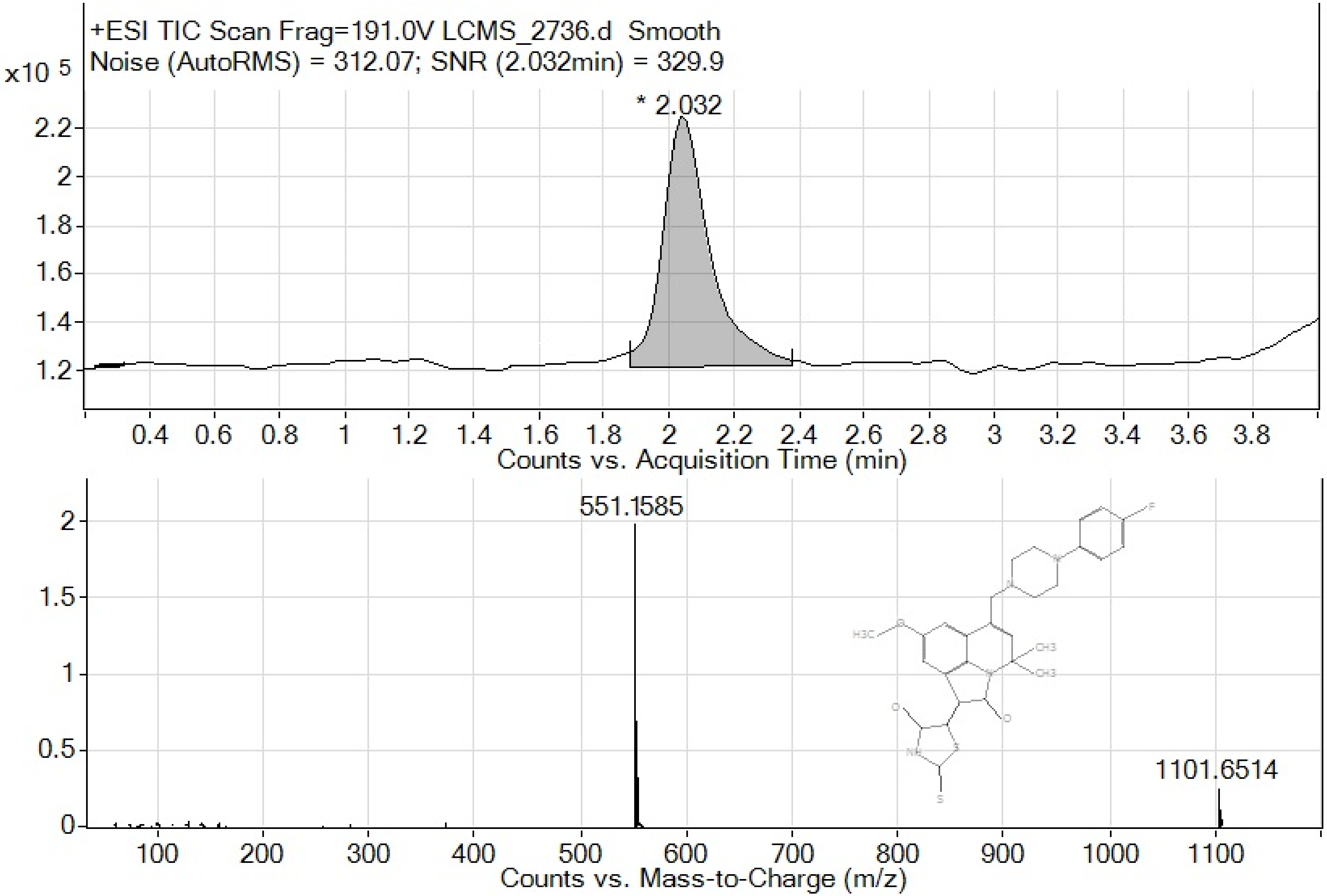
+ESI TIC Scan Frag=191.0V LCMS_2736.d Smooth
Noise (AutoRMS) = 312.07; SNR (2.032min) = 329.9
x10 5
* 2.032
Counts vs. Acquisition Time (min)
551.1585
1101.6514
Counts vs. Mass-to-Charge (m/z)

(*Z*)-5-(6-((4-(benzo[d][1,3]dioxol-5-ylmethyl)piperazin-1-yl)methyl)-8-fluoro-4,4-dimethyl-2-oxo-2,4-dihydro-1*H*-pyrrolo[3,2,1-*ij*]quinolin-1-ylidene)-2-thioxothiazolidin-4-one 9f

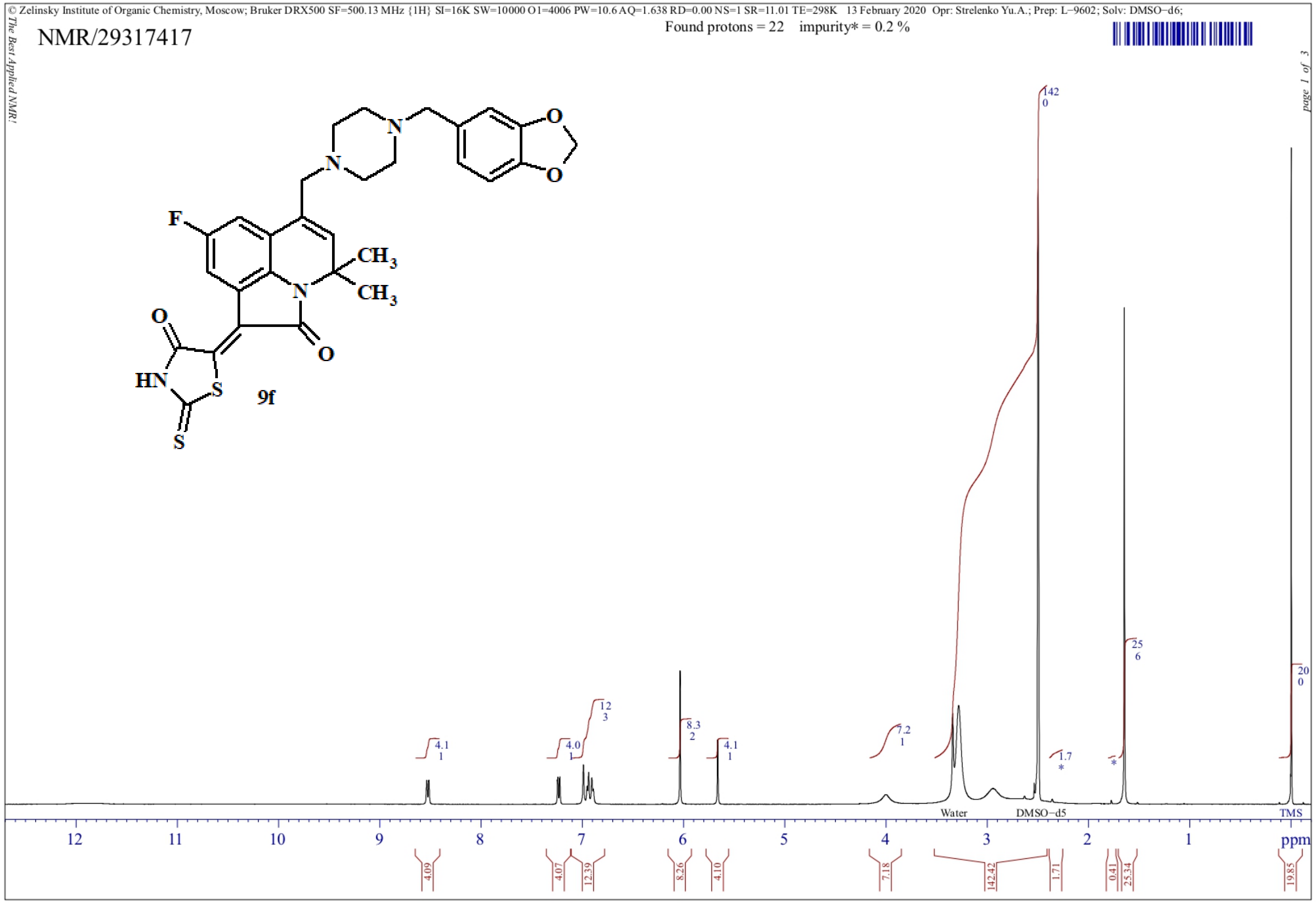

© Zelinsky Institute of Organic Chemistry, Moscow; Bruker DRX500 SF=500.13 MHz {1H} SI=16K SW=10000 O1=4006 PW=10.6 AQ=1.638 RD=0.00 NS=1 SR=11.01 TE=298K 13 February 2020 Opr: Strelenko Yu.A.; Prep: L−9602; Solv: DMSO−d6;

NMR/29317417

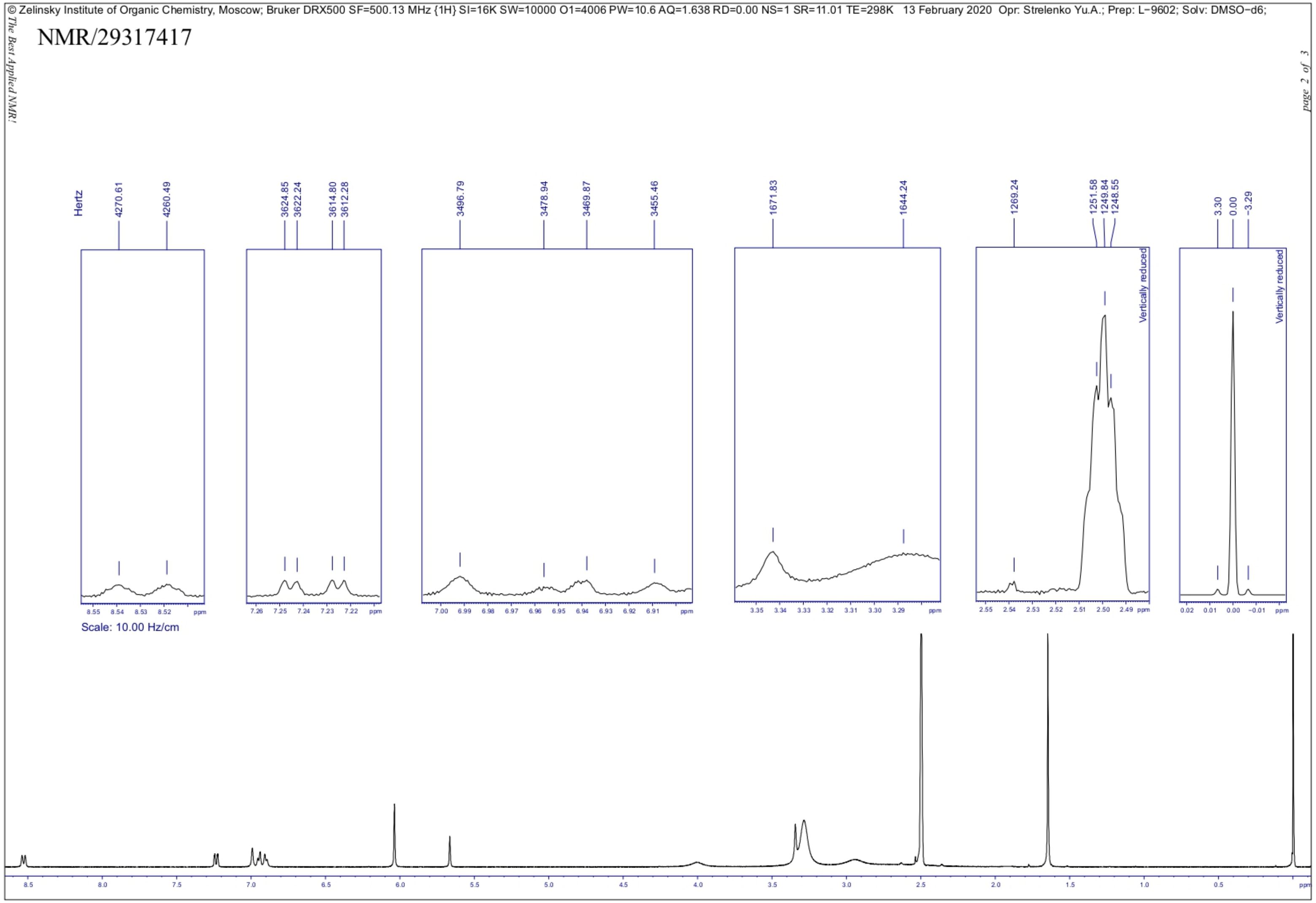

© Zelinsky Institute of Organic Chemistry, Moscow; Bruker DRX500 SF=125.76 MHz {13C} SI=64K SW=31443 O1=13259 PW=8.0 AQ=0.521 RD=2.00 NS=511 SR=54.53 TE=353K 18 February 2020 Opr: Strelenko Yu.A.; Prep: L−9604; Solv: DMSO−d6;

The Best Applied NMR!

NMR/29317417 *Insoluble in DMSO−d6*

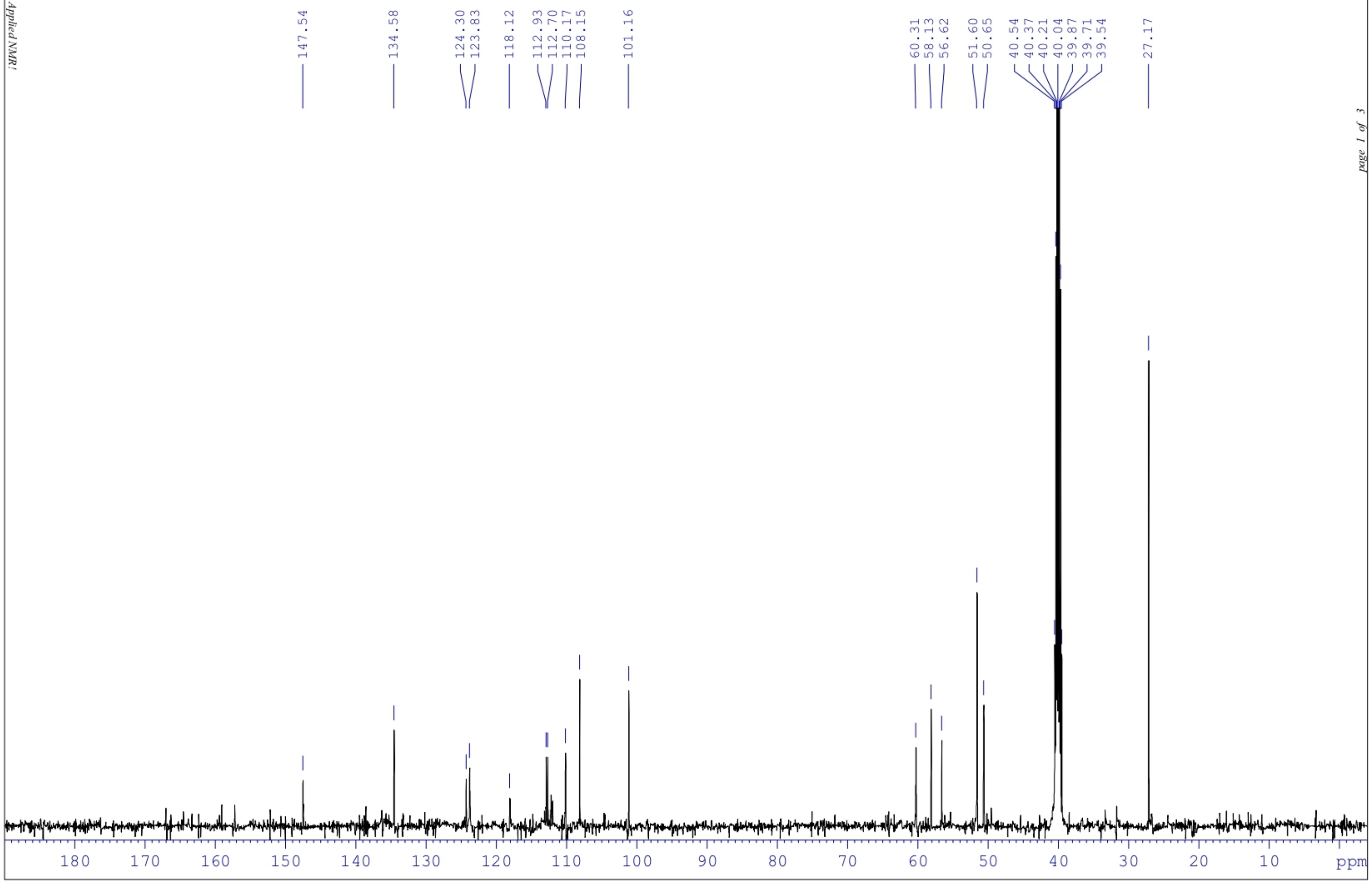

page 1 of 3

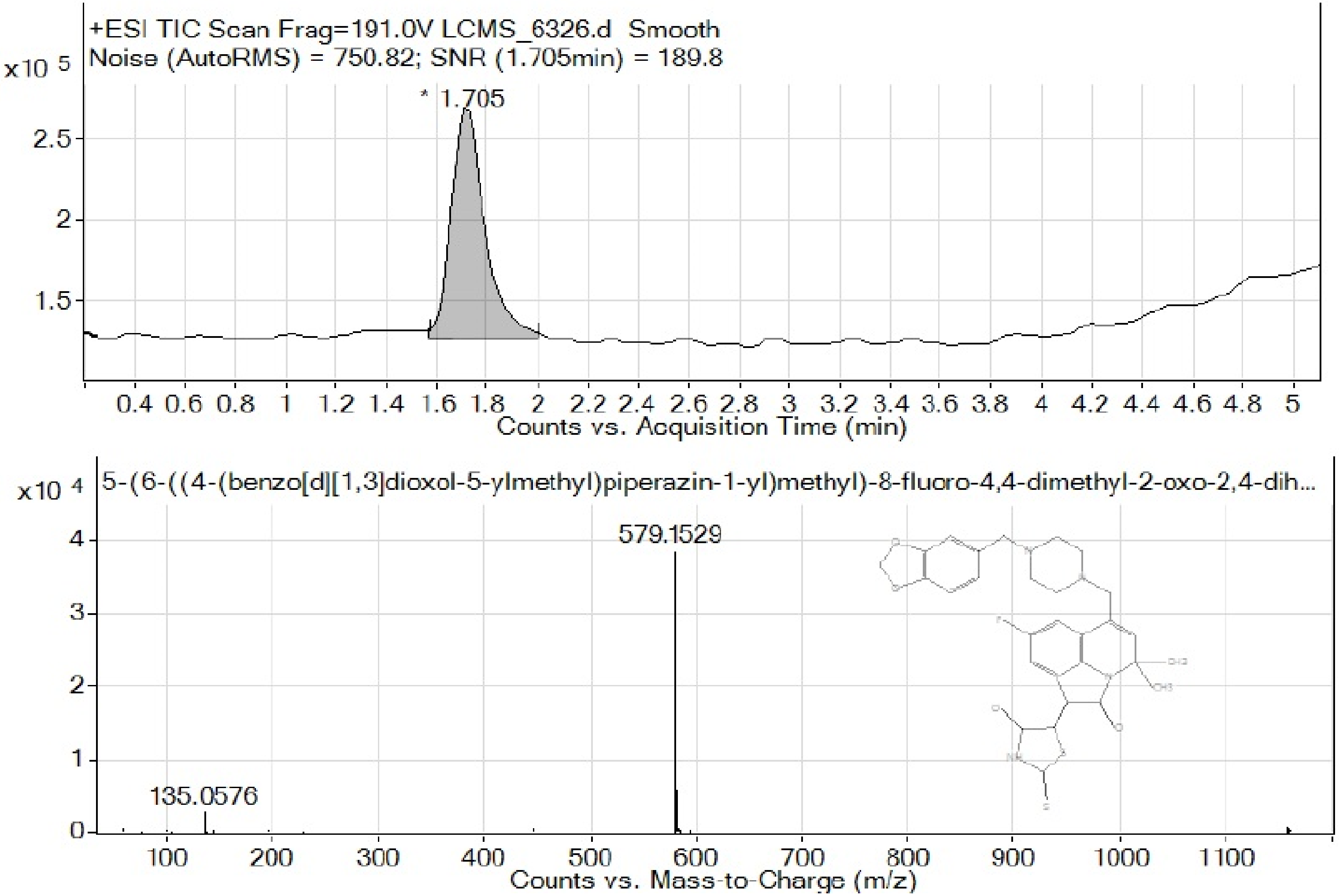

+ESI TIC Scan Frag=191.0V LCMS_6326.d Smooth
Noise (AutoRMS) = 750.82; SNR (1.705min) = 189.8
x10 5
* 1.705
Counts vs. Acquisition Time (min)
x10 4
5-(6-((4-(benzo[d][1,3]dioxol-5-ylmethyl)piperazin-1-yl)methyl)-8-fluoro-4,4-dimethyl-2-oxo-2,4-dih...
579.1529
135.0576
Counts vs. Mass-to-Charge (m/z)
